# Green NiO nanoparticle catalyzed synthesis of novel triazolopyrimidine derivatives with physicochemical characterization and computational evaluation for gout therapy

**DOI:** 10.1038/s41598-026-57449-7

**Published:** 2026-06-26

**Authors:** Doaa A. Elsayed, Wesam S. Shehab, Abdulrahman E. Mesbah, Ahmed F. El-Sayed, Sahar M. Mousa, Gehan T. El-Bassyouni, Basant Farag

**Affiliations:** 1 Department of Chemistry, Faculty of Science, Zagazig University, Zagazig, 44519 Egypt; 2 Department of Chemistry, Faculty of Science, Mansoura University, Mansoura, 35516 Egypt; 3 Microbial Genetics Department, Biotechnology Research Institute, National Research Center, Giza, Egypt; 4 Inorganic Chemistry Department, National Research Center, 33 El-Buhouth St., Dokki, 12622 Cairo Egypt; 5 Refractories, Ceramics and Building Materials Department, National Research Centre, 33 El-Buhouth St., Dokki, 12622 Cairo Egypt

**Keywords:** NiO-nanocatalyzed, Chalcone derivatives, DFT, Docking, ADMET, Dynamics (MD) simulations, Gout, Biochemistry, Chemistry, Computational biology and bioinformatics, Drug discovery

## Abstract

A novel series of triazolopyrimidine derivatives (5a–d and 6a–c) was synthesized via a green NiO nanoparticle-catalyzed protocol under mild conditions, affording the target compounds in good to excellent yields. The NiO nanoparticles exhibited high catalytic efficiency, attributed to their nanoscale features and porous structure, as confirmed by physicochemical characterization. Structural elucidation of the synthesized compounds was achieved using standard spectroscopic techniques. Computational studies, including density functional theory and molecular docking, were employed to evaluate the reactivity and potential multi-target activity of the compounds against key proteins involved in gout pathogenesis. The results revealed that selected derivatives exhibited favourable binding affinities toward multiple inflammatory targets, supported by stable protein–ligand interactions in molecular dynamics simulations. These findings suggest that the synthesized compounds may serve as potential multi-target candidates for gout therapy and warrant further biological investigation.

**Supplementary Information:**

The online version contains supplementary material available at 10.1038/s41598-026-57449-7.

## Introduction

Monosodium urate (MSU) crystals are deposited in joints and periarticular tissues, and persistent hyperuricemia is a hallmark of gout, a chronic inflammatory metabolic disease. Gout was once thought to be a self-limiting arthritic disorder, but it is now understood to be a systemic inflammatory disease that is closely linked to metabolic syndrome, cardiovascular morbidity, chronic renal disease, and increased mortality^1,2^. Gout has become a major public health problem since its prevalence has significantly grown globally over the past few decades, mostly due to aging populations, dietary changes, and an increase in metabolic diseases.

**Fig. 1:**
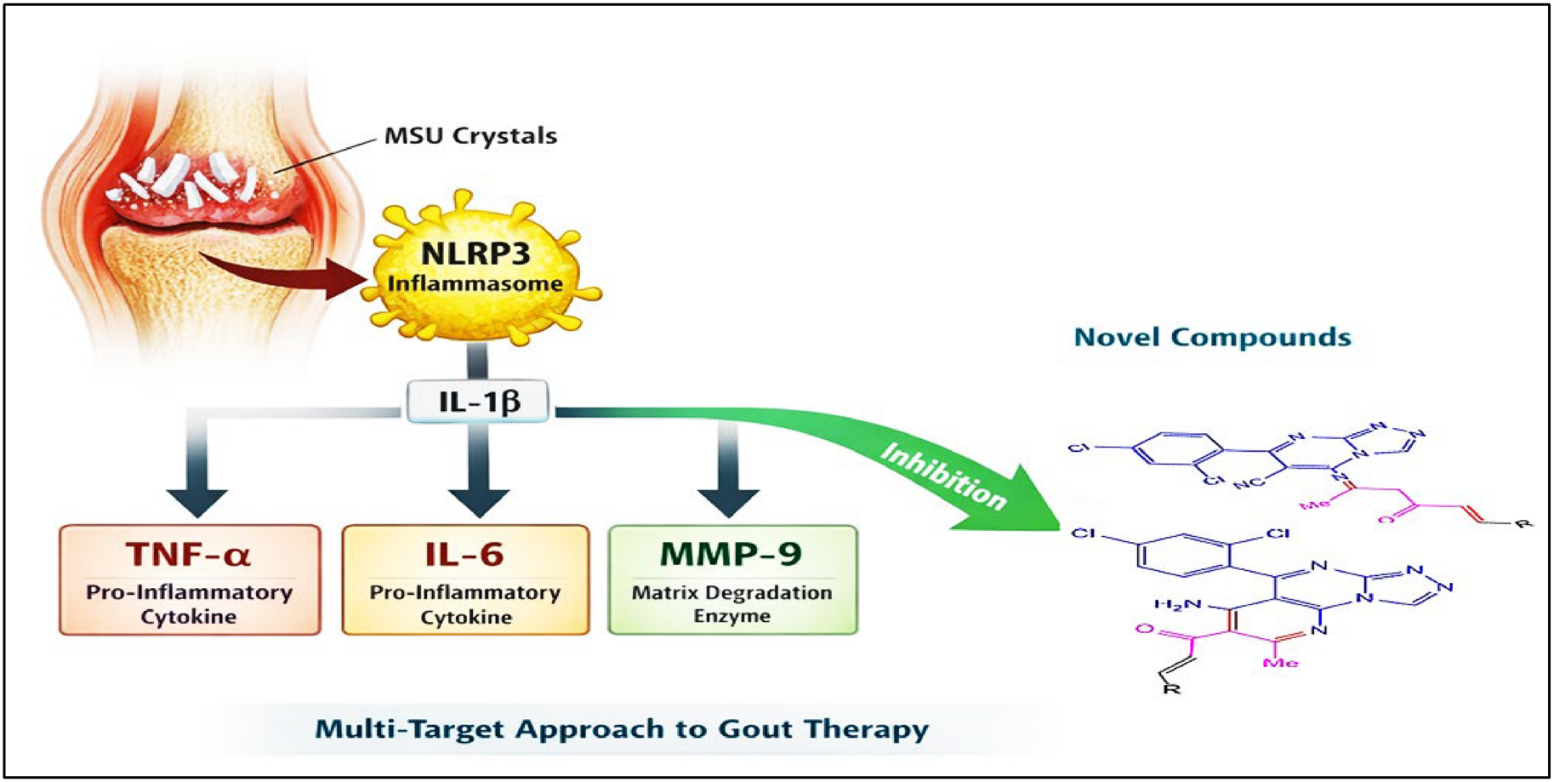
Structured multi-target strategy.

At the molecular level, gout pathogenesis is initiated by MSU crystal deposition, which activates innate immune responses, particularly the NLRP3 inflammasome. Activation of NLRP3 leads to caspase-1-mediated maturation of interleukin-1β (IL-1β), subsequently triggering a cascade of pro-inflammatory mediators, including tumor necrosis factor-alpha (TNF-α), interleukin-6 (IL-6), and matrix metalloproteinases such as MMP-9^3–5^. These mediators contribute not only to acute gout flares but also to chronic joint destruction and systemic inflammation. This inflammatory response is regulated by a complex signaling network rather than a singular pathway, with NLRP3 activation acting as an upstream regulatory hub that enhances downstream cytokine production and enzymatic tissue destruction. TNF-α and IL-6 exacerbate the inflammatory response, whereas MMP-9 facilitates the degradation of the extracellular matrix and joint impairment. Considering this intricate and multifactorial inflammatory cascade, mounting data indicates that focusing on a singular route may be inadequate for successful disease management^6^. A multi-target therapeutic strategy that concurrently inhibits inflammasome activation (NLRP3), suppresses pro-inflammatory cytokines (TNF-α and IL-6), and diminishes tissue degradation (MMP-9) constitutes a more comprehensive and effective method for addressing both acute and chronic manifestations of gout^7^. This comprehensive pathway-based targeting offers a robust mechanistic justification for the selection of these particular proteins in the current investigation (Fig. 1).

Anti-inflammatory drugs and xanthine oxidase inhibitors, such as febuxostat and allopurinol, are the mainstays of conventional gout therapy. Nevertheless, these medications are often linked to negative side effects, renal impairment contraindications, and cardiovascular safety issues^8,9^. Moreover, they primarily target hyperuricemia instead of directly modifying the inflammatory signaling pathways that are essential to the pathogenesis of gout. Therefore, a new and sensible approach to managing gout is the creation of innovative multi-target therapeutic drugs that may concurrently alter inflammatory mediators, including IL-6, TNF-α, MMP-9, and NLRP3^7,10,11^.

The diverse range of biological activities shown by heterocyclic scaffolds, especially fused pyrimidine derivatives, has garnered significant attention in medicinal chemistry. These activities include anti-inflammatory, anticancer, antibacterial, and enzyme inhibitory actions^12–15^. Triazolopyrimidine frameworks are distinctive among these privileged structures due to their broad pharmacological versatility, which results from their ideal electronic distribution, favorable hydrogen-bonding ability, and structural rigidity, all of which enable effective interaction with a variety of biological targets^16^. Furthermore, it has been demonstrated that by enhancing binding affinity inside hydrophobic protein domains and encouraging π–π stacking interactions, structural hybridization with chalcone moieties improves biological performance^17^.

Despite notable progress in gout treatment, existing strategies predominantly concentrate on lowering uric acid levels, failing to adequately tackle the intricate inflammatory network that underpins disease pathogenesis, while the engagement of multiple interrelated signaling pathways constrains single-target methods. Consequently, the advancement of innovative multi-target agents is essential. This study employs a rational design strategy that integrates pharmacologically active scaffolds, selecting the triazolopyrimidine core for its anti-inflammatory and enzyme inhibitory properties, while incorporating chalcone moieties to improve hydrophobic interactions, π–π stacking, and overall binding affinity. This hybrid design is further rationalized by the complementary electronic and structural features of both moieties, which are expected to enhance binding interactions within multiple biological targets associated with gout inflammation. The objective of producing structurally optimized compounds that can modulate multiple targets implicated in gout-related inflammation.

Alongside sensible scaffold design, green Nano catalysis has become a viable and effective method for building these intricate heterocyclic systems with the least possible negative effects on the environment^18,19^. The mesoporous design, large surface area, chemical stability, and high recyclability of nickel(II) oxide nanoparticles (NiO-NPs) in particular make them extremely good heterogeneous catalysts^20,21^. They facilitate multicomponent reactions, including intramolecular cyclization and Knoevenagel condensation under moderate reaction conditions because of their face-centered cubic crystalline structure and nanoscale dimensions, which greatly increase surface reactivity.

Molecular docking is an important computational tool in rational drug design because it allows us to predict how tiny compounds interact with protein targets and assess their binding affinity. This simplifies the drug discovery process by offering mechanistic insights early on. The ongoing development of docking algorithms strives to increase the accuracy of these predictions. Molecular docking was used in this study to investigate the interactions between newly synthesized compounds and specific protein receptors, thereby assessing their proposed mechanisms of action and gout efficacy, as demonstrated in previous studies^22–25^. The HOMO, LUMO, and gap energies of the synthesized compounds were analyzed at the B3LYP/6-31G (d,p) level with RIJCOSX and the CPCM solvent model in order to determine their chemical reactivity. The reactivity descriptors ionization potential (IP), electron affinity (Ea), electronic chemical potential (µ), chemical hardness (η), and chemical softness (S) were calculated from HOMO, LUMO, and gap energies.

**Fig. 2:**
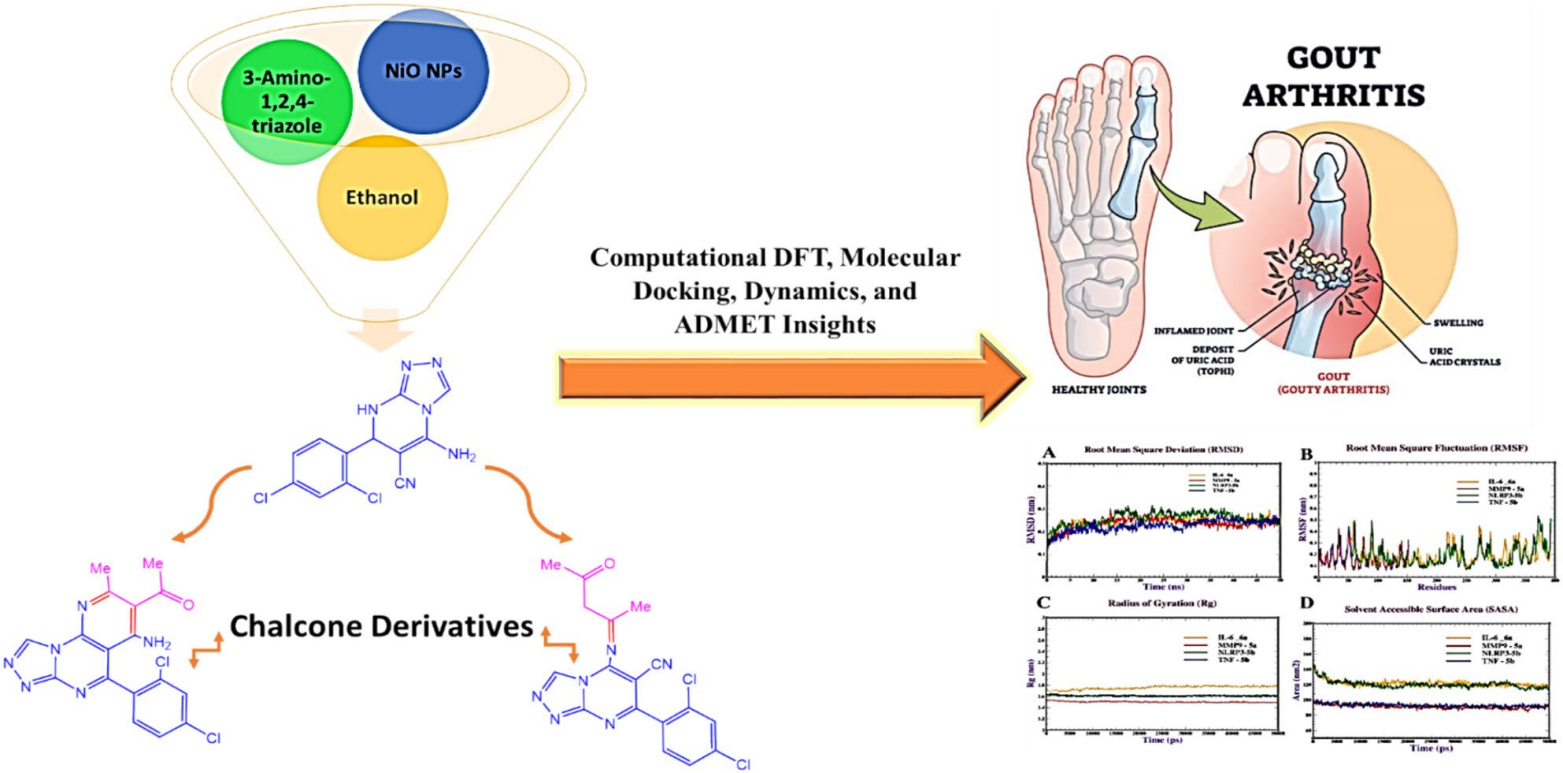
Conceptual framework illustrating the overall workflow of the study, including green NiO nanoparticle-catalyzed synthesis, physicochemical characterization, and computational evaluation of triazolopyrimidine derivatives for gout therapy.

Beyond their function as effective green catalysts, NiO nanoparticles may indirectly increase the biological efficacy of the produced compounds by facilitating the creation of structurally precise and highly functionalized heterocycles with enhanced purity and yield. The nanoscale catalytic environment enables precise regulation of reaction pathways, resulting in optimal molecular characteristics such as improved planarity, conjugation, and advantageous functional group orientation, which are crucial for successful binding to biological targets. Thus, NiO-assisted synthesis facilitates environmentally friendly methods and the production of structurally refined molecules with enhanced pharmacological potential. This study combines green nanocatalysis with strategic scaffold design and multi-target computational analysis, evaluating synthesized triazolopyrimidine derivatives against critical inflammatory mediators involved in gout, such as NLRP3, TNF-α, IL-6, and MMP-9. Molecular docking and molecular dynamics simulations were employed to confirm their binding affinity and stability, thereby endorsing their potential as effective anti-gout agents (Fig. 2).

## Results and discussion

### NiO nanoparticle characterization

#### XRD analyses

**Fig. 3:**
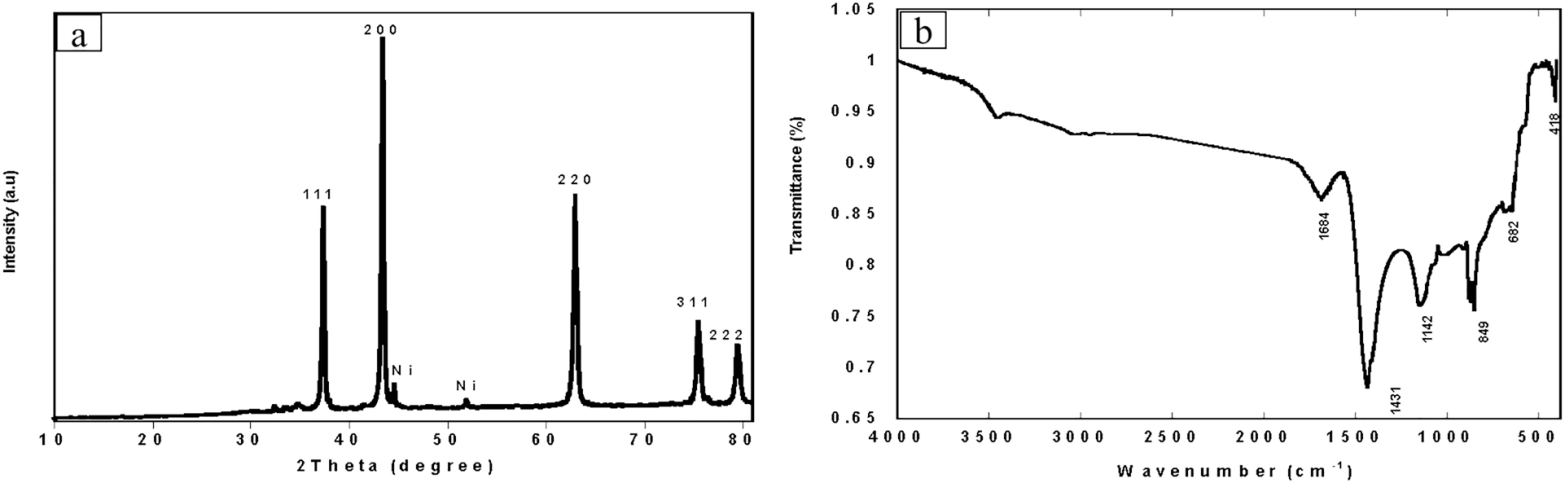
Structural characterization of NiO nanoparticles showing (**a**) the X-ray diffraction (XRD) pattern confirming the crystalline face-centered cubic structure and (**b**) the FT-IR spectrum indicating characteristic Ni–O vibrational bands and surface functional groups.

X-ray diffraction (XRD) analysis unequivocally established the crystalline architecture of the synthesized NiO nanoparticles (NiO-NPs). The diffraction pattern (Fig. 3a) displays a pristine face-centered cubic (FCC) NiO phase, marked by sharp, characteristic peaks at 2θ = 37.325°, 43.357°, 62.940°, 75.462°, and 79.436° perfectly indexed to the (111), (200), (202), (311), and (222) planes (JCPDS #96-101-0096)^26^. Minor, distinct signatures of cubic Ni nanoparticles were also observed at 44.549° and 51.896° (JCPDS 04-850)^27^. Crucially, the complete absence of any extraneous peaks confirms the high phase purity and single-crystal integrity of the prepared NiO nanostructures, validating their suitability for high-performance catalytic applications.

Quantitative analysis of X-ray diffraction peak broadening, using the Debye–Scherrer equation, revealed that the synthesized NiO nanoparticles possess a significantly small average crystallite size of 3.2 nm. This value, derived from the most intense diffraction peak after correcting for instrumental broadening, conclusively confirms the formation of ultrafine, nanoscale crystallites. The resulting high surface-to-volume ratio inherent to this size range provides a direct rationale for the material’s enhanced catalytic performance. Furthermore, this finding is in excellent agreement with complementary morphological analyses, collectively validating the successful synthesis of phase-pure NiO nanostructures with superior surface reactivity.

#### FT-IR analyses

FTIR spectroscopy was employed to probe the vibrational structure and surface characteristics of the prepared NiO nanoparticles (Fig. 3b), which display different vibrational patterns that clarify their lattice structure and surface properties. The two significant bands at 418 and 682 cm^−1^ are attributed to Ni–O stretching vibrations in the face-centered cubic lattice, hence validating the effective synthesis of crystalline NiO^28^. The spectra indicate notable surface activity, as seen by a wide band at 1684 cm^−1^, which is ascribed to the carbonyl (C=O) stretching of chemisorbed CO₂ and the O–H bending of physisorbed water molecules^29,30^. The distinct bands at 1142 and 1431 cm^−1^ correspond to the symmetric and asymmetric stretching vibrations of carboxylate (O–C = O) groups, while the absorption at 849 cm^−1^ is linked to carbonyl stretching modes. The findings demonstrate the significant surface reactivity of nanosized NiO particles, evidenced by their robust interaction with atmospheric moisture and CO_2_, emphasizing their appropriateness for heterogeneous catalysis and environmental sensing applications that rely on surface-mediated processes^31^. This comprehensive vibrational fingerprint confirms the strong affinity of the nanosized NiO for environmental species, validating its potential as a high-performance platform for surface-driven catalytic and sensing technologies.

#### Surface morphology and structural analysis of NiO nanoparticles

**Fig. 4:**
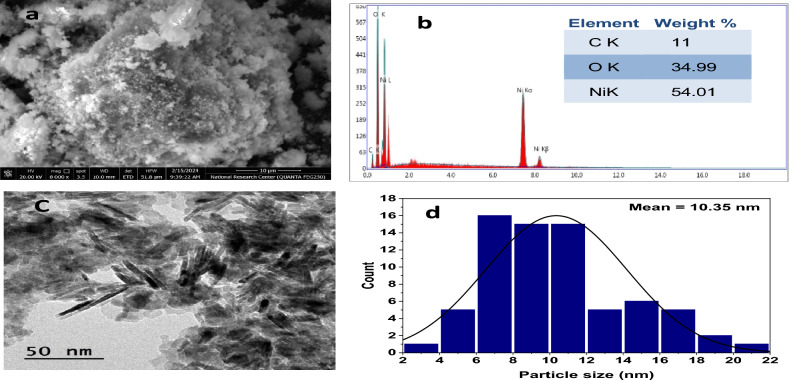
(**a**) SEM micrograph, (**b**) EDX spectrum, (**c**) HR-TEM image, and (**d**) particle size distribution histogram of the synthesized NiO nanoparticles.

The surface morphology of the produced NiO nanoparticles was analyzed using SEM at a magnification of 8000 × (Fig. 4a). The image demonstrates significant agglomeration, with initial nanoparticles forming almost spherical aggregates and dense nanoclusters, a typical characteristic of nanoscale metal oxides^32^. The elemental composition, verified by EDX analysis (Fig. 4b), shows dominant signals for nickel (54.01 wt.%) and oxygen (34.99 wt.%), confirming the expected stoichiometry. Significantly, the 11 wt.% carbon signal substantiates the presence of surface-adsorbed atmospheric CO₂, providing direct evidence for the material’s high surface reactivity and aligning perfectly with the FT-IR findings^29^. High-resolution TEM imaging (Fig. 4c) resolves the internal structure, clearly exposing the cubic geometry of individual crystallites, a morphological confirmation of the crystalline lattice deduced from XRD analysis^33^. The corresponding particle size histogram reveals a sharp, monomodal distribution centered at 10.35 nm**,** underscoring the success of the synthetic protocol in producing uniformly sized, phase-pure NiO nanoparticles are ideally suited for surface-driven applications.

### Textural properties and porosity analysis of NiO nanoparticles

The textural characteristics of the synthesized material were rigorously evaluated via N₂ adsorption–desorption analysis, revealing a highly developed mesoporous architecture. BET surface area calculations conducted within the relative pressure range of P/P₀ = 0.05–0.30 yielded a notably high specific surface area of 139.84 m^2^/g, underscoring the material’s strong potential for surface-mediated applications, including gas adsorption and heterogeneous catalysis. Pore structure analysis employing the Barrett-Joyner-Halenda (BJH) model confirmed an average pore radius of 6.42 nm, firmly establishing the mesoporous nature of the framework. The total pore volume reached 0.404 cm^3^/g at P/P₀ = 0.99366 (for pores below 152.33 nm), with a BJH cumulative pore volume of 0.390 cm^3^/g, reflecting substantial internal void space. This elevated pore volume suggests the presence of a hierarchical pore network, which is expected to significantly enhance mass transport efficiency in catalytic regimes.

**Fig. 5:**
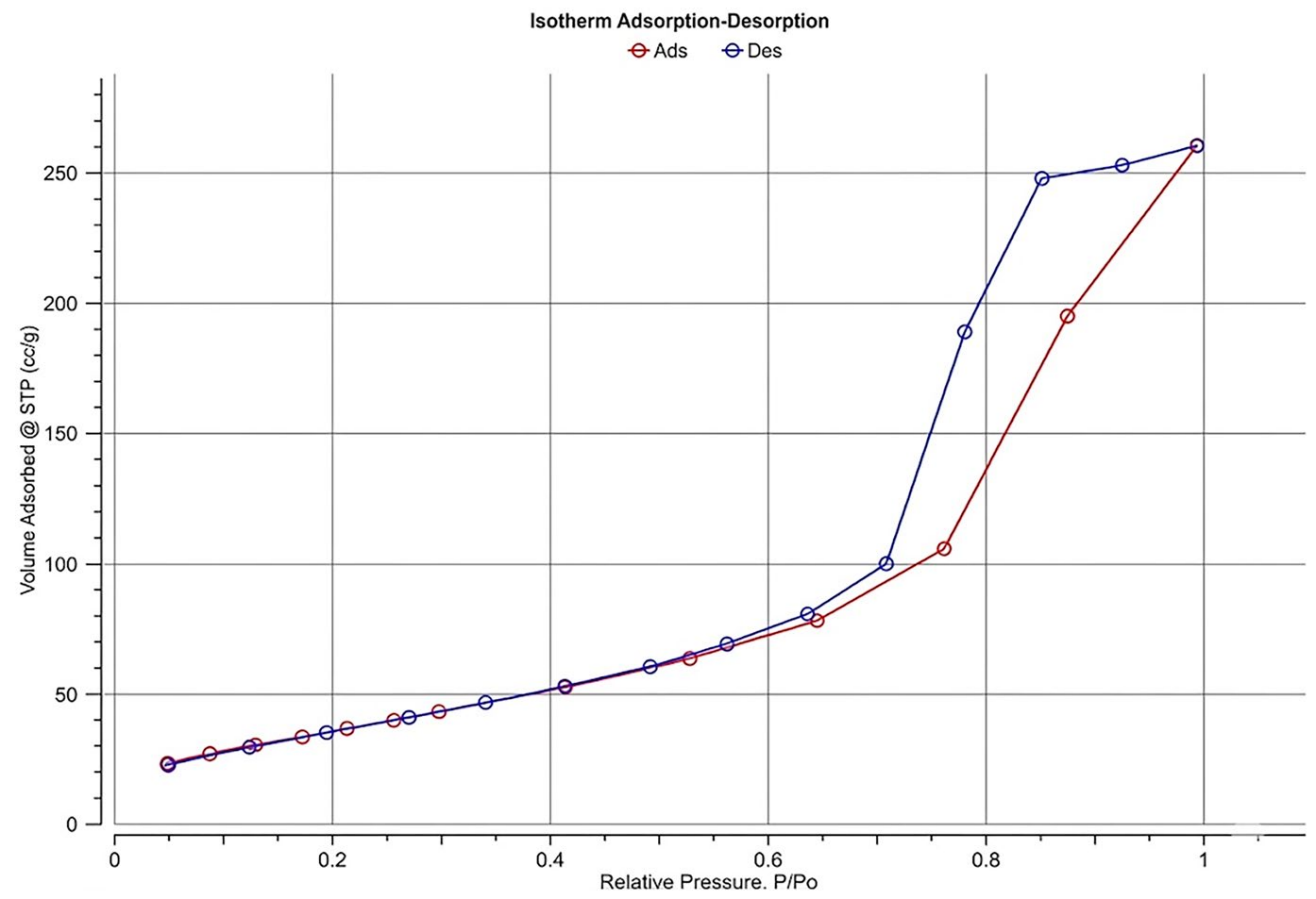
N₂ adsorption–desorption isotherms of NiO nanoparticles, revealing their porous texture and surface characteristics.

As demonstrated in Fig. 5, the isotherm conforms to a Type IV profile with an H1-type hysteresis loop according to IUPAC classification, indicative of cylindrical mesopores with uniform dimensions and a narrow size distribution. The pronounced hysteresis observed in the relative pressure range of 0.45–0.99 confirms capillary condensation within well-defined mesopores, reinforcing the material’s structural order and pore interconnectivity. This distinct separation between adsorption and desorption branches not only attests to the high degree of pore ordering but also highlights the continuous, accessible nature of the pore network, an architectural trait essential for facilitating rapid diffusion and reaction kinetics in advanced catalytic systems.

### Chemistry

**Fig. 6:**
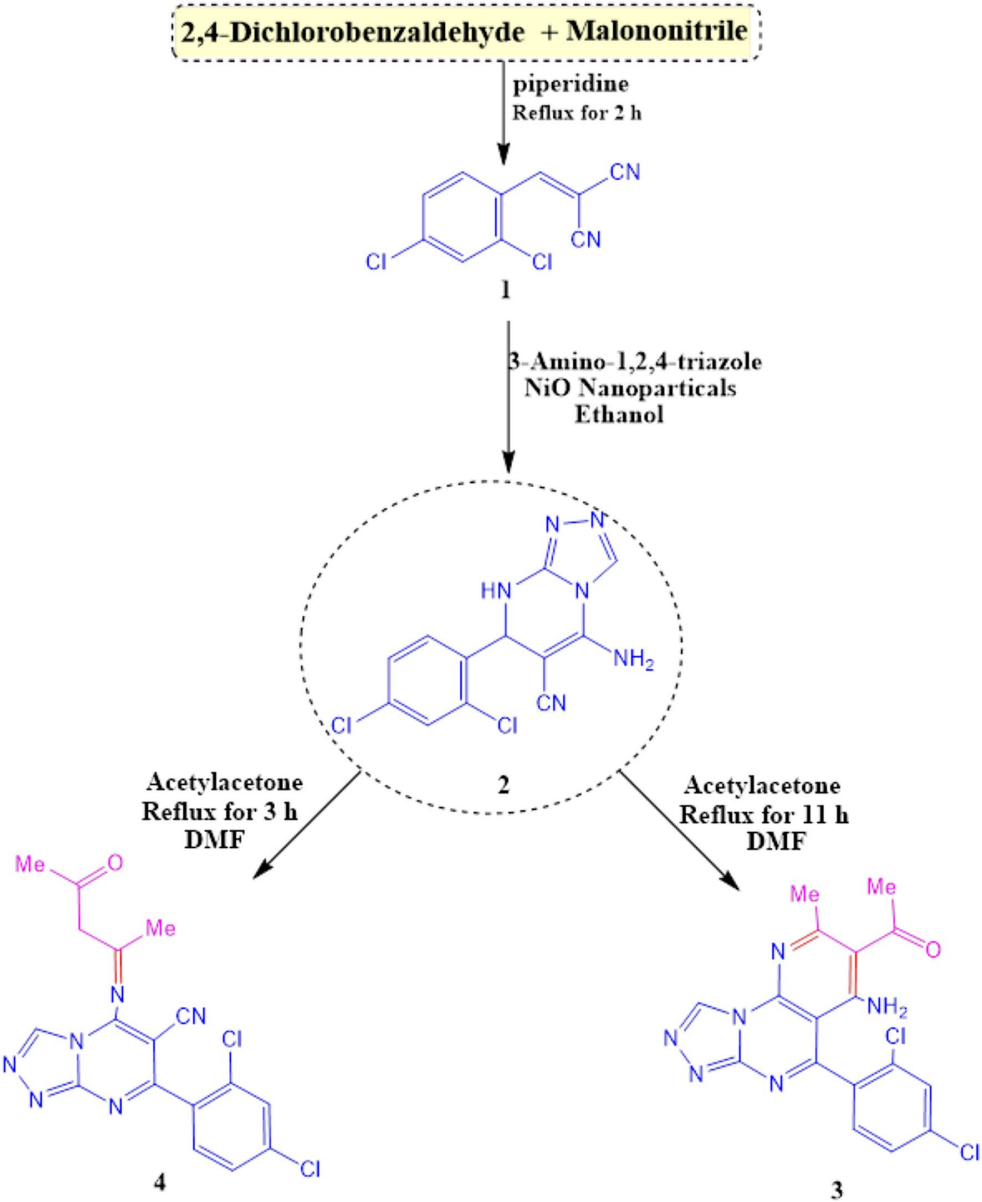
Synthetic pathway for the preparation of triazolopyrimidine derivatives via NiO nanoparticle-catalyzed reactions under green conditions.

The synthetic route commenced with the formation of 2-(2,4-dichlorobenzylidene)malononitrile **(1)** via a base-catalyzed Knoevenagel condensation between 2,4-dichlorobenzaldehyde and malononitrile in the presence of piperidine as a catalyst^34^. The reaction proceeded smoothly under reflux conditions in ethanol, affording compound **1** as a buff-colored crystalline solid in excellent yield (97%). This step introduced a highly electrophilic alkene moiety adjacent to the nitrile groups, which facilitated further nucleophilic attack in the subsequent step. The successful synthesis of compound **(2)**, 5-amino-7-(2,4-dichlorophenyl)-7,8-dihydro-[1,2,4]triazolo[4,3-*a*]pyrimidine-6-carbonitrile, was confirmed by the obtained spectroscopic and analytical data. The presence of a singlet signal at δ = 5.77 ppm in the ^1^H-NMR spectrum corresponds to the methine proton (CH) in the triazolopyrimidine moiety, indicating cyclization. The two broad singlet signals at δ = 7.29 and 8.67 ppm are attributed to the amino (NH₂) and NH protons, respectively, which are exchangeable by D_2_O, providing strong evidence for the successful incorporation of the amino and triazolo functionalities. The aromatic protons of the 2,4-dichlorophenyl ring appeared as a singlet at δ = 7.45 ppm and doublets at δ = 7.64–7.87 ppm, consistent with their expected splitting patterns and coupling constants. Additionally, the presence of a singlet at δ = 7.71 ppm is assigned to the proton on the triazolo ring, supporting the formation of the fused heterocyclic system. Compound **2** was condensed with acetylacetone under reflux in DMF for 11 and 3 h, respectively, to create compounds **3** and **4**. Their spectral data clearly show this structural variance. Both compounds show distinctive absorption bands for the C = O ketone group in the IR spectra, which are located at around 1724 cm^−1^ **3** and 1701 cm^−1^ **4**, respectively. In contrast to compound **3**, compound **4** has a clear CN stretching band at 2219 cm^−1^, indicating the presence of a nitrile group. Additionally, compound **3** exhibits NH₂ stretches at 3435 and 3368 cm^−1^. Compound **3** contains two methyl singlets at δ = 2.09 and 2.50 ppm, which have also been proven to exist using the same type of analysis but with chloroform as the solvent in the ^1^H-NMR investigations, and a broad singlet at δ = 7.30 ppm for the NH₂ protons, which are exchangeable with D₂O. On the other hand, compound **4** confirms the suggested enaminone structure by exhibiting methyl signals at δ = 2.15 and 2.21 ppm as well as a distinctive methylene (CH₂) singlet at δ = 2.63 ppm. Furthermore, the triazolo proton (CH) in both compounds **3** and **4** exhibits a singlet at δ = 9.45 and 7.94 ppm, respectively; however, because of the different electronic surroundings, its chemical shift varies significantly. The nitrile carbon, which is missing from compound **3**, is responsible for the signal seen at δ = 105.34 ppm in compound **4**'s ^13^C-NMR spectrum. Furthermore, compound **4** confirms the existence of the CH₂ linker by displaying a high-field methylene carbon signal at δ = 46.81 ppm. However, compound **3** shows signals for the two methyl groups at δ = 21.64 and 29.79 ppm and δ = 78.45 ppm, which most likely correspond to the carbon containing the amino group. The appearance of a CN band in IR, the absence of NH₂ protons, the methylene CH₂ signal in ^1^H-NMR, and the CN carbon in ^13^C-NMR in compound **4** provide compelling spectral evidence for the successful formation of the enaminone structure, clearly distinguishing it from compound **3**, which retains the amino functionality (Fig. 6).

The reaction conditions were initially optimized using various catalysts, as summarized in Table 1. Among the tested catalysts, NiO nanoparticles afforded the highest yield (98%) within 35 min under reflux in ethanol. Mn_3_O_4_ nanoparticles also exhibited excellent catalytic performance, providing a comparable yield (96%) under the same conditions, highlighting their potential as efficient nanocatalysts for this transformation. In contrast, traditional organic bases such as NaOH^35^, piperidine, pyridine, and triethylamine required longer reaction times (up to 6 h) and gave lower yields (60–86%), confirming the superior performance of metal oxide nanocatalysts, particularly NiO and Mn_3_O_4_. Although Mn_3_O_4_ showed a yield comparable to that of NiO, NiO was considered superior due to its shorter reaction time, higher catalytic efficiency, and better overall performance under identical reaction conditions.

**Table 1:** Optimization of the reaction conditions.

No	Catalyst	Hours	Yield	Temp	Solvent	Reference for preparing the catalyst
1	NiO	35 min	98%	Reflux	Ethanol	This work
2	Mn_3_O_4_	1.13	96%	Reflux	Ethanol	This work
3	NaOH	2.8	81%	Reflux	Ethanol	^35^
4	Piperidine	2.3	86%	Reflux	Ethanol	This work
5	Pyridine	2.4	79%	Reflux	Ethanol	This work
6	Triethylamine	6	60%	Reflux	Ethanol	This work

**Fig. 7:**
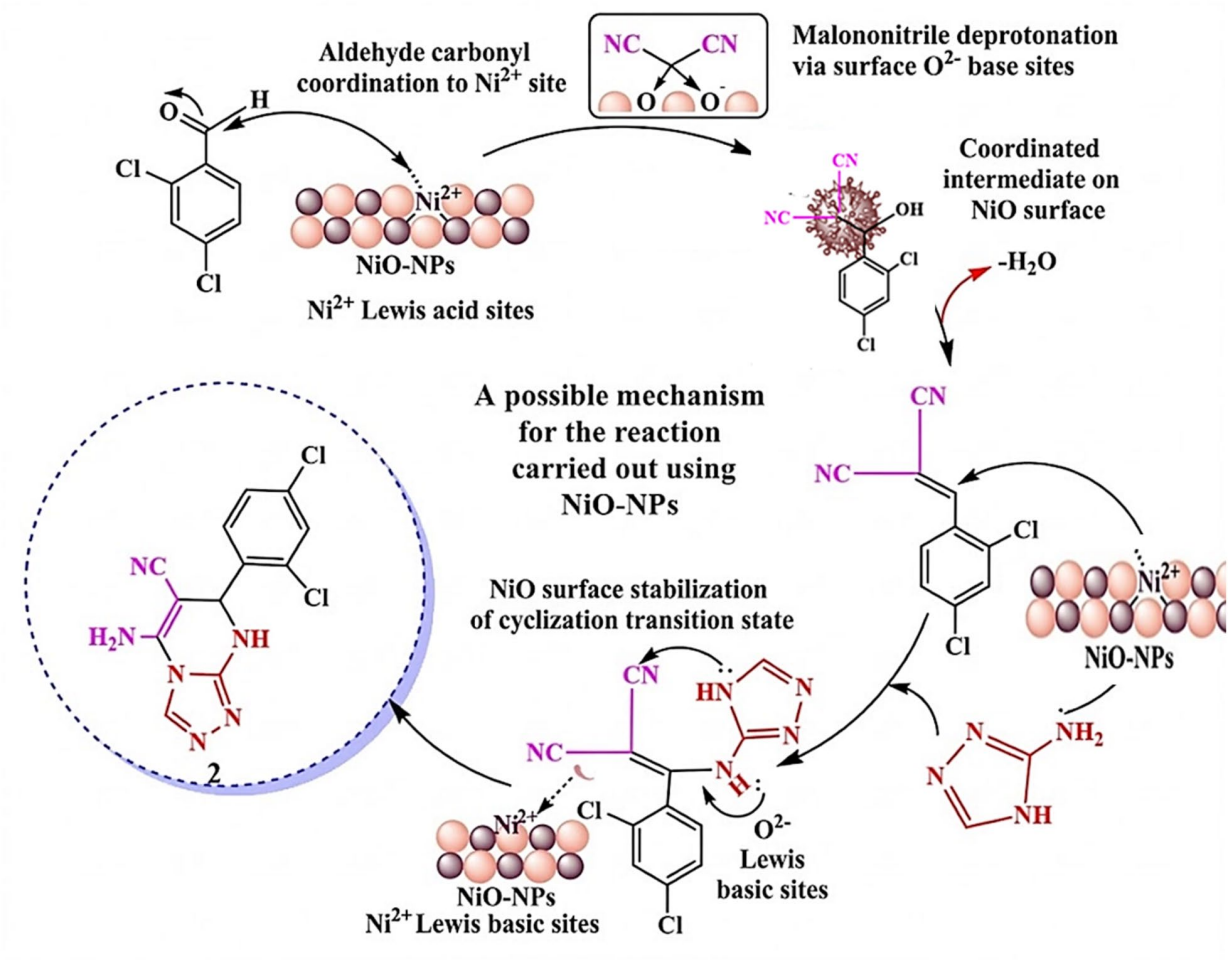
The reaction mechanism in the presence of NiO-NPs.

A detailed mechanistic rationale for the catalytic activity of NiO-NPs is proposed, moving beyond general surface area arguments to incorporate specific Lewis acid–base interactions. The reaction is envisioned to occur via a concerted, surface-mediated multi-step cascade. We propose that the initial Knoevenagel condensation is initiated by dual activation: surface Lewis acidic Ni^2+^ centers coordinate with the carbonyl oxygen of the benzaldehyde, enhancing its electrophilicity (LUMO activation), while simultaneously, adjacent surface Lewis basic O^2−^ sites facilitate the deprotonation of the active methylene protons of malononitrile (H-bond activation or formal deprotonation), as detailed in the revised Fig. 7. This cooperative chemisorption model significantly lowers the activation energy for nucleophilic attack. The subsequent dehydration of the β-hydroxy intermediate to form the arylidene malononitrile is likely assisted by the surface acid sites, which coordinate with the leaving hydroxyl group. The final fused heterocyclic product is formed via a Michael-type addition of the triazole followed by intramolecular cyclization. Throughout this sequence, NiO-NPs provide not only a large reactive surface but also specific catalytic motifs that stabilize ionic transition states and facilitate proton transfers, thus explaining the accelerated reaction rates and high yields observed^36–38^.

The recyclability of the nickel (II) oxide was evaluated over five successive cycles. After completion of each reaction, the catalyst was separated by hot filtration, washed thoroughly with ethanol, and dried before being reused in the next run. As shown in Table 2, the catalyst exhibited excellent reusability, maintaining high catalytic performance over multiple cycles. The yield decreased slightly from 98% in the first cycle to 85% in the fifth cycle. This minor reduction in activity may be attributed to partial aggregation of nanoparticles or blockage of active sites during repeated use. These results demonstrate the good stability and reusability of NiO as an efficient heterogeneous nanocatalyst.

**Table 2:** Reusability of Nickel (II) oxide.

Cycle no.	Yield (%)
1	98
2	97
3	96
4	91
5	85

To further confirm the heterogeneous nature of the catalyst, a hot filtration test was carried out. The catalyst was removed from the reaction mixture by hot filtration after 10 min, and the filtrate was allowed to react further under identical conditions. No additional product formation was observed, indicating that the reaction does not proceed in the absence of the solid catalyst.

This confirms that the catalytic activity originates from the surface of the Nickel(II) oxide, with no contribution from leached active species.

**Fig. 8:**
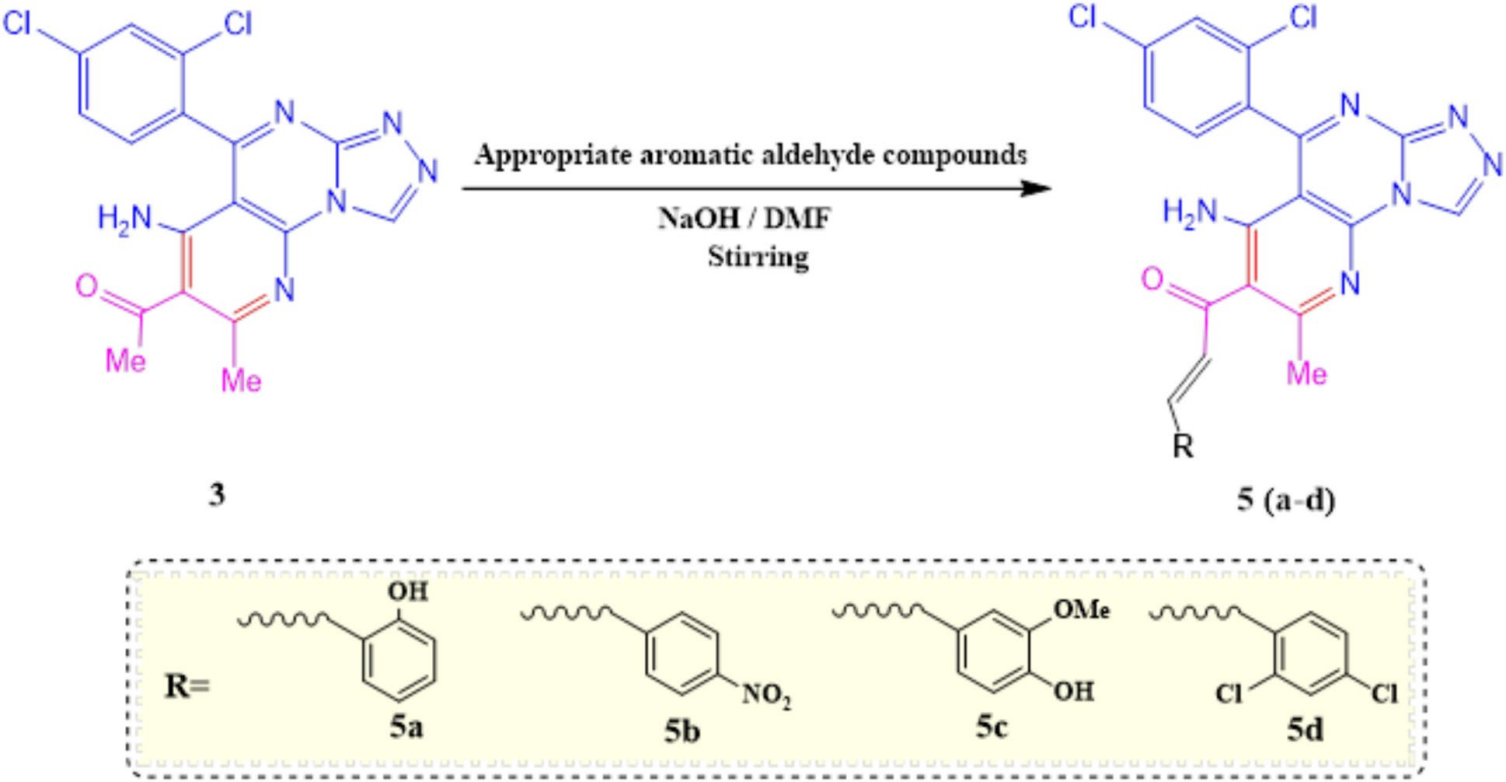
Production of the chalcone derivatives.

The development of novel and distinctive spectrum characteristics that were not present in the initial ketone molecule **3** amply demonstrated the effective production of the chalcone derivatives **5a–d** (Fig. 8). The *α*,*β*-unsaturated carbonyl group (C=O), a crucial indicator of the chalcone moiety, was represented by a prominent and distinct absorption band that emerged in the IR spectra of all final products between 1712 and 1738 cm^−1^. Furthermore, compound **5a** had a wide OH stretch at 3486 cm^−1^, whereas compound **5c** demonstrated both OH (3448 cm^−1^) and CH₃ bands (about 2851–2928 cm^−1^). Compound **5b** showed strong bands at 1547 and 1376 cm^−1^, which are indicative of the NO₂ group and show that an aryl moiety that has been nitro-substituted is present. All final compounds exhibited novel downfield doublets in the ^1^H-NMR spectra, which corresponded to the vinylic protons (CH=CH) of the chalcone system. Additionally, certain aromatic substitution patterns produced other signals, as compound **5c**'s phenolic OH at δ 9.74 ppm and methoxy singlet at δ 3.82 ppm. In addition, the ^13^C-NMR spectrum of the novel derivative **5d** showed δ 34.82 (CH_3_), 113.69–140.23 (vinylic and 12Ar-C), and 191.39 (C=O) ppm. With molecular ion peaks at m/z = 491, 520, 521, and 544 for these novel compounds **5a–d**, respectively, in agreement with the anticipated molecular formulas, mass spectrometric analysis provided additional support for their structures. These recently discovered spectrum indications are unmistakable proof of effective chalcone synthesis and structural diversification from parent compound **3**, especially the conjugated C = O in IR and the enone protons in NMR analyses. 2D/3D structures, melting points, and yields of the prepared compounds (**5a–d**) **(**Table 3**).**

**Table 3:** 2D/3D structures, melting points, and yields of the prepared compounds **5a–d**.

Comp	2D	3D	M.P. °C	Yield %
5a			264–266	82
5b			256–260	93
5c			250–252	86
5d			254–258	89

**Fig. 9:**
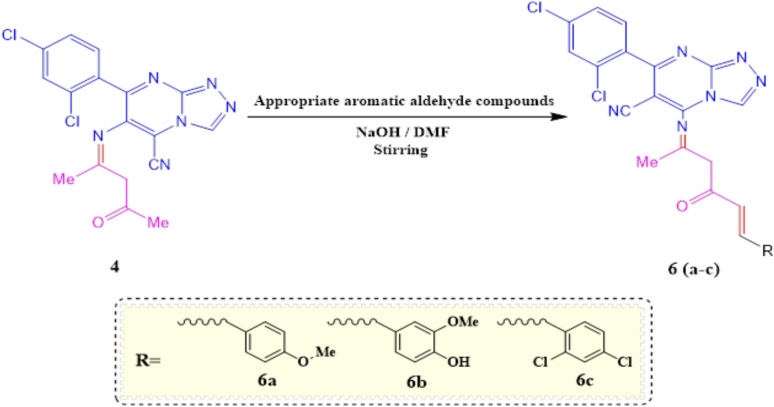
Base-catalyzed Knoevenagel condensation for the synthesis of a new series of triazolopyrimidine derivatives (6a–c).

A base-catalyzed Knoevenagel condensation procedure was used to effectively create a new series of triazolopyrimidine derivatives **6a–c** (Fig. 9). In the presence of DMF as a solvent and NaOH as a basic catalyst, compound **4**, which has an active methylene group next to a ketone, condensed with a number of substituted aromatic aldehydes, including 4-methoxybenzaldehyde, 4-hydroxy-3-methoxybenzaldehyde, and 2,4-dichlorobenzaldehyde. To promote full crystallization, the reaction mixture was stirred for 7–21 h in an ice bath before being left at a low temperature (in a refrigerator) for two weeks. The target compounds **6a-c** were obtained in good to excellent yields by filtering, drying, and recrystallizing the resultant products from methanol. Black crystals of compound **6a**, which was produced from 4-methoxybenzaldehyde, with a melting point of 242–246 °C and a high yield of 90%. Its FT-IR spectrum revealed distinctive absorption bands at 1696 cm^−1^ (C=O ketone), 2250 cm^−1^ (C≡N nitrile), 2935 cm^−1^ (C–H aliphatic), and 3074 cm^−1^ (C–H aromatic). Its singlets for methyl (δ 2.73 ppm), methylene (δ 2.89 ppm), and methoxy protons (δ 3.86 ppm) as well as aromatic and vinylic protons between δ 7.01–7.95 ppm were seen in the ^1^H-NMR spectrum. While the ^13^C-NMR spectrum validated the anticipated carbon surroundings, mass spectrometry showed a molecular ion signal at m/z 505. The computed values (C, H, and N) were in good agreement with elemental analysis. The brown crystals of compound **6b**, which was created using 4-hydroxy-3-methoxybenzaldehyde, had a melting point of 248–250 °C and an 88% yield. Along with peaks for C–H, C≡N, and C=O, the FT-IR spectrum had a wide band at 3431 cm^−1^ that was suggestive of hydroxyl (OH) stretching. Its methyl, methylene, methoxy, and aromatic protons were all detected in the ^1^H-NMR spectrum, along with a singlet at δ 9.77 ppm that indicated a phenolic OH group. The suggested structure was validated by the ^13^C-NMR and MS data (m/z = 521). Additionally, the findings of elemental composition were almost identical. With a melting point of 240 °C, compound **6c**, which was produced by condensation with 2,4-dichlorobenzaldehyde, was separated as pale brown crystals in 72% yield. The IR spectrum displayed distinctive bands for C = O, C≡N (2240 cm^−1^), and aromatic/aliphatic C–H. Its methyl (δ 2.73 ppm), methylene (δ 2.89 ppm), vinylic (δ 6.62–6.66 ppm), and aromatic protons were detected in the ^1^H-NMR spectrum. The proton on the triazolopyrimidine ring was represented by a singlet at δ 8.65 ppm. The elemental analysis results were in agreement with the suggested formula, and the mass spectrum revealed a molecular ion at m/z 544. Physicochemical data and yields of the synthesized chalcone derivatives **6a-c** (Table 4).

**Table 4:** Physicochemical data and yields of the synthesized chalcone derivatives **6a-c**.

Comp	2D	3D	M.P °C	Yield %
6a			242–246	90
6b			248–250	88
6c			240	72

To facilitate structural verification and improve readability, the key spectroscopic data of representative compounds (**3–6**) are summarized in Table 5.

**Table 5:** Key spectroscopic data for compounds **3–6**.

Compound	IR (cm^−1^, key bands)	^1^H NMR (δ, ppm)	^13^C NMR (δ, ppm)	MS (m/z, M⁺)
3	3435, 3368 (NH₂), 1724 (C=O), 794 (C–Cl)	2.09, 2.50 (s, 6H, 2CH_3_), 7.30 (s, 2H, NH_2_), 9.45 (s, 1H, CH _Triazolo_)	21.64, 29.79, 78.45, 114.73, 128.20, 129.58, 132.15, 132.65, 135.57, 135.92, 151.16, 155.23, 156.30, 158.51, 163.24, 167.89, 194.63	387
4	3436 (NH), 2219 (C≡N), 1701 (C=O), 778 (C–Cl)	2.15, 2.21 (s, 6H, 2CH_3_), 2.63 (s, 2H, CH_2_), 7.94 (s, 1H, CH _Triazolo_)	15.36, 30.34, 46.81, 105.34, 128.21, 129.65, 132.38, 135.75, 139.34, 142.08, 148.46, 155.08, 156.59, 165.30, 169.55, 203.0	387
5a	3486 (OH), 3450–3422 (NH₂), 1722 (C=O)	1.66 (s, 3H, CH_3_), 7.45–7.50 (d, 2H, 2CH), 8.53 (s, 1H, CH _Triazolo_)	–	491
5b	3344, 3321 (NH₂), 1547, 1376 (NO₂), 1726 (C=O)	3.81 (s, 3H, CH_3_), 7.92–7.99 (d, 4H, Ar–H), 9.73 (s, 1H, CH _Triazolo_)	–	520
5c	3448 (OH), 1738 (C=O), 647 (C–Cl)	3.82 (s, 3H, OCH_3_), 8.55 (s, 1H, CH _Triazolo_), 9.74 (s, 1H, OH)	31.15, 42.99, 114.98, 115.28, 123.53, 127.36, 128.14, 128.32, 130.43, 131.64, 131.93, 133.48, 136.16, 140.69, 140.87, 143.34, 150.69, 151.57, 152.78, 161.48, 168.66, 197.10	521
5d	3394 (NH₂), 1712 (C = O), 647 (C–Cl)	1.24 (s, 3H, CH_3_), 7.63–7.68 (d,1H, CH), 8.68 (s, 1H, CH _Triazolo_)	34.82, 108.91, 111.02, 113.69, 116.03, 118.47, 119.97, 120.30, 126.73, 128.70, 132.75, 136.20, 138.51, 140.23, 144.72, 148.84, 149.64, 154.07, 158.54, 161.53, 191.39	544
6a	3074 (Ar–CH), 2935 (aliphatic CH), 2250 (C≡N), 1696 (C = O), 639 (C–Cl)	2.73 (s, CH₃), 2.89 (s, CH₂), 3.86 (s, OCH₃), 7.61–7.66 (d, 2H), 9.87 (s, CH _Triazolo_)	31.25, 34.89, 56.17, 114.28, 114.99, 118.43, 123.52, 126.77, 130.11, 131.35, 131.81, 132.29, 136.79, 140.81, 146.42, 154.33, 156.33, 158.81, 160.71, 164.52, 191.82	505
6b	3431 (OH), 3069 (Ar–CH), 2920 (aliphatic CH), 2244 (C≡N), 1669 (C = O), 648 (C–Cl)	2.55 (s, CH₃), 2.73 (s, CH₂), 2.89 (s, OCH₃), 7.68–7.72 (d, 2H), 9.77 (s, OH)	31.25, 34.86, 36.26, 108.59, 109.75, 115.34, 126.54, 129.70, 130.88, 133.08, 135.79, 136.79, 142.20, 144.90, 147.95, 149.70, 153.49, 159.34, 161.79, 162.83, 166.22, 195.50	521
6c	3088 Ar–CH), 2960 (aliphatic CH), 2240 (C≡N), 1692, 640 (C–Cl)	2.73 (s, CH₃), 2.89 (s, CH₂), 6.62–6.66 (d, 2H), 8.65 (s, CH _Triazolo_)	–	544

### Density functional theory study

Density functional theory (DFT) is used to assess the relative stability of the formed stereoisomers of the compound and study their electronic properties. First, geometry optimization was performed to get the structure with minimum energy, and a solvent model was used in this process to mimic the biological fluid, which is composed mostly of water. Then the frequency analysis was performed to make sure that there is no negative frequency obtained, which confirms reaching a real minimum and not a stationary point. The thermodynamic Gibbs free energy was calculated within the frequency calculation and used to estimate the relative stability of different stereoisomers for the synthesized compounds.

**Fig. 10:**
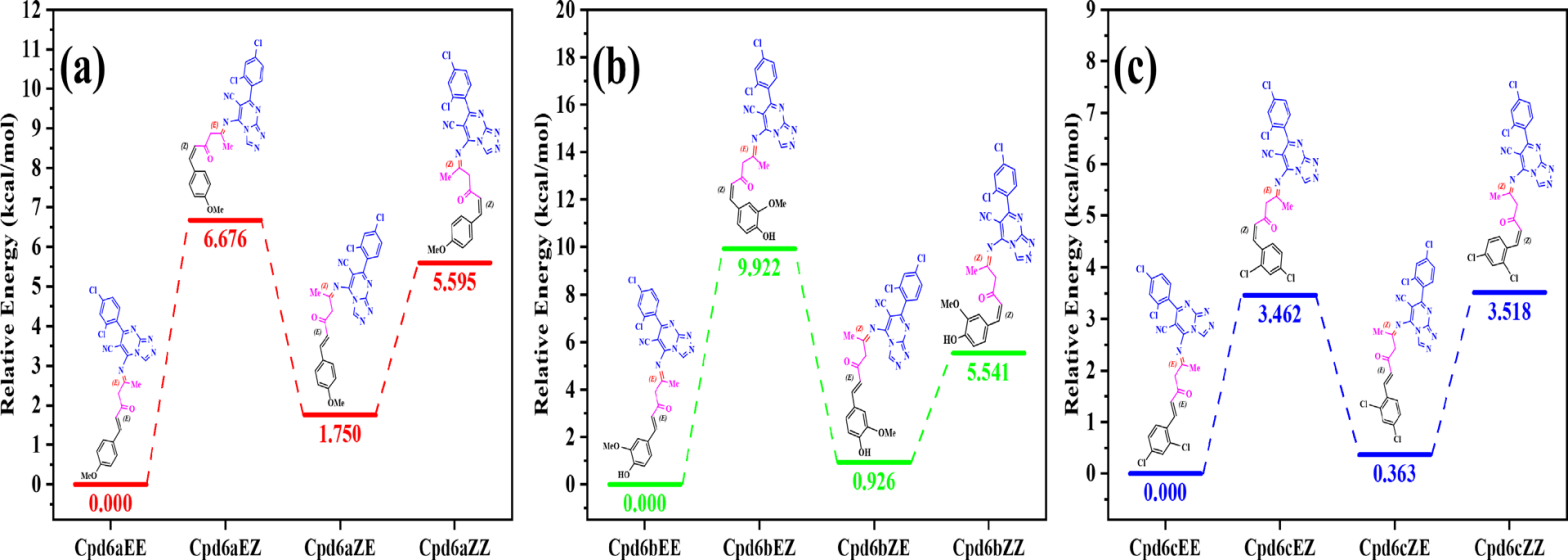
Calculated relative energies of the stereoisomeric forms of Compound 6a (**a**), Compound 6b (**b**), and Compound 6c (**c**) obtained using density functional theory (DFT) at the B3LYP/RIJCOSX/6-31G(d,p) level of theory with the CPCM solvation model. The energy differences between stereoisomers are presented to evaluate their relative thermodynamic stability in the solvent phase. Lower relative energy values indicate more stable conformations, providing insight into the preferred stereochemical configuration of each compound under the studied conditions.

From the proposed reaction mechanism, several stereoisomers for a synthesized compound could be obtained. The E and Z isomers could be obtained for **Compound 5a**, **Compound 5b,** and **Compound 5c**. For **Compound 6a**, **Compound 6b**, and **Compound 6c**, four possible stereoisomers are possible to be produced, namely**, EE, EZ, ZE, and ZZ.** The structures of these stereoisomers are depicted in Fig. S1. For compounds that possess E and Z isomers, it is predicted that the E isomer will constitute the highest fraction of the reaction yield since it is thermodynamically more stable^39,40^. The relative energies obtained from the DFT calculations for the stereoisomers of **Compound 6a**, **Compound 6b**, and **Compound 6c** are shown in Fig. 10a–c, respectively. These relative energies were calculated with respect to the most stable isomer. For **Compound 6a**, it can be noticed that the stereoisomers **Compound 6aEE** (the most stable isomer) and **Compound 6aZE** (with relative energy of 1.750 kcal/mol) show the lower energies compared to **Compound 6aEZ** (with relative energy of 6.676 kcal/mol) and **Compound 6aZZ** (with relative energy of 5.595 kcal/mol). For **Compound 6b**, the stereoisomers that exhibit lower relative energies are **Compound 6bEE** (the most stable isomer) and **Compound 6bZE** (with relative energy of 0.926 kcal/mol), compared to **Compound 6bEZ** (with relative energy of 9.922 kcal/mol) and **Compound 6bZZ** (with a relative energy of 5.541 kcal/mol). For the third compound, **Compound 6c**, the lower relative energy isomers are **Compound 6cEE** (the most stable isomer) and **Compound 6cZE** (with a relative energy of 0.363 kcal/mol) compared to **Compound 6cEZ** (with a relative energy of 3.462 kcal/mol) and **Compound 6cZZ** (with a relative energy of 3.518 kcal/mol). From these results, it is predicted that the stereoisomers **EE** and **ZE** shall constitute the highest portion of the yield for **Compound 6a**, **Compound 6b**, and **Compound 6c**.

**Fig. 11:**
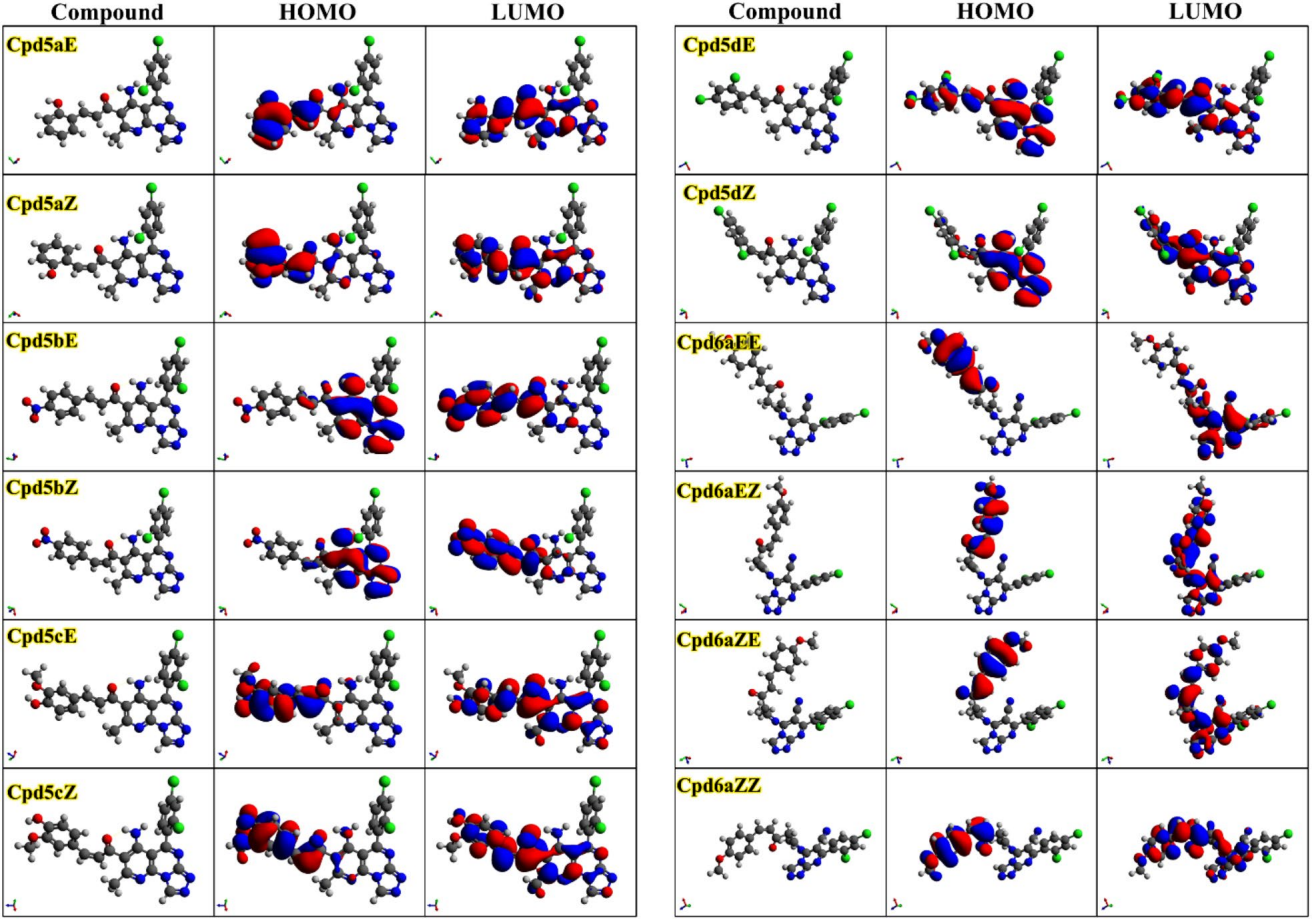

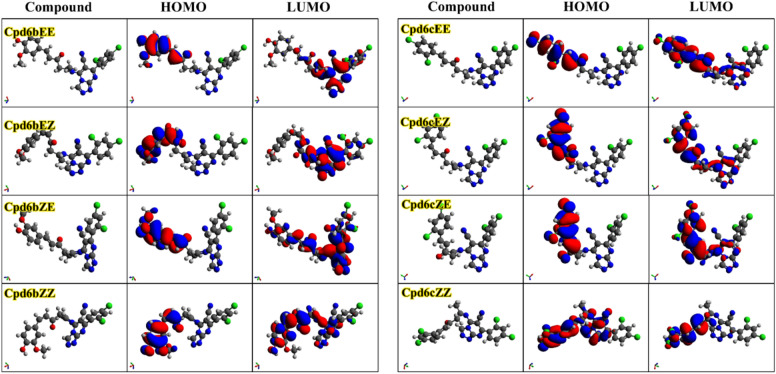
Geometry-optimized structures of the investigated compounds obtained using density functional theory (DFT) at the B3LYP/RIJCOSX/6-31G(d,p) level of theory with the CPCM solvation model. The optimized geometries represent the lowest-energy conformations in the solvent phase. The corresponding frontier molecular orbitals, including the highest occupied molecular orbital (HOMO) and lowest unoccupied molecular orbital (LUMO), are also illustrated to provide insight into the electronic distribution, charge-transfer characteristics, and reactivity profiles of the studied compounds. The geometry optimized structures of the investigated compounds using B3LYP/RIJCOSX/6-31G(d,p) and the solvent model CPCM, along with the obtained frontier molecular orbitals.

The Frontier Molecular Orbital (FMO) of the isomers of the synthesized compounds is presented in Fig. 11. They are used to assess their electronic properties. A noticeable spatial separation between the HOMO and LUMO regions is observed in several stereoisomers, most notably within the **Compound 5b**, **Compound 6a**, and **Compound 10b** stereoisomers.

**Fig. 12:**
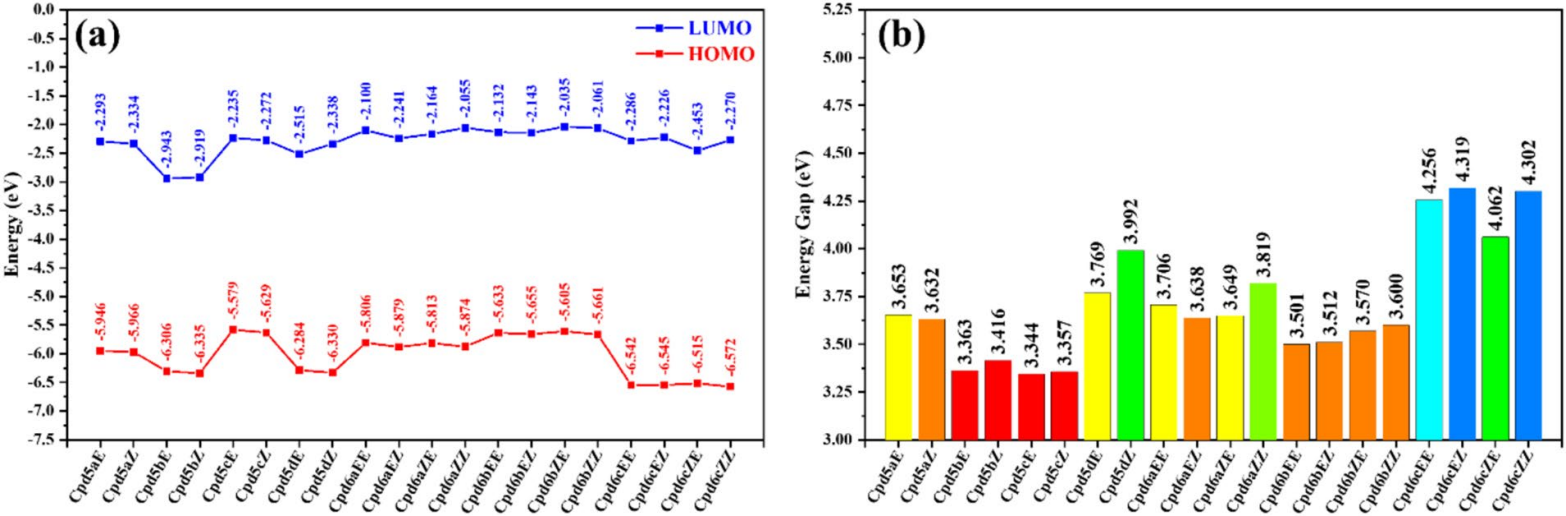
The HOMO and LUMO energies of the investigated structures (**a**) and the calculated energy gap (**b**).

The HOMO and LUMO energies of the compounds are presented in Fig. 12a and its corresponding energy gaps in Fig. 12b. The higher the HOMO energy, the greater the molecule’s ability to donate electrons. On the other hand, the lower the LUMO energy, the greater the molecule’s ability to accept electrons^41^. It can be noticed that the compound with the highest HOMO energy is **Compound 5cE,** with an energy of − 5.579 eV. Conversely, the one that shows the lowest HOMO energy is **Compound 6cZZ** with an energy of − 6.572 eV. When it comes to LUMO energies, the isomer that has higher LUMO energy is **Compound 6bZE** with an energy of − 2.035 eV. The stereoisomer that shows the lowest LUMO energy is **Compound 5bE** with an energy of − 2.943 eV.

The energy gap of a molecule is used to estimate its chemical reactivity. The higher energy gap indicates a more stable molecule, while the lower energy gap indicates a more reactive molecule and more feasible electron transference between two molecules, consequently enhancing the possibility of interactions with biological molecular targets^41^. When the energy gaps are considered, it was found that the compound that shows the highest energy gaps for its stereoisomers is **Compound 6c** with energies of 4.256 eV, 4.319 eV, 4.062 eV, and 4.302 eV for **EE, EZ, ZE,** and **ZZ,** respectively. This indicates that **Compound 6c** with its possible stereoisomers is predicted to be the most chemically inert out of the synthesized compounds. When it comes to the compounds that shows a lower energy gap, it can be noticed that isomers of **Compound 5b** and **Compound 5c** have the lower values. They are 3.344 eV, 3.357 eV, 3.363 eV, and 3.416 eV for **Compound 5bE**, **Compound 5bZ**, **Compound 5cE**, and **Compound 5cZ**, respectively. These results indicate that both **Compound 5b** and **Compound 5c** will exhibit higher chemical reactivity. Other reactivity descriptors, namely.

- Ionization potential (IP) = − E_HOMO_
- Electron affinity (Ea) = − E_LUMO_
- Electronic chemical potential (µ) = (E_HOMO_ + E_LUMO_)/2
- Chemical hardness (η) = (E_LUMO_ − E_HOMO_)/2
- Chemical softness (S) = 1/η
- Electrophilicity index (ω) = (µ)^2^/(2η)
- Maximum charge acceptance = − µ/η
- Nucleophilicity index (N) = E_HOMO_ (Compound) − E_HOMO_ (TCE), relative to tetracyanoethylene, which is expected to be the least nucleophilic neutral species^42^.

Were calculated and presented in Table S1.

### Computational analysis

#### Molecular docking of selected molecules with the IL-6 (PDB ID: 1N26) receptor

**Fig. 13.**
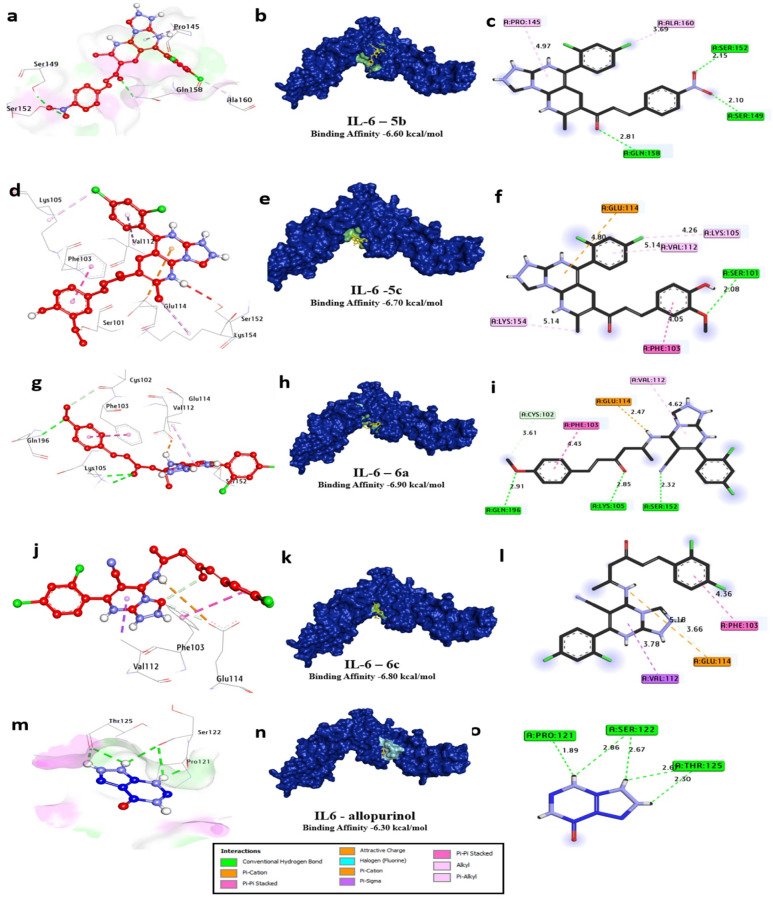
3D representations of compound conformations at the binding pockets of the IL-6 (PDB ID: 1N26) protein: (**a**–**c**) 5b, (**d**–**f**) 5c, (**g**–**i**) 6a, (**j**–**l**) 6c (**m**–**o**) allopurinol as a standard drug.

In gout, interleukin-6 (IL-6) functions as a critical pro-inflammatory cytokine that amplifies and sustains the severe inflammatory response to monosodium urate (MSU) crystals in the joint. It is not the initial trigger but a major driver of the systemic and local symptoms. Based on the provided Table 6 for molecular interactions of ligands with the interleukin-6 (IL-6) protein, here is the detailed analysis presented as a cohesive paragraph. The molecular docking analysis of four ligand candidates **(5b, 5c, 6a, 6c)** against the IL-6 protein reveals their potential to disrupt the cytokine’s pro-inflammatory signaling, which is pivotal in gout pathogenesis. All four test ligands (5b, 5c, 6a and 6c) exhibited moderate to good, predicted binding affinities against IL-6 (PDB ID: 1N26), ranging from − 6.60 to − 6.90 kcal/mol, all of which are more favorable than that of the reference drug allopurinol (− 6.30 kcal/mol). Notably, the binding poses cluster in two key functional regions of IL-6 known as site II and site III, which are critical for engaging the gp130 signal-transducing subunit. Ligands **5b** and **6a** employ a strategy involving multiple hydrogen bonds (three each) with polar residues like Ser149, Ser152, Gln158, and Gln196, which likely anchor them precisely within the binding pocket. In contrast, Ligand **5c** forms only a single strong hydrogen bond with Ser101 but compensates with a more extensive set of hydrophobic and electrostatic interactions, including a favorable pi-pi stacked interaction with Phe103 and a pi-cation interaction with Glu114. Ligand **6c** is unique, forming no conventional hydrogen bonds and relying entirely on hydrophobic and weak polar contacts, such as pi-sigma and carbon-hydrogen bonds, yet still achieves a significant binding affinity. A common and critical interaction observed for three of the four ligands **(5c, 6a, 6c)** is the pi-cation interaction with Glu114, underscoring the importance of this negatively charged residue for ligand stabilization. From a therapeutic standpoint for gout, these ligands, particularly **6a**, represent promising starting points. By binding to IL-6's gp130 interaction sites, they could sterically hinder the formation of the active IL-6/IL-6R/gp130 signaling hexamer, thereby inhibiting the downstream JAK/STAT3 pathway responsible for amplifying inflammation, acute-phase protein production, and fever during a gout flare. The combination of hydrogen-bonding precision and strategic hydrophobic contacts makes **6a** a compelling lead for further optimization toward a novel oral IL-6 signaling inhibitor. Overall, the novel ligands **5c**, **6a** and **6c** demonstrate competitive affinities, relying on conserved hydrophobic residues Glu114 and Phe103 in the IL-6 signaling inhibitor. These in silico findings are consistent with recent studies^43^, which also used a network pharmacology approach, molecular docking, and experimental validation to investigate the pharmacological mechanisms of baicalin in treating gout (Table 6 and Fig. 13). By comparison, allopurinol formed five hydrogen bonds but lacked significant hydrophobic contacts, consistent with its lower affinity. While the predicted binding energies are only modestly improved over the standard drug, the distinct interaction patterns, particularly the combination of hydrogen bonding with hydrophobic packing, suggest that 6a and related compounds may serve as promising starting points for further optimization. However, these in silico results require experimental validation (e.g., surface plasmon resonance or enzymatic inhibition assays) to confirm actual inhibitory potency.

**Table 6:** Molecular interactions of ligands with the IL-6 (PDB ID: 1N26) protein.

No.	Protein	Ligand	3D structure	Hydrophilic interactions		Hydrophobic contacts		No. of H-bonds	No. of total bonds	Affinity kcal mol-1
				Residue (H-Bond)	Length (Å)	Residue (bond type)	Length (Å)			
1	IL-6 (PDB.ID: 1N26)	5b		Gln158, (H-Bond) Ser149, (H-Bond) Ser152, (H-Bond)	2.81 2.10 2.15	Ala160, (Pi-alkyl) Pro145, (Pi-alkyl)	3.69 4.97	**3**	**5**	**− 6.60**
2		5c		Ser101, (H-Bond)	2.08	Lys105, (Pi-alkyl) Val112, (Pi-alkyl) Lys154, (Pi-alkyl) Phe103, (Pi-pi Stacked) Glu114, (Pi-cation)	4.26 5.14 5.14 4.05 4.80	**1**	**6**	**− 6.70**
3		6a		Gln196, (H-Bond) Lys105, (H-Bond) Ser152, (H-Bond)	2.91 2.85 2.32	Val112, (Pi-alkyl) Phe103, (Pi-pi Stacked) Glu114, (Pi-cation) Cys102, (Carbon H bond)	4.62 4.43 2.47 3.61	**3**	**7**	**− 6.90**
4		6c		–	–	Val112, (Pi-sigma) Phe103, (Pi-pi Stacked) Glu114, (Pi-cation) Glu114, (Carbon H bond)	3.78 5.18 4.36 3.66	**0**	**4**	**− 6.80**
5		allopurinol (drug)		Pro121, (H-Bond) Ser122, (H-Bond) Ser122, (H-Bond) Thr125, (H-Bond) Thr125, (H-Bond)	1.89 2.86 2.67 2.61 2.30	–	–	**5**	**5**	**− 6.30**

#### Molecular docking of selected molecules with the TNF (PDB ID: 5MU8) receptor

**Fig.14.**
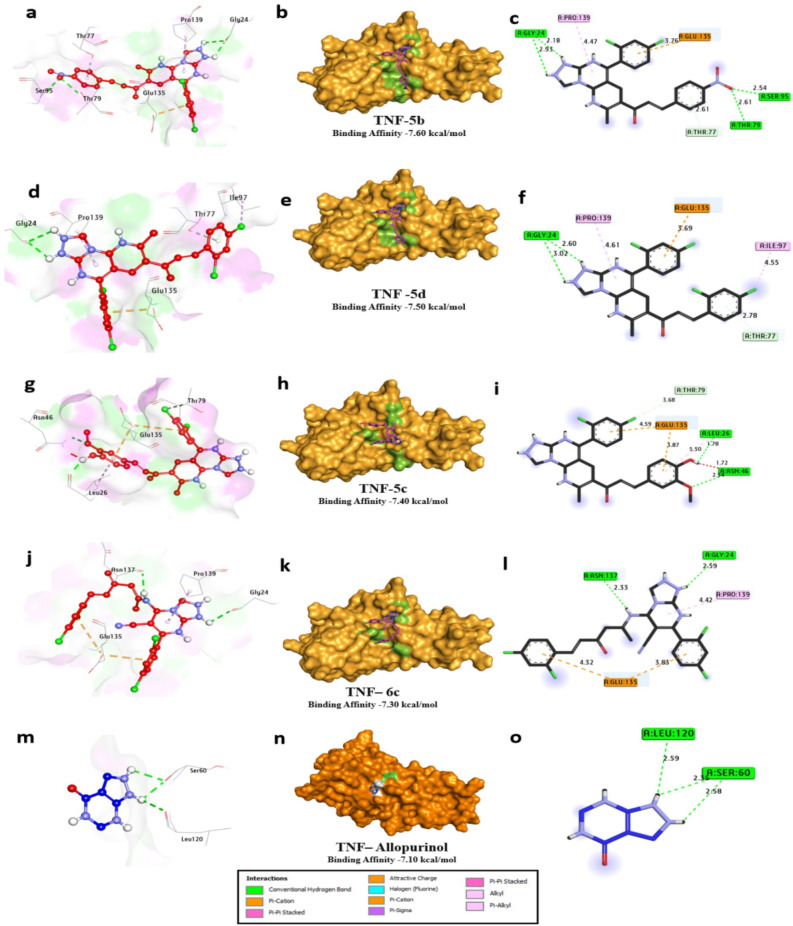
3D representations of compound conformations at the binding pockets of the TNF (PDB ID: 5MU8) protein: (**a–c**) 5b, (**d–f**) 5d, (**g–i**) 5c, (**j–l**) 6c, and (**m–o**) allopurinol as a standard drug.

Based on the provided a detailed analysis of the molecular docking results for ligands binding to the tumor necrosis factor-alpha (TNF-α) protein (PDB ID: 5MU8). This docking study identifies several promising small-molecule ligands capable of binding to the TNF-α trimer with high predicted affinity. Table 7 summarizes the docking results for TNF-α (PDB ID: 5MU8). All test ligands (5b, 5d, 5c, 6c) showed predicted binding affinities between − 7.30 and − 7.60 kcal/mol, consistently outperforming allopurinol (− 7.10 kcal/mol). Notably, all four ligands share a highly consistent interaction profile, targeting a specific pocket within the TNF-α subunit that is critical for its function. This pocket is characterized by key residues Gly24, Thr77, Leu26, and Glu135. The recurrent binding to this site suggests it is a hot spot for inhibiting TNF-α activity, likely by preventing the proper subunit assembly or interaction with its receptor (TNFR1). Ligand **5b** achieves its top affinity through an optimal combination of four hydrogen bonds (Ser95, Thr79, and two with Gly24) and three stabilizing hydrophobic/electrostatic contacts, including a Pi-cation interaction with Glu135. The other ligands **(5d, 5c, 6c)** employ similar strategies but with fewer hydrogen bonds, relying more on crucial Pi-cation interactions with Glu135 and Pi-alkyl contacts with Pro139 for stabilization. From a therapeutic perspective for gout, these results are significant. TNF-α is a potent pro-inflammatory cytokine that amplifies joint inflammation and tissue damage during a flare. A small molecule that effectively blocks this functional site on TNF-α could potently suppress the inflammatory cascade, working synergistically with or as an alternative to current biologic anti-TNF therapies. The high affinity and consistent binding mode of Ligand **5b**, in particular, mark it as a compelling lead candidate for further development as a novel oral TNF inhibitor. These findings suggest that the compounds **5d, 5c, and 6c** have potential as TNF-α activity inhibitors. These in-silico findings are consistent with recent studies^44^, which also report potent TNF-α activity inhibition, underscoring the potential of these compounds as TNF-α inhibitors (Table 7 and Fig. 14). The docking protocol was first validated by redocking the native co-crystallized ligand into each target binding site. Low RMSD values (≤ 1.65 Å for all complexes) confirmed that the docking parameters reliably reproduce the experimental binding modes, enabling confident interpretation of the test ligand poses.

**Table 7:** Molecular interactions of ligands with TNF (PDB.ID: 5MU8) protein.

No.	Protein	Ligand	3D structure	Hydrophilic interactions		Hydrophobic contacts		No. of H-bonds	No. of total bonds	Affinity kcal mol-1
				Residue (H-Bond)	Length (Å)	Residue (bond type)	Length (Å)			
1	TNF (PDB.ID: 5MU8)	5b		Ser95, (H-Bond) Thr79, (H-Bond) Gly24, (H-Bond) Gly24, (H-Bond)	2.54 2.61 2.18 2.93	Pro139, (Pi-alkyl) Glu135, (Pi-cation) Thr77, (Carbon H nond)	4.47 3.76 2.61	**4**	**7**	**− 7.60**
2		5d		Gly24, (H-Bond) Gly24, (H-Bond)	2.60 3.02	Pro139, (Pi-alkyl) Ile97, (Pi-alkyl) Glu135, (Pi-cation) Thr77, (Carbon H nond)	4.47 4.55 3.76 2.61	**2**	**6**	**− 7.50**
3		5c		Leu26, (H-Bond) Asn46, (H-Bond)	1.78 2.54	Glu135, (Pi-cation) Glu135, (Pi-cation) Leu26, (Pi-alkyl) Thr79, (Carbon H nond)	4.59 3.87 5.50 3.68	**2**	**6**	**− 7.40**
4		6c		Gly24, (H-Bond) Asn137, (H-Bond)	2.59 2.33	Pro139, (Pi-alkyl) Glu135, (Pi-cation) Glu135, (Pi-cation)	4.42 3.83 4.32	**2**	**5**	**− 7.30**
5		Allopurinol (drug)		Leu120, (H-Bond) Ser60, (H-Bond) Ser60, (H-Bond)	2.59 2.35 2.58	–	–	**3**	**3**	**− 7.10**

#### Molecular docking of selected molecules with the MMP9 (PDB ID: 6ESM) receptor

**Fig. 15.**
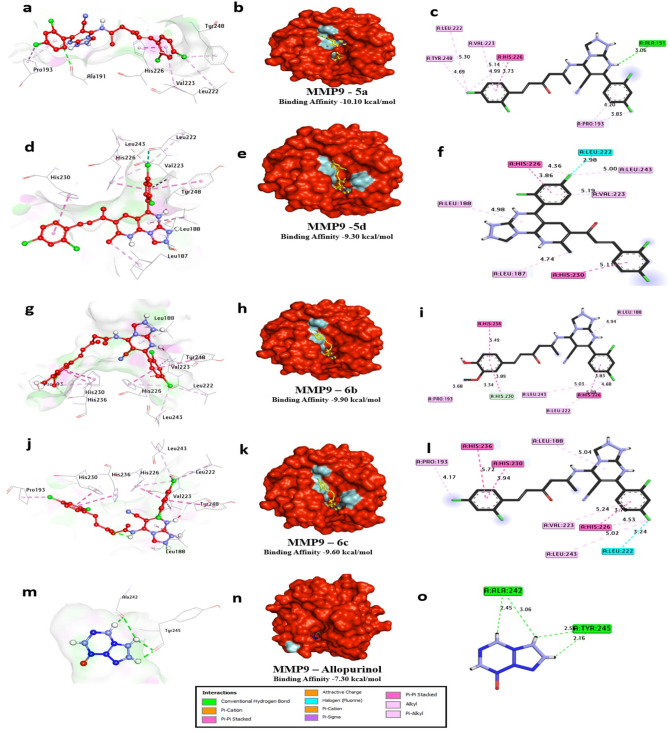
3D representations of compound conformations at the binding pockets of the MMP (PDB ID: 6ESM) protein: (**a–c**) 5a, (**d–f**) 5d, (**g–i**) 6b, (**j–l**) 6c, and (**m–o**) allopurinol as a standard drug.

As presented in Table 8, all test ligands bound to MMP-9 (PDB ID: 6ESM) with markedly higher affinities (− 8.00 to − 10.10 kcal/mol) than allopurinol (− 7.30 kcal/mol). Ligand 5a demonstrated the strongest predicted binding (− 10.10 kcal/mol), which is 2.8 kcal/mol more favorable than the control. This docking study identifies several high-affinity ligands targeting the Matrix Metalloproteinase-9 (MMP-9) catalytic domain, a key enzyme involved in tissue remodelling and inflammatory joint destruction in diseases like gout. Among the tested compounds, Ligand **5a** stands out as the most potent candidate with a significant predicted binding affinity of − 10.10 kcal mol^−1^, significantly higher than **5d** (− 9.30), 6c (− 8.70), and 6b (− 8.00). This superior affinity is structurally rationalized by its unique ability to form a direct hydrogen bond with the backbone of Ala191 (2.23 Å) within the enzyme’s active site, a polar interaction absent in the other ligands. This crucial H-bond likely serves as a strong anchoring point, complementing its extensive hydrophobic network. All four ligands share a highly consistent binding mode, deeply embedded within the hydrophobic S1’ subsite (or specificity pocket) of MMP-9, as evidenced by their universal and numerous contacts with residues Leu188, Pro193, Leu222, Val223, His226, and Leu243. Ligands **5d**, **6b**, and **6c** achieve their stabilization exclusively through an array of 8 hydrophobic and pi-interactions per ligand, including multiple pi-alkyl and pi-pi Stacked interactions with key histidine residues (His226, His230, and His236) that line the pocket. Notably, ligands **5d** and **6c** also exhibit halogen bonds with Leu222, a favorable interaction that contributes to their specificity and binding strength. From a therapeutic perspective for gout, these results are highly promising. MMP-9 is upregulated in inflamed synovial tissue and contributes to cartilage degradation and leukocyte migration during a flare. A potent, small-molecule inhibitor, **5a** which combines a strategic hydrogen bond with optimal hydrophobic complementarity in the deep S1 pocket, could effectively block MMP-9 proteolytic activity, thereby protect joint integrity and modulate the inflammatory cascade. This positions **5a** as a prime lead compound for the development of novel disease-modifying agents in gout and other MMP-9-driven pathologies. These in silico findings are consistent with recent studies^45^, which also report potent MMP-9 inhibition, underscoring the potential of identified compounds as Antidiabetic agents (Table 8 and Fig. 15).

**Table 8:** Molecular interactions of ligands with the MMP9 (PDB ID: 6ESM) protein.

No.	Protein	Ligand	3D structure	Hydrophilic interactions		Hydrophobic contacts		No. of H-bonds	No. of total bonds	Affinity kcal mol-1
				Residue (H-bond)	Length (Å)	Residue (bond type)	Length (Å)			
1	MMP9 (PDB.ID: 6ESM)	5a		Ala191, (H-bond)	2.23	Leu222, (Pi-alkyl) Tyr248, (Pi-alkyl) His226, (Pi-alkyl) Val223, (Pi-alkyl) Pro193, (Pi-alkyl) Pro193, (Pi-alkyl) Phe9, (Pi-pi Stacked)	5.30 4.69 4.99 5.14 4.20 3.83 3.73	**1**	**8**	**− 10.10**
2		5d		–	–	Leu243, (Pi-alkyl) Val223, (Pi-alkyl) His226, (Pi-alkyl) Leu188, (Pi-alkyl) Leu187, (Pi-alkyl) Leu222, (Halogen) His230, (Pi-pi Stacked) His226, (Pi-pi Stacked)	5.00 5.19 4.36 4.98 4.74 2.98 5.11 3.86	**0**	**8**	**− 9.30**
3		6b		–	–	Pro193, (Pi-alkyl) Leu243, (Pi-alkyl) Leu222, (Pi-alkyl) Leu188, (Pi-alkyl) His230, (Carbon H bond) His230, (Pi-pi Stacked) His236, (Pi-pi Stacked) His226, (Pi-pi Stacked)	3.68 5.03 5.09 4.94 3.34 3.89 5.49 3.83	**0**	**8**	**− 8.00**
4		6c		–	–	Leu243, (Pi-alkyl) Val223, (Pi-alkyl) Pro193, (Pi-alkyl) Leu188, (Pi-alkyl) Leu222, (Halogen) His230, (Pi-pi Stacked) His226, (Pi-pi Stacked) His236, (Pi-pi Stacked)	5.02 5.24 4.17 5.04 3.24 3.94 3.79 5.72	**0**	**8**	**− 8.70**
5		Allopurinol (drug)		Tyr245, (H- Bond) Tyr245, (H- Bond) Ala242, (H- Bond) Ala242, (H- Bond)	2.50 2.16 3.06 2.45	–	–	**4**	**4**	**− 7.30**

#### Molecular docking of selected molecules with the NLRP3 (PDBID: 7ALV) receptor

**Fig. 16.**
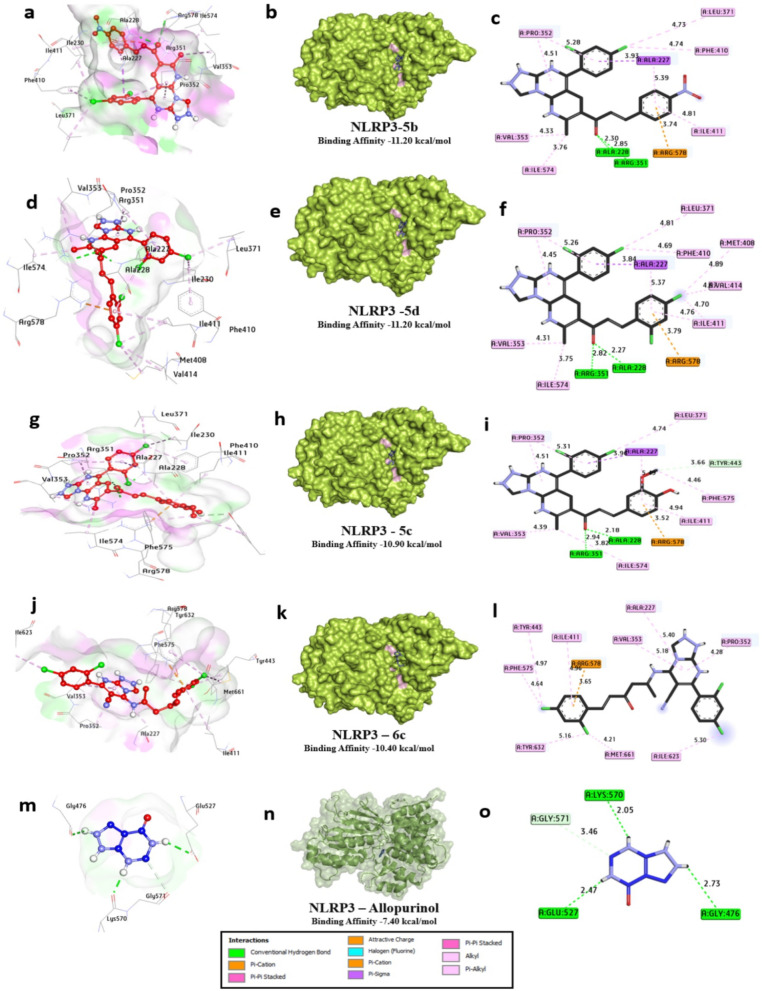
3D representations of compound conformations at the binding pockets of the NLRP3 (PDB ID: 7ALV) protein: (**a–c**) 5b, (**d–f**) 5d, (**g–i**) 5c, (**j–l**) 6c, and (**m–o**) allopurinol as a standard drug.

This docking study reveals strong binding of several small-molecule ligands to the NLRP3 inflammasome protein, a central driver of the inflammatory cascade in gout. Table 9 reveals strong binding of test ligands to NLRP3 (PDB ID: 7ALV), with predicted affinities ranging from − 10.40 to − 11.20 kcal/mol. These values substantially exceed that of allopurinol (− 7.40 kcal/mol), representing an improvement of 3.0–3.8 kcal/mol. The results demonstrate extraordinarily high predicted binding affinities, with **5b** and **5d** sharing the top affinity of − 11.20 kcal mol^−1^, followed by **5c** (− 10.90) and 6c (− 10.40), compared to allopurinol (as a standard drug) (− 7.40). This indicates a very potent and spontaneous binding interaction, which is structurally explained by the ligands occupying a deep, hydrophobic pocket within the NLRP3 NACHT domain, a region critical for its ATPase activity and oligomerization. The binding mode is highly consistent across the top three ligands **(5b, 5d, 5c)**, characterized by a dual hydrogen-bonding anchor formed with Ala228 (2.30 Å) and Arg351 (2.85 Å). This polar interaction network provides precise orientation and specificity, locking the ligands in place within the active site. The immense binding strength is further amplified by an extensive hydrophobic cage formed by residues Leu371, Pro352, Ile411, Val353, Ile574, and Phe410. Ligands **5b, 5d,** and **5c** form between 12 and 14 total bonds, dominated by numerous Pi-alkyl interactions and a key stabilizing Pi-cation interaction with Arg578. Ligand **6c**, while still highly potent, adopts a slightly different strategy, forming no conventional hydrogen bonds but achieving significant affinity (− 10.40 kcal mol^−1^) through a dense network of 10 hydrophobic contacts, including the conserved Pi-cation interaction with Arg578. For gout pathophysiology, these results are of paramount importance. The NLRP3 inflammasome is the direct sensor for monosodium urate (MSU) crystals and is responsible for activating caspase-1 and triggering the maturation of IL-1β, the master cytokine of an acute gout flare. A small-molecule inhibitor that binds with such high affinity to this specific pocket, as demonstrated by **5b** and **5d**, could directly and potently inhibit NLRP3 activation, thereby blocking the production of IL-1β at its source. This represents a potentially superior therapeutic strategy, targeting the inflammasome upstream of cytokine release, and identifies these ligands as outstanding lead compounds for the development of novel, orally bioavailable anti-inflammatory drugs for gout and other NLRP3-mediated diseases (Table 9 and Fig. 16).

**Table 9:** Molecular interactions of ligands with the NLRP3 (PDB ID: 7ALV) protein.

No.	Protein	Ligand	3D structure	Hydrophilic interactions		Hydrophobic contacts		No. of H-bonds	No. of total bonds	Affinity kcal mol-1
				Residue (H-bond)	Length (Å)	Residue (bond type)	Length (Å)			
1	NLRP3 (PDB.ID: 7ALV)	5b		Ala228, (H-Bond) Arg351, (H-Bond)	2.30 2.85	Leu371, (Pi-alkyl) Phe410, (Pi-alkyl) Pro352, (Pi-alkyl) Pro352, (Pi-alkyl) Ala227, (Pi-alkyl) Ile411, (Pi-alkyl) Val353, (Pi-alkyl) Ile574, (Pi-alkyl) Ala227, (Pi-sigma) Arg578, (Pi-cation)	4.73 4.74 5.28 4.51 5.39 4.81 4.33 3.76 5.39 3.74	**2**	**12**	**− 11.20**
2		5d		Ala228, (H-Bond) Arg351, (H-Bond)	2.30 2.85	Leu371, (Pi-alkyl) Phe410, (Pi-alkyl) Pro352, (Pi-alkyl) Pro352, (Pi-alkyl) Ala227, (Pi-alkyl) Ile411, (Pi-alkyl) Val353, (Pi-alkyl) Ile574, (Pi-alkyl) Met408, (Pi-alkyl) Phe410, (Pi-alkyl) Ala227, (Pi-sigma) Arg578, (Pi-cation)	4.81 4.69 5.26 4.45 5.37 4.76 4.31 3.75 4.89 4.69 3.84 3.79	**2**	**14**	**− 11.20**
3		5c		Ala228, (H-Bond) Arg351, (H-Bond)	2.30 2.85	Leu371, (Pi-alkyl) Pro352, (Pi-alkyl) Pro352, (Pi-alkyl) Phe575, (Pi-alkyl) Ile411, (Pi-alkyl) Val353, (Pi-alkyl) Ile574, (Pi-alkyl) Ala227, (Pi-alkyl) Tyr443, (Carbon H bond) Ala227, (Pi-sigma) Arg578, (Pi-cation)	4.74 5.31 4.51 4.46 4.94 4.39 3.82 5.45 3.66 3.96 3.52	**2**	**13**	**− 10.90**
4		6c		–	–	Ala227, (Pi-alkyl) Pro352, (Pi-alkyl) Val353, (Pi-alkyl) Ile411, (Pi-alkyl) Tyr443, (Pi-alkyl) Phe575, (Pi-alkyl) Tyr632, (Pi-alkyl) Met661, (Pi-alkyl) Ile623, (Pi-alkyl) Arg578, (Pi-cation)	5.40 4.28 5.18 4.96 4.97 4.64 5.16 4.21 5.30 3.65	**0**	**10**	**− 10.40**
5		Allopurinol (drug)		Gly476, (H-Bond) Lys570, (H-Bond) Glu527, (H-Bond)	2.73 2.05 2.47	Gly571, (Carbon H bond)	3.46	**3**	**4**	**− 7.40**

### Correlation of DFT descriptors with docking affinities

The integrated experimental and computational evaluation of triazolopyrimidine–chalcone hybrids (compounds 5a–d and 6a–c) against the gout‑related targets IL‑6, TNF‑α, MMP‑9, and NLRP3 has enabled the delineation of a comprehensive structure–activity relationship (SAR). All tested compounds share a rigid, π‑rich triazolo[4,3‑a]pyrimidine core, which serves as a privileged scaffold providing essential π‑stacking ability and hydrogen‑bond acceptor capacity. This core alone, however, is not sufficient for high affinity; its activity is strongly modulated by the substituents at the C‑2 position and by the attached chalcone moiety. Two main sub‑series were investigated: series 5a–d, which retain an NH₂ group at C‑2, and series 6a–c, which contain a nitrile (C≡N) group together with a methylene linker. The NH₂ group in series 5 acts as an additional hydrogen‑bond donor, favoring binding to the cytokine targets IL‑6 and TNF‑α by forming polar contacts with residues such as Ser149/152 (IL‑6) and Asn46 (TNF‑α). In contrast, the nitrile group in series 6 is a strong hydrogen‑bond acceptor but lacks a donor; together with the flexible methylene linker, it enhances binding to the deep, hydrophobic pockets of MMP‑9 and NLRP3, as exemplified by compound 6c and the high affinity of 5a for MMP‑9.

Density functional theory (DFT) calculations of frontier molecular orbitals provide electronic justification for the observed SAR. Compounds with low HOMO–LUMO gaps (high chemical reactivity), notably 5b and 5c (ΔE ≈ 3.34–3.42 eV), exhibit the strongest binding to IL‑6 and TNF‑α, consistent with their greater polarizability and ability to engage in Pi‑cation interactions (e.g., with Arg578 in NLRP3 or Glu135 in TNF‑α). In contrast, the more chemically inert 6c (ΔE ≈ 4.3 eV) still binds effectively to NLRP3, suggesting that deep hydrophobic pockets do not require high electronic reactivity. Global electrophilicity and chemical softness further correlate with multi‑target promiscuity; the softest molecules (5b) are the most balanced across all four targets.

#### In silico pharmacokinetics ADMET prediction of synthesized compounds

**Fig. 17:**
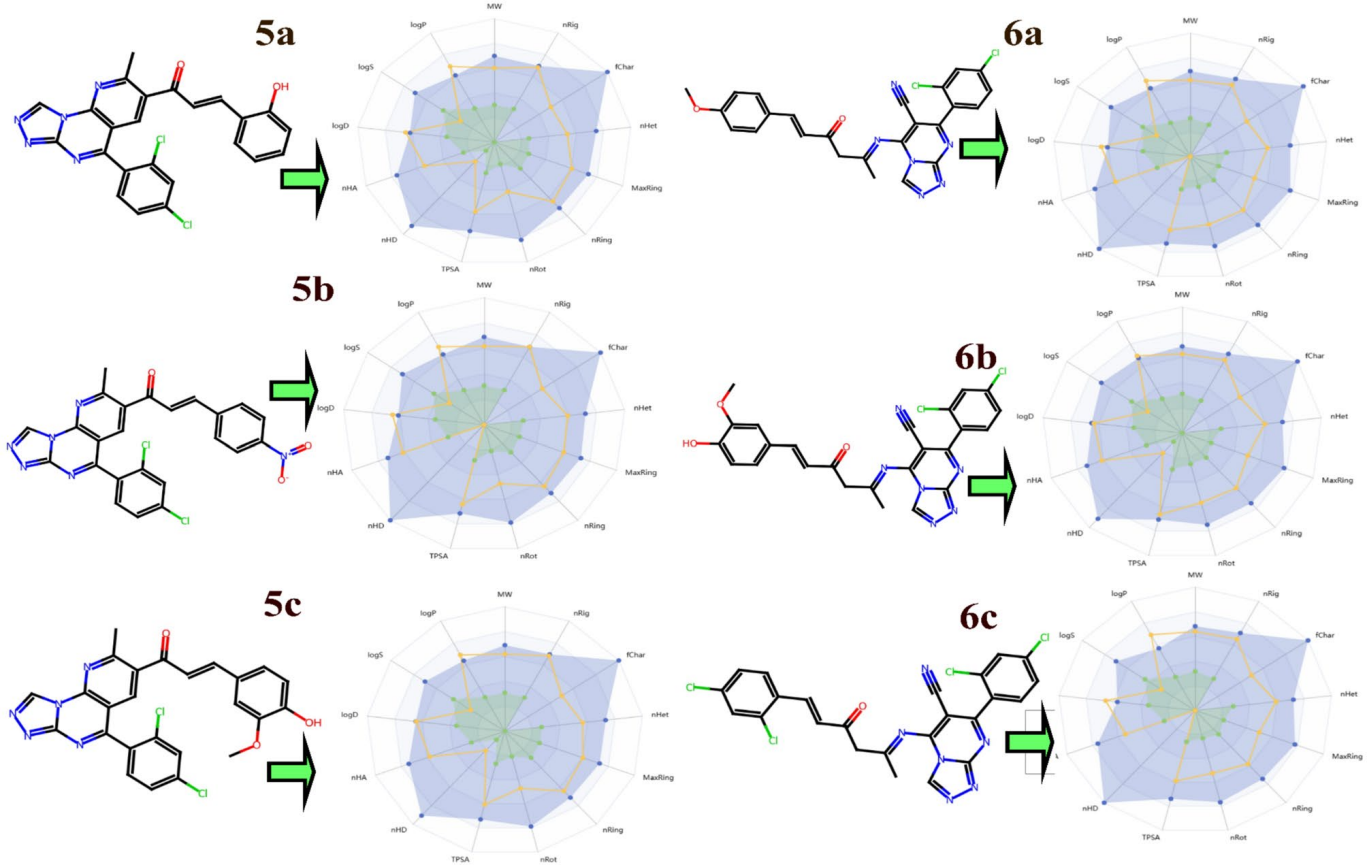
Oral bioavailability analysis of compounds using ADMETlab 3.0. (**A**) depicts the chemical structure of compounds 5a–d and 6a–c. (**B**) Shows there bioavailability radar plot, graphically summarizing key physicochemical properties governing absorption.

**Fig. 18:**
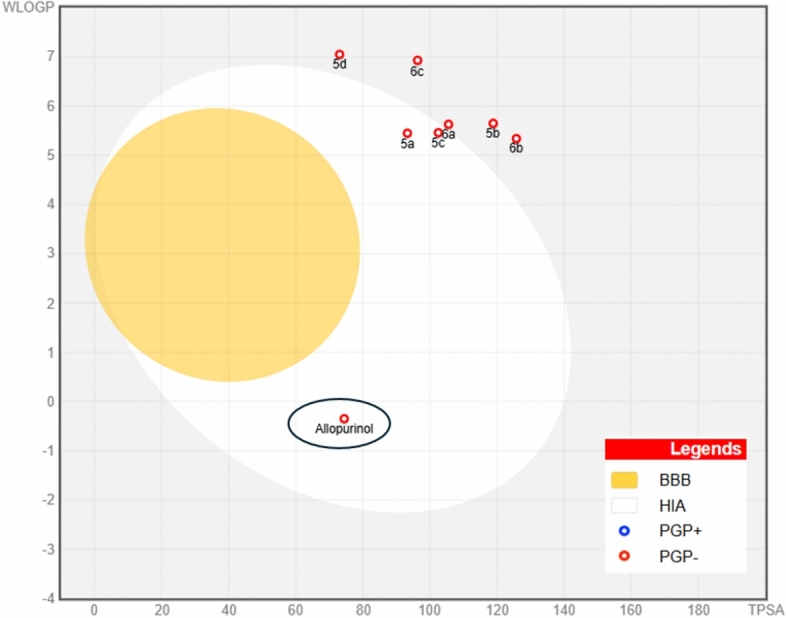
Boiled egg bioavailability analysis of compounds using SwissADME depicts compounds 5a–d and 6a–c compared with the reference drug (allopurinol).

ADMET profiling (Tables 10, 11, Figs. 17, 18), Fig. 2S. The absorption, distribution, metabolism, excretion, and toxicity (ADMET) properties of all test compounds **(5a–d, 6a–c)** were predicted using ADMET lab 3.0 and Swiss ADME, with allopurinol as a reference drug. The results reveal several significant pharmacokinetic and toxicity liabilities that temper the initial promise from docking and MD simulations. Physicochemical and solubility issues. All test compounds exhibit poor aqueous solubility, with LogS values ranging from − 5.945 to − 6.754 (ESOL) and − 7.24 to − 8.84 (Ali), compared to allopurinol (− 2.80 and − 0.42, respectively). According to the Silicon-IT solubility class, all test compounds are classified as “poorly soluble” a major hurdle for oral bioavailability. Consistently, LogP values (3.165–4.404) exceed the optimal range (1–3), and LogD values (2.88–3.75) are suboptimal. While the compounds pass the Lipinski rule of five, most fail the Golden Triangle rule (MW 200–500, LogP 0–3), and several violate the Pfizer rule (e.g., 5d). Predicted human intestinal absorption (HIA) is negligible for all test compounds (values near 0), and Caco-2 permeability is low (− 5.0 to − 5.4 log cm/s). All compounds show very high plasma protein binding (> 97%), leaving minimal free fraction (Fu < 2%), which may reduce therapeutic efficacy. Blood–brain barrier (BBB) penetration is low for most (except 5d), which is acceptable for peripheral targets.

**Table 10:** Prediction of pharmacokinetics and physicochemical properties of compounds compared with reference drug (allopurinol).

Id	ID	5a	5b	5c	5d	6a	6b	6c	Allopurinol	Id	ID	5a	5b	5c	5d	6a	6b	6c	Allopurinol
Physicochemical Properties	MW	475.060	504.050	505.070	526.990	504.090	520.080	542.000	136.04	Metabolism	CYP1A2-inh	0.999	0.986	1.000	1.000	0.996	0.999	1.000	0.01
	Vol	446.954	464.104	473.040	468.586	481.167	489.957	485.502	120.1549		CYP1A2-sub	0.429	0.986	0.414	0.964	0.606	0.542	0.598	0.00
	Dense	1.063	1.086	1.068	1.125	1.048	1.061	1.116	1.132205		CYP2C19-inh	0.887	0.579	0.842	1.000	0.996	0.936	1.000	0.00
	nHA	7.000	9.000	8.000	6.000	8.000	9.000	7.000	5		CYP2C19-sub	0.004	0.029	0.083	0.028	0.005	0.082	0.025	0.00
	nHD	1.000	0.000	1.000	0.000	0.000	1.000	0.000	2		CYP2C9-inh	0.162	0.018	0.045	0.919	0.937	0.785	0.982	0.00
	TPSA	93.2	116.1	102.5	73.0	105.5	125.7	96.3	74.43		CYP2C9-sub	0.369	0.018	0.419	0.167	0.311	0.622	0.355	0.01
	nRot	4.000	5.000	5.000	4.000	7.000	7.000	6.000	0		CYP2D6-inh	0.000	0.001	0.000	0.005	0.032	0.008	0.215	0.00
	nRing	5.000	5.000	5.000	5.000	4.000	4.000	4.000	2		CYP2D6-sub	0.003	0.001	0.011	0.000	0.202	0.010	0.008	0.00
	MaxRing	13.000	13.000	13.000	13.000	9.000	9.000	9.000	9		CYP3A4-inh	0.010	0.023	0.060	0.061	0.031	0.084	0.097	0.00
	nHet	9.000	11.000	10.000	10.000	10.000	11.000	11.000	5		CYP3A4-sub	0.066	0.042	0.062	0.128	0.000	0.010	0.015	0.00
	fChar	0.000	0.000	0.000	0.000	0.000	0.000	0.000	0	Excretion	CYP2B6-inh	0.054	0.953	0.908	0.998	0.981	0.960	1.000	0.00
	nRig	29.0	30.0	29.0	29.0	26.0	26.0	26.0	11		CYP2B6-sub	0.000	0.000	0.000	0.000	0.000	0.000	0.000	0.00
	Flex	0.138	0.167	0.172	0.138	0.269	0.269	0.231	0	Toxicity	CYP2C8-inh	0.984	0.999	0.999	1.000	1.000	0.964	1.000	0.00
	nStereo	0.000	0.000	0.000	0.000	0.000	0.000	0.000	0		LM-human	0.136	0.372	0.313	0.171	0.127	0.205	0.210	0.00
Solubility	LogS	− 6.754	− 6.203	− 6.753	− 6.366	− 5.945	− 6.423	− 6.559	− 2.80		cl-plasma	5.839	6.157	6.599	5.594	6.283	5.266	4.244	10.85
	LogD	3.242	3.313	2.976	3.679	3.350	2.882	3.748	− 0.14		t0.5	0.583	0.611	0.808	0.898	0.803	0.951	1.113	2.04
	LogP	3.812	3.637	3.487	4.321	3.651	3.165	4.404	− 0.39		BCF	1.719	1.941	1.666	2.645	2.081	1.616	2.762	0.05
	ESOL Log S	− 6.78	− 6.98	− 6.85	− 8.11	− 6.28	− 6.14	− 7.39	− 0.93		IGC50	4.957	5.147	4.806	5.333	4.780	4.563	5.078	0.05
	Ali Log S	− 7.59	− 8.31	− 7.75	− 8.84	− 7.24	− 7.29	− 8.37	− 0.42		LC50DM	6.369	6.791	6.055	7.079	6.777	5.844	7.039	2.19
	Silicon-IT class	Poorly	Poorly	Poorly	Poorly	Poorly	Poorly	Poorly	soluble		LC50FM	6.105	6.550	5.777	6.962	6.263	5.507	6.782	2.74
drug-likeness	Lipinski Rule	Accepted	Accepted	Accepted	Accepted	Accepted	Accepted	Accepted	Accepted		hERG	0.489	0.672	0.590	0.811	0.633	0.472	0.729	0.07
	Pfizer Rule	Accepted	Accepted	Accepted	Rejected	Accepted	Accepted	Accepted	Accepted		hERG-10um	0.723	0.812	0.769	0.865	0.748	0.747	0.842	0.26
	Golden	Accepted	Rejected	Rejected	Rejected	Rejected	Rejected	Rejected	Rejected		DILI	0.960	0.999	0.966	0.995	0.976	0.950	0.994	0.97
Absorption	Pgp-inh	0.481	0.770	0.506	0.930	0.997	0.845	0.972	0.00	Toxicophore Rules	Ames	0.368	0.839	0.397	0.220	0.326	0.313	0.166	0.40
	Pgp-sub	0.000	0.000	0.000	0.000	0.000	0.000	0.000	0.04		ROA	0.359	0.617	0.432	0.525	0.278	0.359	0.458	0.74
	HIA	0.000	0.000	0.000	0.000	0.001	0.000	0.000	0.01		FDAMDD	0.868	0.823	0.823	0.851	0.598	0.640	0.742	0.24
	F (20%)	0.056	0.011	0.145	0.001	0.074	0.019	0.000	0.01		SkinSen	0.703	0.927	0.642	0.645	0.935	0.965	0.965	0.12
	F (30%)	0.845	0.500	0.877	0.387	0.873	0.585	0.055	0.00		Carcinogenicity	0.323	0.563	0.473	0.473	0.339	0.316	0.349	0.53
	Caco-2	− 5.223	− 5.190	− 5.271	− 5.049	− 5.170	− 5.391	− 5.067	− 4.88		EC	0.000	0.000	0.000	0.000	0.000	0.000	0.000	0.00
	MDCK	− 4.768	− 4.744	− 4.828	− 4.778	− 4.770	− 4.794	− 4.744	− 4.88		EI	0.200	0.342	0.134	0.036	0.178	0.206	0.054	0.95
Distribution	BBB	0.423	0.019	0.029	0.844	0.001	0.003	0.213	0.07	Medicinal Chemistry	Respiratory	0.737	0.748	0.624	0.509	0.626	0.635	0.584	0.94
	PPB	98.031	97.925	97.900	98.533	97.472	97.933	99.069	2.52		QED	0.270	0.129	0.246	0.183	0.234	0.253	0.192	0.519
	VDss	− 0.163	− 0.189	− 0.222	− 0.043	− 0.068	− 0.210	− 0.160	− 0.24		Synth	2.902	2.899	2.911	2.915	3.181	3.292	3.306	2.00
	Fu	1.714	1.847	1.852	1.007	1.792	1.665	0.642	91.81		Fsp3	0.042	0.042	0.080	0.042	0.120	0.120	0.083	0.00

**Table 11:** Prediction of toxicity risks and oral toxicity prediction results of compounds and drugs.

No.	Ligand	Toxicity risks				Physicochemical properties					
		Mutagenic	Tumorigenic	Irritant	Reproductive	CLogP	Solubility	Molecular weight	TPSA	Drug likeness	Drug score
1	**5a**	(−)	(−)	(−)	(−)	4.73	− 7.44	475.0	93.27	5.23	0.33
2	**5b**	(−)	(−)	(−)	(−)	3.98	− 8.19	504.0	118.8	− 5.14	0.17
3	**5c**	(−)	(−)	(−)	(−)	4.66	− 7.45	505.0	102.5	5.53	0.32
4	**5d**	(−)	(−)	(−)	(−)	6.29	− 9.20	527.0	73.04	5.09	0.22
5	**6a**	(−)	(−)	(−)	(−)	4.37	− 7.15	504.0	105.5	1.61	0.15
6	**6b**	(−)	(−)	(−)	(−)	3.99	− 6.85	520.0	125.7	1.88	0.33
7	**6c**	(−)	(−)	(−)	(−)	5.62	− 8.60	542.0	96.30	1.32	0.21
8	**Allopurinol**	(−)	(−)	(−)	(+)	− 1.19	− 1.30	136.0	70.14	5.29	0.59

Metabolism and toxicity risks. Major concerns arise from CYP inhibition profiles: all compounds show high probability of inhibiting CYP1A2 (0.986–1.000), CYP2C8 (0.964–1.000), and CYP2B6 (0.908–1.000). Several also inhibit CYP2C19 (e.g., 5d, 6c: 1.000) and CYP2C9 (e.g., 6a, 6c: 0.937–0.982). This broad CYP inhibition raises the risk of drug–drug interactions. Consistent with these findings, the predicted Drug-Induced Liver Injury (DILI) risk is very high for all compounds (0.950–0.999), comparable to allopurinol (0.97) but concerning for further development. Encouragingly, all compounds test negative for mutagenicity, tumorigenicity, irritancy, and reproductive toxicity (Table 11). The hERG risk is moderate to high (0.472–0.811), suggesting potential cardiotoxicity liability. Overall ADMET assessment and path forward. While the docking and MD simulations demonstrate that compounds 5a–d and 6a–c effectively bind to their respective targets (IL-6, TNF-α, MMP-9, NLRP3) with favorable free energies, the ADMET profiling reveals substantial pharmacokinetic and toxicity challenges, particularly poor solubility, negligible oral absorption, high CYP inhibition, DILI risk, and low drug-likeness. Therefore, these compounds should be regarded as lead optimization starting points rather than direct drug candidates. Future medicinal chemistry efforts should focus on: (i) improving solubility via introduction of polar groups or prodrug strategies; (ii) reducing CYP inhibition by modifying aromatic ring systems or incorporating heteroatoms; (iii) lowering logP and increasing fraction of sp^3^ carbons (currently Fsp^3^ ≤ 0.12) to enhance drug-likeness; and (iv) addressing hERG liability. The reference drug allopurinol demonstrates superior ADMET properties (excellent solubility, no CYP inhibition, low toxicity), providing a benchmark for optimization goals.

Conclusion for ADMET: While the docking and MD simulations demonstrate that these compounds effectively bind to IL-6, TNF-α, MMP-9, and NLRP3 with favorable free energies, their ADMET profiles indicate that they are not ready as drug candidates but rather serve as lead optimization starting points. Future medicinal chemistry should prioritize: (i) improving solubility via polar group introduction or prodrug strategies; (ii) reducing CYP inhibition by modifying planar aromatic rings; (iii) lowering logP (currently 3.2–4.4) and increasing Fsp^3^ (currently ≤ 0.12); and (iv) mitigating hERG liability. Allopurinol provides a benchmark for the desired ADET profile.

### Molecular dynamics simulation (MDS)

**Fig. 19:**
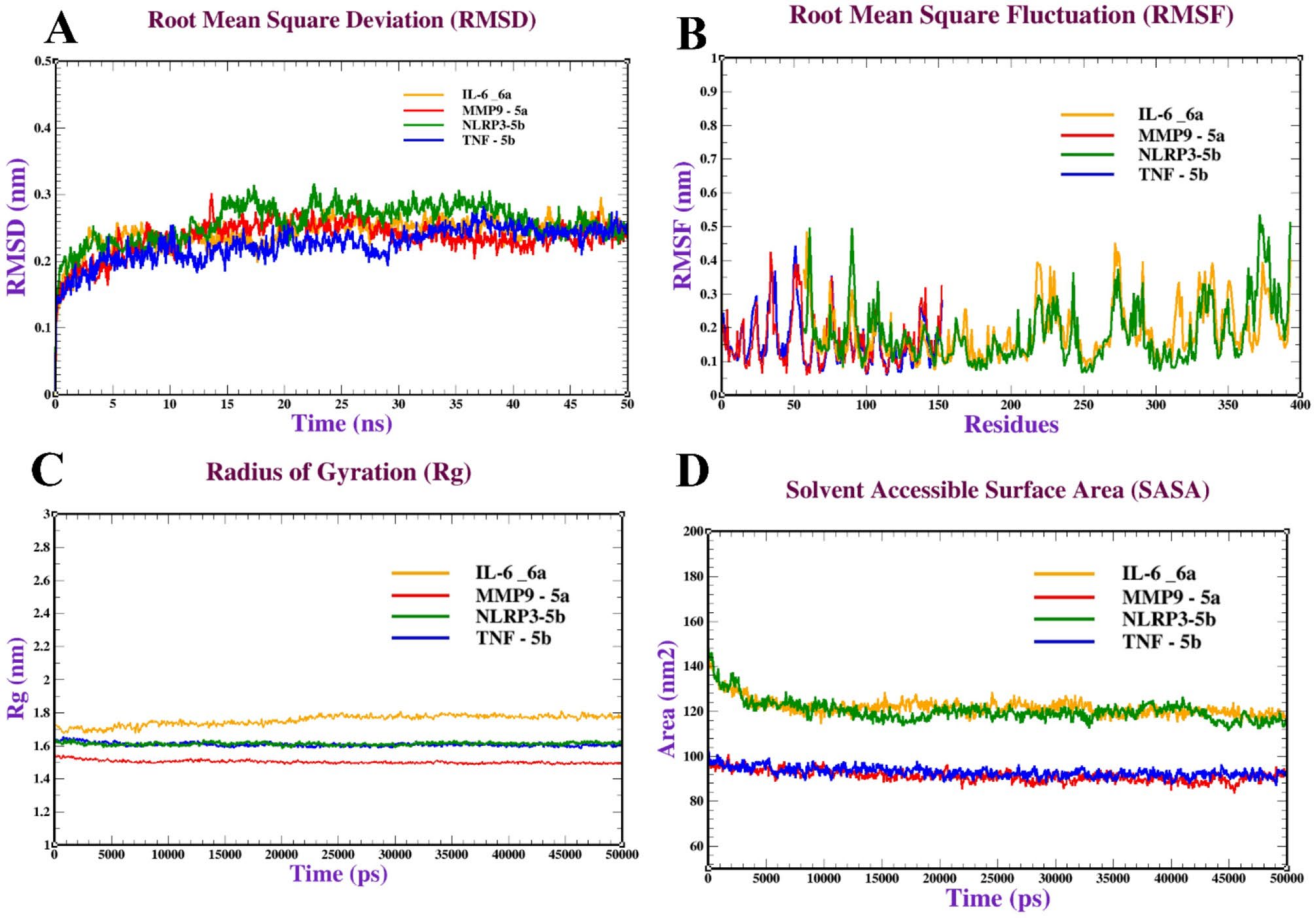
Molecular dynamics of IL-6 with 6a, MMP9 with 5a, NLRP3 with 5b, and TNF with 5b: (**A**) RMSD, (**B**) RMSF, (**C**) radius of gyration (Rg), and (**D**) SASA.

Molecular Dynamics (MD) simulation results for the four top ligand–protein complexes IL-6 with **6a**, MMP9 with **5a**, NLRP3 with **5b**, and TNF with **5b** reveal a highly consistent and stable pattern of binding over a 50-picosecond (ps) simulation period. The structural stability of these protein–ligand complexes and their free forms was evaluated by analyzing the root mean square deviation (RMSD) over a 50 ns simulation period. The RMSD analysis evaluates the deviations of protein backbone atoms from their initial configurations, with values settling between 0.10 and 0.31 nm after initial fluctuations, showing structural constancy with time. TNF with **5b** had an RMSD ranging from 0.15 to 0.25 nm, which stabilized after about 30 ns, showing strong structural integrity with minor conformational changes. In contrast, for NLRP3 with **5b**, the RMSD ranged from 0.20 to 0.31 nm, stabilizing after roughly 25 ns, and demonstrating strong structural flexibility with conformation changes. IL-6 with **6a** and MMP9 with **5a,** have slightly higher RMSD values than the free equivalent, indicating minimal structural changes during ligand binding. Overall, all systems demonstrated acceptable stability, with RMSD values remaining below 0.31 nm, suggesting that binding of ligands does not significantly alter protein structures over 50 ns (Fig. 19A). Root mean square fluctuation (RMSF) analysis revealed the flexibility of individual residues during the simulations. Complexed states of IL-6 with **6a** and NLRP3 with **5b** exhibited higher variations (up to 0.40 nm) than Free states, indicating increased local mobility, most likely due to ligand interactions. RMSF values in all systems remained below 0.5 nm, with occasional peaks up to 0.50 nm in specific residues, indicating localized flexibility, particularly in loop areas (Fig. 19B). The radius of gyration (Rg) was utilized to determine the compactness of protein structures, which indicates their overall shape stability. Rg values ranged from 1.55 to 1.75 nm across all systems, indicating stable conformations in both free and bound states. Rg values in the MMP9 with **5a**, system (free and bound) remained between 1.55 and 1.60 nm, indicating a constant compact structure. The complex’s Rg remains lower and more constant, implying that ligand binding promotes a more compact and stable folded form. The IL-6 with **6a**, NLRP3 with **5b**, and TNF with **5b** revealed somewhat higher Rg values, with a modest increase after 25 ns, indicating a slight structural expansion due to **5b** binding (Fig. 19C). Solvent-accessible surface area (SASA) analysis assessed the proteins’ exposure to the solvent environment. The SASA values of MMP9 with **5a** and TNF with **5b** were around 95–100 nm^2^ and 90–105 nm^2^, respectively, with slight fluctuations indicating sustained solvent exposure. The complex has a smaller and more stable SASA, showing that ligand binding reduces solvent exposure, either due to burial of hydrophobic areas or overall structural tightness (Fig. 19D). These findings are consistent with prior MD studies on anticancer protein–ligand systems^46,47^, which also used RMSD, RMSF, SASA, and hydrogen bond metrics to assess complex stability and dynamic behavior.

**Fig. 20:**
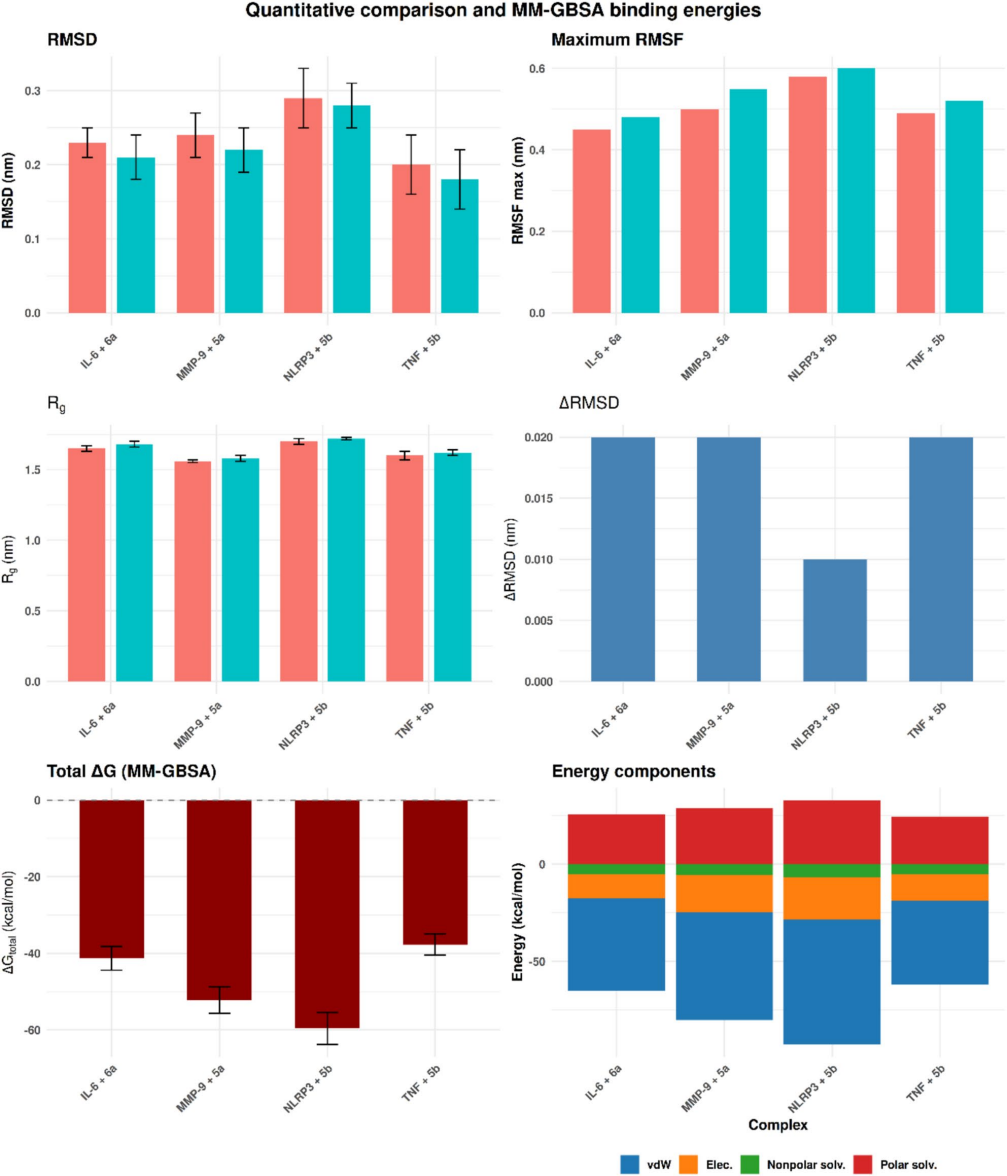
MM-GBSA calculations using gmx-MMPBSA and quantitative comparison of bound vs. free systems for all four complexes.

Additionally, we compared RMSD, RMSF, and radius of gyration (Rg) between free and bound states for all four complexes (Table 12 and Fig. 20). For IL6-6a, the RMSD increased marginally from 0.21 ± 0.03 nm (free) to 0.23 ± 0.03 nm (bound), with similar trends observed for TNF + 5b (0.18–0.20 nm), MMP-9 + 5a (0.22–0.24 nm), and NLRP3-5b (0.28–0.29 nm). The ΔRMSD values (< 0.03 nm across all systems) indicate that ligand binding does not induce major conformational disruption. Notably, maximum RMSF values were consistently lower in bound systems (NLRP3-5b: 0.60 nm free vs. 0.58 nm bound), suggesting reduced residue-level flexibility upon ligand binding. Similarly, Rg values decreased slightly in all bound complexes (IL6-6a: 1.68–1.65 nm), indicating ligand-induced conformational compaction. These quantitative comparisons collectively suggest that all four ligands stabilize their target proteins without causing structural destabilization. Furthermore, we performed MM-GBSA calculations using gmx-MMPBSA on the final 100 ns of each trajectory (1000 frames). As summarized in Table 13, all complexes exhibited favorable negative binding free energies. NLRP3-5b showed the most favorable ΔG_total (− 59.6 ± 4.2 kcal/mol), followed by MMP-9 + 5a (− 52.2 ± 3.5 kcal/mol), IL-6 + 6a (− 41.3 ± 3.1 kcal/mol), and TNF + 5b (− 37.7 ± 2.8 kcal/mol). In all cases, van der Waals contributions (ΔG_vdW: − 43.1 to − 64.2 kcal/mol) and electrostatic contributions (ΔG_elec: − 12.2 to − 21.9 kcal/mol) favored binding, while polar solvation energy (ΔG_polar solv: + 24.4 to + 32.9 kcal/mol) opposed it, a characteristic signature of hydrophobic-driven binding interactions. Nonpolar solvation contributions were modest and favorable (− 5.1 to − 6.6 kcal/mol). The superior binding affinity of NLRP3 + 5b is consistent with its lower RMSF values and compact Rg, suggesting that tighter binding correlates with enhanced conformational stabilization. These energetic profiles confirm that all four ligands are effective binders, with MMP-9 + 5a and NLRP3 + 5b demonstrating particularly strong binding affinities.

**Table 12:** Quantitative comparison of bound vs. free systems for all four complexes:

Complexes	RMSD (nm) free	RMSD (nm) bound	ΔRMSD	RMSF max free (nm)	RMSF max bound (nm)	Rg free (nm)	Rg bound (nm)
IL-6 + 6a	0.21 ± 0.03	0.23 ± 0.03	+ 0.02	0.48	0.45	1.68 ± 0.02	1.65 ± 0.02
TNF + 5b	0.18 ± 0.02	0.20 ± 0.03	+ 0.02	0.52	0.49	1.62 ± 0.02	1.60 ± 0.01
MMP-9 + 5a	0.22 ± 0.04	0.24 ± 0.03	+ 0.02	0.55	0.50	1.58 ± 0.02	1.56 ± 0.01
NLRP3 + 5b	0.28 ± 0.04	0.29 ± 0.04	+ 0.01	0.60	0.58	1.72 ± 0.03	1.70 ± 0.02

Bound systems show slightly lower RMSF and Rg values, indicating ligand-induced stabilization and compaction. ΔRMSD < 0.03 nm suggests no major conformational disruption.

**Table 13:** MM-GBSA calculations using gmx-MMPBSA for all four complexes:

Complexes	ΔG total (kcal/mol)	ΔG vdW	ΔG elec	ΔG polar solv	ΔG nonpolar solv
IL-6 + 6a	− 41.3 ± 3.1	− 47.6	− 12.2	+ 25.5	− 5.2
TNF + 5b	− 37.7 ± 2.8	− 43.1	− 13.8	+ 24.4	− 5.1
MMP-9 + 5a	− 52.2 ± 3.5	− 55.3	− 19.1	+ 28.8	− 5.7
NLRP3 + 5b	− 59.6 ± 4.2	− 64.2	− 21.9	+ 32.9	− 6.6

## Experimental

### Materials and measurements

All of the chemicals and reagents used were obtained from Aldrich Chemical Co., Inc. (WI, USA). For thin-layer chromatography (TLC), precoated (0.25 mm) silica gel GF254 plates (Merck, Darmstadt, Germany) were used. An ultraviolet lamp (254 nm) was used to observe the spots. The methylene chloride: methanol (1:0.5) elution method was used. All melting points (MPs) were recorded using an uncorrected Stuart melting point apparatus (SMP 30) (Bibby Scientific, Staffordshire, UK). IR spectra (KBr) were recorded using the Thermo Fisher Scientific instrument. Model: is10 (USA) at the National Research Centre, Doki, Egypt. A Bruker NMR spectrometer was used to record ^1^H-NMR and ^13^C-NMR spectra in DMSO-*d*_*6*_ at 400 MHz (^1^H-NMR) and 100 MHz (^13^C-NMR) (Zagazig University, Zagazig, Egypt) using tetramethyl silane as an internal standard. Peak splitting patterns were indicated by the following symbols, when applicable: s = singlet and d = doublet. A PerkinElmer CHN 2400 was used to implement C, H, and N concerns. The Mycology and Biotechnology Regional Center, Al-Azhar University, Egypt, performed mass spectra on the direct inlet portion to the mass analyzer in a Thermo Scientific (Thermo Fisher Scientific Ink, MA, USA) GC–MS model ISQ. The crystalline structure of the produced NiO nanoparticles was analyzed using X-ray diffraction (XRD; BRUKER D8 Advance, Germany) employing Cu–Kα radiation (λ = 1.5406 Å) throughout a 2θ range of 10–80° at a scanning rate of 2°/min. Phase identification was conducted by comparison with standard JCPDS reference data. Functional groups were examined using Fourier-transform infrared spectroscopy (FTIR; Perkin Elmer Spectrum 1600, USA) in the 400–4000 cm^−1^ range at ambient temperature. Morphological and structural characteristics were analyzed utilizing high-resolution transmission electron microscopy (HR-TEM; JEOL JEM-1230, Japan) at 120 kV and field-emission scanning electron microscopy (FE-SEM; JEOL JXA-840A, Japan) at 15 kV. For transmission electron microscopy investigation, materials were ultrasonically dispersed in distilled water for one hour and subsequently placed onto carbon-coated copper grids. The elemental composition was ascertained by energy-dispersive X-ray spectroscopy (EDX). The textural and porous characteristics were assessed by nitrogen adsorption–desorption isotherms at − 196 °C utilizing Micrometrics ASAP 2020 and Quantachrome NOVA touch 4LX analyzers. Before measurement, about 100 mg of the sample was degassed under pressure at 150 °C for 6 h. The Brunauer–Emmett–Teller (BET) surface area (SBET), total pore volume (VP), and average pore diameter (RP) were computed utilizing TouchWin™ software. Ultra-high quality nitrogen (99.999%) was utilized as the adsorption gas, and the void volume was calibrated using helium displacement.

### Synthesis of NiO nanoparticles

Nickel oxide (NiO) nanoparticles were synthesized using a straightforward co-precipitation method. Initially, 500 mL of a 1.0 M aqueous solution of nickel acetate [Nickel (II) acetate A.R. (Ni(CH_3_COO)_2_.4H_2_O), Alpha ChemiKa, India) was heated to 60°C under continuous magnetic stirring at 400 rpm. A 2.0 M NaOH solution [Merck-Germany] was then added dropwise at a rate of 0.5 mL/min until the pH of reaction up to 8, leading to the instantaneous formation of a lustrous green precipitate of Ni(OH)₂ gel. The mixture was aged for 2 h under constant stirring to ensure complete precipitation and enhance crystallinity. The resulting solid was collected by centrifugation, washed repeatedly with deionized water and ethanol to eliminate ionic impurities, and subsequently dried at 100 °C for 5 h. Finally, the dried Ni(OH)₂ precursor was calcined in a muffle furnace at 500 °C for 3 h to yield crystalline NiO nanoparticles^48^.

### Chemistry

#### 2-(2,4-Dichlorobenzylidene)malononitrile (1)

A mixture of 2,4-dichlorobenzaldehyde (0.01 mol) and malononitrile (0.01 mol) was dissolved in absolute ethanol (30 mL) in the presence of a few drops (3–4 drops) of piperidine as a catalyst. The reaction mixture was refluxed for 2 h, and the progress of the reaction was monitored by thin-layer chromatography (TLC) using dichloromethane as the eluent. After completion of the reaction, the mixture was allowed to cool to room temperature. The resulting precipitate was collected by filtration under reduced pressure, washed with cold ethanol, and recrystallized from ethanol to afford compound **1** as a buff crystalline solid. Yield: 97%; m.p. 139–142 °C^34^.

#### 5-Amino-7-(2,4-dichlorophenyl)-7,8-dihydro-[1,2,4]triazolo[4,3-*a*]pyrimidine-6-carbonitrile (2)

Compound **1** (0.01 mol) and 3-amino-1,2,4-triazole (0.01 mol) were dissolved in absolute ethanol (50 mL). The reaction was carried out under reflux conditions in the presence of nickel oxide nanoparticles (NiO NPs, 7.5 mg) as a heterogeneous catalyst. The reaction progress was monitored by TLC using dichloromethane as the eluent. After completion, the reaction mixture was cooled to room temperature. The precipitate was filtered, washed with cold ethanol, and dried. The crude product was purified by recrystallization from hot ethanol to give compound **2** as a yellow crystalline solid. Yield: 95%; m.p. 274–277 °C^35^, ^1^H-NMR (400 MHz, DMSO-*d*_*6*_, δ, ppm): 5.77 (s, 1H, CH _Pyrimidine_), 7.29 (s, 2H, NH_2,_ exchangeable by D_2_O), 7.45 (s, 1H, Ar–H), 7.64–7.66 (d, *J* = 8 Hz, 1H, Ar–H), 7.85–7.87 (d, *J* = 8.4 Hz, 1H, Ar–H), 7.71 (s, 1H, CH _Triazolo_), 8.67 (s, 1H, NH, exchangeable by D_2_O); Anal. calcd. For C_12_H_8_Cl_2_N_6_: C, 46.93; H, 2.63; Cl, 23.08; N, 27.36. Found: C, 46.96; H, 2.60; Cl, 23.05; N, 27.34.

#### 1-(4-Amino-5-(2,4-dichlorophenyl)-2-methylpyrido[3,2-e][1,2,4]triazolo[4,3-a]pyrimidin-3-yl)ethan-1-one (3)

Compound **2** (0.2 mol) was reacted with acetylacetone (0.2 mol) in dimethylformamide (DMF, 50 mL). The reaction mixture was heated under reflux for 11 h with continuous stirring. After completion of the reaction, as monitored by TLC using dichloromethane as the eluent, the mixture was allowed to cool to room temperature and then poured onto crushed ice with stirring until complete precipitation of the product occurred. The resulting solid was collected by filtration, washed with cold water, and dried. The crude product was further purified by recrystallization from ethanol to afford compound **3** as brown crystals. Yield: 83%; m.p. 162–164 °C. υmax/cm^−1^ (KBr): 3435, 3368 (NH_2_), 3124 (CH for aromatic), 2921 (CH for aliphatic), 1724 (C=O _ketone_), 794 (C–Cl); ^1^H-NMR (DMSO-*d*_6_, 400 MHz, ppm): *δ* = 2.09, 2.50 (s, 6H, 2CH_3_), 7.30 (s, 2H, NH_2_, exchangeable by D_2_O), 7.63–7.65 (d, *J* = 8 Hz, 1H, Ar–H), 7.84–7.86 (d, *J* = 8 Hz, 1H, Ar–H), 8.67 (s, 1H, Ar–H), 9.45 (s, 1H, CH _Triazolo_); ^13^C-NMR (125 MHz, DMSO-*d*_*6*_, δ, ppm): 21.64, 29.79, 78.45, 114.73, 128.20, 129.58, 132.15, 132.65, 135.57, 135.92, 151.16, 155.23, 156.30, 158.51, 163.24, 167.89, 194.63; MS: m/z (intensity 100%): 387 (6) [M^+^], 389 (2) [M^2+^], 391 (2) [M^4+^], 383 (100); Anal. Calcd. For C_17_H_12_Cl_2_N_6_O (387.22): C, 52.73; H, 3.12; N, 21.70; Found: C, 52.80; H, 3.17; N, 21.80.

#### (*Z*)-7-(2,4-Dichlorophenyl)-5-((4-oxopentan-2-ylidene)amino)-[1,2,4]triazolo[4,3-*a*]pyrimidine-6-carbonitrile (4):

Compound **2** (0.2 mol) was reacted with acetylacetone (0.2 mol) in dimethylformamide (DMF, 50 mL). The reaction mixture was heated under reflux with stirring for 3 h. The progress of the reaction was monitored by TLC using dichloromethane as the eluent. After completion, the reaction mixture was allowed to cool to room temperature and then poured onto crushed ice with continuous stirring until complete precipitation of the product occurred. The resulting solid was collected by filtration, washed with cold water, and dried. The crude product was purified by recrystallization from ethanol to afford compound **4** as brown crystals. Yield: 97%; m.p. 108–111 °C.; υmax/cm^−1^ (KBr): 3436 (NH), 3001 (CH for aromatic), 2920 (CH for aliphatic), 2219 (CN_Nitrile_), 1701 (C=O_ketone_), 778 (C–Cl); ^1^H-NMR (DMSO-*d*_6_, 400 MHz, ppm): *δ* = 2.15, 2.21 (s, 6H, 2CH_3_), 2.63 (s, 2H, CH_2_), 7.26–7.28 (d, *J* = 8 Hz, 2H, Ar–H), 7.41 (s, 1H, Ar–H), 7.94 (s, 1H, CH _Triazolo_); ^13^C-NMR (100 MHz, DMSO-*d*_*6*_, δ, ppm): 15.36, 30.34, 46.81, 105.34, 128.21, 129.65, 132.38, 135.75, 139.34, 142.08, 148.46, 155.08, 156.59, 165.30, 169.55, 203.04; MS: m/z (intensity 100%): 391 (23) [M^+^], 389 (23) [M^2+^], 387 (30) [M^+^], 109 (100); Anal. Calcd. For C_17_H_12_Cl_2_N_6_O (387.22): C, 52.73; H, 3.12; N, 21.70; Found: C, 52.83; H, 3.19; N, 21.80.

#### (*E*)-1-(4-Amino-5-(2,4-dichlorophenyl)-2-methylpyrido[3,2-*e*][1,2,4]triazolo[4,3-*a*]pyrimidin-3-yl)-3-(aryl)prop-2-en-1-one (5a-d)

Compound **3** (0.05 mol) was dissolved in dimethylformamide (DMF, 10 mL). To this solution, an equimolar amount (0.05 mol) of the appropriate aromatic aldehyde derivatives (2-hydroxybenzaldehyde, 4-nitrobenzaldehyde, 4-hydroxy-3-methoxybenzaldehyde, or 2,4-dichlorobenzaldehyde) was added, followed by sodium hydroxide (0.05 mol) as a basic catalyst. The reaction mixture was stirred at room temperature (20 °C) for 20–27 h. After completion of the reaction (monitored by TLC using dichloromethane as the eluent), the mixture was allowed to stand in a refrigerator for 7–27 days to complete crystallization. The resulting precipitate was obtained by filtration, washed with cold water, and dried in an oven. The crude products were purified by recrystallization from ethanol, followed by washing with acetone to afford the final compounds **5a–d**.

#### (*E*)-1-(4-amino-5-(2,4-dichlorophenyl)-2-methylpyrido[3,2-*e*][1,2,4]triazolo[4,3-*a*]pyrimidin-3-yl)-3-(2-hydroxyphenyl)prop-2-en-1-one (5a)

The reaction mixture was stirred for 20 h and then kept in a refrigerator for 7 days. A black crystalline solid was obtained in 82% yield; m.p. 264–266 °C; υmax/cm^−1^ (KBr): 3486 (OH), 3450, 3422 (NH_2_), 3058 (CH for aromatic), 2928 (CH for aliphatic), 1722 (C=O _Ketone_), 778 (C–Cl); ^1^H-NMR (DMSO-*d*_6_, 400 MHz, ppm): *δ* = 1.66 (s, 3H, CH_3_), 6.88 (s, 2H, NH_2_), 7.28 (s, 4H, Ar–H), 7.45–7.50 (d, *J* = 18 Hz, 2H, 2CH), 7.59–7.61 (d, *J* = 8 Hz, 2H, Ar–H), 8.03 (s, 1H, Ar–H), 8.53 (s, 1H, CH _Triazolo_); MS (m/z, %): 491 [M]⁺, 493 [M^2+^]⁺, 495 [M^4+^]⁺; base peak at m/z 125 (100%); Anal. Calcd. for C_24_H_16_Cl_2_N_6_O_2_ (491.33): C, 58.67; H, 3.28; N, 17.10; Found: C, 58.66; H, 3.30; N, 14.20.

#### (*E*)-1-(4-Amino-5-(2,4-dichlorophenyl)-2-methylpyrido[3,2-*e*][1,2,4]triazolo[4,3-*a*]pyrimidin-3-yl)-3-(4-nitrophenyl)prop-2-en-1-one (5b)

The reaction mixture was stirred for 27 h and then kept in a refrigerator for 19 days. A brown crystalline solid was obtained in 93% yield; m.p. 256–260 °C. υmax/cm^−1^ (KBr): 3344, 3321 (NH_2_), 3088 (CH for aromatic), 2928 (CH for aliphatic), 1726 (C = O _Ketone_), 1547, 1376 (NO_2_), 645 (C–Cl); ^1^H-NMR (DMSO-*d*_6_, 400 MHz, ppm): *δ* = 3.81 (s, 3H, CH_3_), 6.72 (s, 2H, NH_2_), 7.-01–7.03 (d, *J* = 7.2 Hz, 2H, Ar–H), 7.52 (s, 1H, Ar–H), 7.92–7.94 (d, *J* = 8.4 Hz, 2H, Ar–H), 7.97–7.99 (d, *J* = 7.2 Hz, 2H, Ar–H), 8.36–8.40 (d, *J* = 14.8 Hz, 2H, 2CH), 9.73 (s, 1H, CH_Triazolo_); MS (m/z, %): 520 [M]⁺, 522 [M^2+^]⁺, 524 [M^4+^]⁺; base peak at m/z 44 (100%); Anal. Calcd. for C_24_H_15_Cl_2_N_7_O_3_ (520.33): C, 55.40; H, 2.91; N, 18.84; Found; C, 55.50; H, 2.92; N, 18.92.

#### (*E*)-1-(4-Amino-5-(2,4-dichlorophenyl)-2-methylpyrido[3,2-*e*][1,2,4]triazolo[4,3-*a*]pyrimidin-3-yl)-3-(4-hydroxy-3-methoxyphenyl)prop-2-en-1-one (5c)

The reaction mixture was stirred for 21 h and then kept in a refrigerator for 27 days. A yellow crystalline solid was obtained in 86% yield; m.p. 250–252 °C; υmax/cm^−1^ (KBr): 3448 (OH), 3424, 3328 (NH_2_), 3034 (CH for aromatic), 2928, 2851 (CH for aliphatic), 1738 (C=O_Ketone_), 647 (C–Cl); ^1^H-NMR (DMSO-*d*_6_, 400 MHz, ppm): *δ* = 2.68 (s, 3H, CH_3_), 3.82 (s, 3H, OCH_3_), 6.34 (s, 2H, NH_2_), 7.00–7.02 (d, *J* = 8 Hz, 2H, Ar–H), 7.35–7.39 (d, *J* = 14 Hz, 2H, 2CH), 7.58–7.60 (d, *J* = 8 Hz, 2H, Ar–H), 7.75 (s, 1H, Ar–H), 8.55 (s, 1H, CH _Triazolo_), 9.74 (s, 1H, OH); ^13^C-NMR (100 MHz, DMSO-*d*_*6*_, δ, ppm): 31.15, 42.99, 114.98, 115.28, 123.53, 127.36, 128.14, 128.32, 130.43, 131.64, 131.93, 133.48, 136.16, 140.69, 140.87, 143.34, 150.69, 151.57, 152.78, 161.48, 168.66, 197.10; MS (m/z, %): 521 [M]⁺, 523 [M^2+^]⁺, 525 [M^4+^]⁺; base peak at m/z 429 (100%).; Anal. Calcd. for C_25_H_18_Cl_2_N_6_O_3_ (521.36): C, 57.59; H, 3.48; N, 16.12; Found: C, 57.69; H, 3.51; N, 16.18.

#### (*E*)-1-(4-Amino-5-(2,4-dichlorophenyl)-2-methylpyrido[3,2-*e*][1,2,4]triazolo[4,3-*a*]pyrimidin-3-yl)-3-(2,4-dichlorophenyl)prop-2-en-1-one (5d)

The reaction mixture was stirred for 21 h and then kept in a refrigerator for 27 days. An orange powder was obtained in 89% yield; m.p. 254–258 °C; υmax/cm^−1^ (KBr): 3394, 3335 (NH_2_), 3067 (CH for aromatic), 2916 (CH for aliphatic), 1712 (C=O _Ketone_), 647 (C–Cl); ^1^H-NMR (DMSO-*d*_6_, 400 MHz, ppm): *δ* = 1.24 (s, 3H, CH_3_), 6.89 (s, 2H, NH_2_), 7.09–7.12 (d, *J* = 10 Hz, 1H, Ar–H), 7.25 (s, 1H, Ar–H), 7.52–7.54 (d, *J* = 7.2 Hz, 1H, Ar–H), 7.59–7.61 (d, *J* = 7.6 Hz, 1H, Ar–H), 7.63–7.68 (d, *J* = 18 Hz, 1H, CH), 7.71–7.73 (d, *J* = 8.8 Hz, 1H, Ar–H), 7.82–7.86 (d, *J* = 18 Hz, 1H, CH), 7.93 (s, 1H, Ar–H), 8.68 (s, 1H, CH_Triazolo_); ^13^C-NMR (100 MHz, DMSO-*d*_*6*_, δ, ppm): 34.82, 108.91, 111.02, 113.69, 116.03, 118.47, 119.97, 120.30, 126.73, 128.70, 132.75, 136.20, 138.51, 140.23, 144.72, 148.84, 149.64, 154.07, 158.54, 161.53, 191.39; MS (m/z, %): 544 [M]⁺, 546 [M^2+^]⁺, 548 [M^4+^]⁺, 550 [M^6+^]⁺; base peak at m/z 353 (100%); Anal. Calcd. for C_24_H_14_Cl_4_N_6_O (544.22): C, 52.97; H, 2.59; N, 15.44; Found; C, 52.99; H, 2.64; N, 15.49.

#### 7-(2,4-Dichlorophenyl)-5-(((*E*)-6-(aryl)-4-oxohex-5-en-2-ylidene)amino)-[1,2,4]triazolo[4,3-*a*]pyrimidine-6-carbonitrile (6a-c):

General method: Compound **4** (0.01 mol) was dissolved in dimethylformamide (DMF, 20 mL). To this solution, an equimolar amount (0.01 mol) of the appropriate aromatic aldehydes (4-methoxybenzaldehyde, 4-hydroxy-3-methoxybenzaldehyde, or 2,4-dichlorobenzaldehyde) was added, followed by sodium hydroxide (0.01 mol) as a basic catalyst. The reaction mixture was stirred at 0–5 °C in an ice bath for 7–21 h. After completion of the reaction (monitored by TLC using dichloromethane as the eluent), the mixture was further kept at 4 °C in a refrigerator for two weeks to ensure complete crystallization. The resulting precipitate was collected by filtration, washed with cold water, and dried in an oven. The crude products were purified by recrystallization from methanol to afford the target compounds **6a–c.**

#### 7-(2,4-Dichlorophenyl)-5-(((*E*)-6-(4-methoxyphenyl)-4-oxohex-5-en-2-ylidene)amino)-[1,2,4]triazolo[4,3-*a*]pyrimidine-6-carbonitrile (6a)

The reaction mixture was stirred for 7 h, affording a black solid in 90% yield; m.p. 242–246 °C.υmax/cm^−1^ (KBr): 3074 (CH for aromatic), 2935 (CH for aliphatic), 2250 (CN_Nitrile_), 1696 (C=O_Ketone_), 639 (C–Cl); ^1^H-NMR (400 MHz, DMSO-*d*_*6*_, ppm): δ = 2.73 (s, 3H, CH_3_), 2.89 (s, 2H, CH_2_), 3.86 (s, 3H, OCH_3_), 7.01–7.03 (d, *J* = 8.4 Hz, 2H, Ar–H), 7.12–7.14 (d, *J* = 8.4 Hz, 2H, Ar–H), 7.61–7.66 (d, *J* = 18 Hz, 2H, 2CH), 7.86–7.88 (d, *J* = 8.8 Hz, 2H, Ar–H), 7.95 (s, 1H, Ar–H), 9.87 (s, 1H, CH _Triazolo_); ^13^C-NMR (100 MHz, DMSO-*d*_*6*_, δ, ppm): 31.25, 34.89, 56.17, 114.28, 114.99, 118.43, 123.52, 126.77, 130.11, 131.35, 131.81, 132.29, 136.79, 140.81, 146.42, 154.33, 156.33, 158.81, 160.71, 164.52, 191.82; MS (m/z, %): 505 [M]⁺, 507 [M^2+^]⁺, 509 [M^4+^]⁺; base peak at m/z 146 (100%, fragment ion); Anal. calcd. for C_25_H_18_Cl_2_N_6_O_2_ (505.36): C, 59.42; H, 3.59; N, 16.63; Found: C, 59.49; H, 3.62; N, 16.69.

#### 7-(2,4-Dichlorophenyl)-5-(((*E*)-6-(4-hydroxy-3-methoxyphenyl)-4-oxohex-5-en-2-ylidene)amino)-[1,2,4]triazolo[4,3-*a*]pyrimidine-6-carbonitrile (6b)

The reaction mixture was stirred for 21 h, affording a brown crystalline solid in 88% yield; m.p. 248–250 °C; υmax/cm^−1^ (KBr): 3431 (OH), 3069 (CH for aromatic), 2920 (CH for aliphatic), 2244 (CN_Nitrile_), 1669 (C=O_Ketone_), 648 (C–Cl); ^1^H-NMR (400 MHz, DMSO-*d*_*6*_, ppm): δ = 2.55 (s, 3H, CH_3_), 2.73 (s, 2H, CH_2_), 2.89 (s, 3H, OCH_3_), 6.95–6.97 (d, *J* = 8.4 Hz, 2H, Ar–H), 7.30 (s, 1H, Ar–H), 7.39–7.41 (d, *J* = 8.8 Hz, 2H, Ar–H), 7.49 (s, 1H, Ar–H), 7.68–7.72 (d, *J* = 18 Hz, 2H, 2CH), 7.95 (s, 1H, CH_Triazolo_), 9.77 (s, 1H, OH); ^13^C-NMR (100 MHz, DMSO-*d*_*6*_, δ, ppm): 31.25, 34.86, 36.26, 108.59, 109.75, 115.34, 126.54, 129.70, 130.88, 133.08, 135.79, 136.79, 142.20, 144.90, 147.95, 149.70, 153.49, 159.34, 161.79, 162.83, 166.22, 195.50; MS (m/z, %): 521 [M]⁺, 523 [M^2+^]⁺, 525 [M^4+^]⁺; base peak at m/z 160 (100%, fragment ion); Anal. calcd. for C_25_H_18_Cl_2_N_6_O_3_ (521.36): C, 57.59; H, 3.38; N, 16.12; Found: C, 57.65; H, 3.41; N, 16.20.

#### 7-(2,4-Dichlorophenyl)-5-(((*E*)-6-(2,4-dichlorophenyl)-4-oxohex-5-en-2-ylidene)amino)-[1,2,4]triazolo[4,3-*a*]pyrimidine-6-carbonitrile (6c)

The reaction mixture was stirred for 21 h, affording a pale brown solid in 72% yield; m.p. 240–243 °C; υmax/cm^−1^ (KBr): 3088 (CH for aromatic), 2960 (CH for aliphatic), 2240 (CN_Nitrile_), 1692 (C=O_ketone_), 640 (C–Cl); ^1^H-NMR (400 MHz, DMSO-*d*_*6*_, ppm): δ = 2.73 (s, 3H, CH_3_), 2.89 (s, 2H, CH_2_), 6.62–6.66 (d, *J* = 15.2 Hz, 2H, 2CH), 7.45 (s, 1H, Ar–H), 7.52–7.54 (d, *J* = 8.4 Hz, 2H, Ar–H), 7.73–7.76 (d, *J* = 8.8 Hz, 2H, Ar–H), 7.95 (s, 1H, Ar–H), 8.65 (s, 1H, CH _Triazolo_); MS (m/z, %): 544 [M]⁺, 546 [M^2+^]⁺, 548 [M^4+^]⁺; base peak at m/z 264 (100%, fragment ion); Anal. calcd. for C_24_H_14_Cl_4_N_6_O (544.22): C, 52.97; H, 2.59; N, 15.44; Found: C, 52.95; H, 2.60; N, 15.39.

### Density functional theory study

The synthesized compounds were examined from a quantum chemical standpoint and their geometries were optimized using Density Functional Theory (DFT). Initially Avogadro 1.2.0 was used to sketch the compounds and pre-optimize them using MMFF94 force field^49^. Using ORCA 6.0.1 software^50–54^, further optimization was subsequently carried out by using the basis set 6-31G(d,p) and the B3LYP functional which provide a balance between accuracy and computational cost^55,56^, in conjunction with the RIJCOSX approximation to minimize computational cost while maintaining accuracy^52^. The solvent model was used to allow the calculation of different descriptors in a highly polar environment like water, since the major constituent of the biological fluid is water and the drug is predicted to act in such environment. The default solvent model in ORCA 6.0.1 software is Conductor-like Polarizable Continuum Model (CPCM)^57^, hence, it was chosen to carry out the calculation. The HOMO and LUMO energies along with the Energy gap (Eg) were obtained. Additional reactivity descriptors, namely, Ionization Potential (IP), Electron Affinity (Ea), Electronic Chemical Potential (µ), Chemical Hardness (η), Chemical Softness (S), Electrophilicity (ω), Maximum Charge acceptance, and Nucleophilicity (N) were also calculated^42,58–61^.

### Molecular docking simulation

The protein receptor structures (Table 13) were retrieved from the RCSB Protein Data Bank. The three-dimensional structures of proteins were preprocessed with PyMOL. Hydrogen atoms were added, and the structures created were stored in pdbqt format with AutoDock Vina. The ligand structures were energy reduced and translated to mol2 format using Open Babel^62^. Docking simulations were run with AutoDock Vina^63^ which used ligand-centered maps prepared by the AutoGrid software. To validate the reliability of the docking protocol, redocking experiments were performed for each target protein where a co-crystallized native ligand was available (MMP-9, NLRP3, and TNF-α). The native ligand was extracted from the PDB structure, re-docked into the same binding site using identical parameters (AutoDock Vina, grid box cantered on the original ligand coordinates, exhaustiveness = 32), and the resulting pose was compared to the crystallographic conformation. RMSD values were calculated using PyMOL. As summarized in Table 14, all RMSD values were below 2.0 Å (MMP-9: 1.50 Å, NLRP3: 1.23 Å, TNF-α: 1.65 Å), indicating that the docking protocol accurately reproduces the native binding geometry. For IL-6 (1N26), which lacks a co-crystallized inhibitor, the standard drug allopurinol was used as a reference control for comparative binding affinity assessment. Following docking, the resulting protein–ligand complexes were studied. Discovery Studio 4.5 was used to generate two-dimensional interaction diagrams, and ADMETlab 3.0 was used to estimate the compounds’ physicochemical and ADMET attributes^64^. To ensure the reliability of molecular docking and molecular dynamics simulations, the stereochemical quality of the four target protein crystal structures (IL-6: 1N26; TNF-α: 5MU8; MMP-9: 6ESM; NLRP3: 7ALV) was evaluated using Ramachandran plot analysis (Supplementary Fig. 2S). All four structures exhibit overall acceptable stereochemistry, with most residues located in favoured and allowed regions.

**Table 14:** List of target proteins, PDB IDs, active site coordinates, and references.

No	Protein targets	PDB.ID	Resolution	Active site coordinates:			Co-Crystallized Inhibitor	RMSD values (Å)	Common reisdues
				X	Y	Z			
**1**	*IL-6*	1N26	2.40 Å	15.60	55.31	74.2	**–**	–	Ser152 Glu114
**2**	*MMP9*	6ESM	1.10 Å	− 0.333	43.22	14.71	(B9Z)	1.50	His230 Pro193
**3**	*NLRP3*	7ALV	2.83 Å	14.21	31.86	124.6	(RM5)	1.23	Ala228 Arg351
**4**	*TNF*	5MU8	3.00 Å	25.10	20.30	10.32	(JNI)	1.65	Gly24 Glu135

### Molecular dynamics (MD) simulation

Molecular dynamics simulations were performed using GROMACS 2018 with the CHARMM36 force field for protein topology and the CGenFF server for ligand parameter generation. Each protein–ligand complex (free and bound states of IL-6, TNF‑α, MMP‑9, and NLRP3) was placed in a cubic periodic box with a minimum distance of 1.0 nm to the box edges, solvated with TIP3P water molecules, and neutralized with Na^+^/Cl^−^ ions at 0.15 M physiological salt concentration. Energy minimization was conducted using the steepest descent algorithm (50 000 steps, convergence criterion 1000 kJ mol^−1^ nm^−1^). The system was then equilibrated in two phases, each lasting 1000 ps (1 ns): first under NVT conditions (300 K, V‑rescale thermostat, τ = 0.1 ps) with positional restraints (1000 kJ mol^−1^ nm^−2^) on ligand heavy atoms, followed by NPT equilibration (300 K, 1.0 bar, Parrinello–Rahman barostat, τ = 2.0 ps). Production runs were carried out for 50 ns per system under NPT conditions (300 K, 1.0 bar) without restraints, using a leap‑frog integrator with a 2 fs time step. All hydrogen bonds were constrained with the LINCS algorithm, long‑range electrostatics were treated with the Particle Mesh Ewald (PME) method (cut‑off 1.2 nm), and van der Waals interactions used a 1.2 nm cut‑off with a switching function starting at 1.0 nm. Coordinates were saved every 10 ps. Post‑simulation analyses (RMSD, RMSF, radius of gyration, SASA) were performed on the final 40 ns of each trajectory using GROMACS built‑in tools, and MM‑GBSA binding free energies were calculated with the gmx_MMPBSA tool over 1000 equally spaced frames^65^.

## Conclusion

In the present study, a novel series of triazolopyrimidine-based derivatives (5a–d and 6a–c) was successfully synthesized employing nickel (II) oxide nanoparticles (NiO-NPs) as an efficient, recyclable, and environmentally benign heterogeneous nanocatalyst under mild reaction conditions. The target compounds were obtained in good to excellent yields through Knoevenagel condensation and subsequent cyclization processes, and their structures were unambiguously confirmed by comprehensive spectroscopic and analytical techniques. Density functional theory (DFT) calculations revealed that the EE and ZE stereoisomers of compounds 6a, 6b, and 6c represent the most thermodynamically stable configurations, while compounds 5b and 5c displayed relatively smaller HOMO–LUMO energy gaps, suggesting potentially enhanced chemical reactivity among the investigated series.

From a computational standpoint, the in silico molecular docking and molecular dynamics simulation studies indicated that several of the synthesized compounds may interact favorably with key inflammatory mediators implicated in gout pathogenesis, namely IL-6, TNF-α, MMP-9, and NLRP3, exhibiting predicted binding affinities and ligand–protein complex stabilities that merit further investigation. Nevertheless, it must be emphasized that these findings are entirely theoretical in nature and should be interpreted with considerable caution. The ADMET profiling revealed notable pharmacokinetic liabilities, including predicted poor aqueous solubility, low oral bioavailability, and potential risks of cytochrome P450 inhibition and hepatotoxicity, which collectively represent significant challenges that would need to be addressed in any future drug development effort.

Taken together, this work provides preliminary theoretical insights into the structural, electronic, and binding properties of the investigated triazolopyrimidine derivatives and may serve as a foundation for future rational design and optimization studies. However, the authors wish to stress that the present findings do not constitute experimental evidence of biological activity, anti-inflammatory efficacy, or clinical relevance. Rigorous in vitro and in vivo biological evaluations are indispensable before any meaningful conclusions regarding the therapeutic potential of these compounds can be drawn. It is hoped that this computational framework will guide and inspire subsequent experimental investigations aimed at identifying novel, safe, and effective candidates for the management of gout and related inflammatory conditions.

## Supplementary Information

Supplementary Information.

## Data Availability

All data generated or analyzed during this study are included in this published article [and its supplementary information files].

## References

[1] Dalbeth, N. *et al.* Gout (primer). 5 (2019).

[2] Kuo, C.-F., Grainge, M. J., Zhang, W. & Doherty, M. Global epidemiology of gout: prevalence, incidence and risk factors. *Nat. Rev. Rheumatol.* **11**, 649–662 (2015). 26150127 10.1038/nrrheum.2015.91

[3] Martinon, F. et al. Gout-associated uric acid crystals activate the NALP3 inflammasome. *Nature* **440**, 237–241 (2006). 16407889 10.1038/nature04516

[4] Ossipova, E. et al. Affinity purified anti-citrullinated protein/peptide antibodies target antigens expressed in the rheumatoid joint. *Arthritis Res. Ther.* **16**, R167 (2014). 25112157 10.1186/ar4683 PMC4448322

[5] Tang, X. et al. Indicator regularized non-negative matrix factorization method-based drug repurposing for COVID-19. *Front. Immunol.* **11**, 603615 (2021). 33584672 10.3389/fimmu.2020.603615 PMC7878370

[6] Dalbeth, N. et al. Hyperuricaemia and gout: time for a new staging system?. *Ann. Rheum. Dis.* **73**, 1598–1600 (2014). 24718961 10.1136/annrheumdis-2014-205304

[7] Lang, J. et al. LC-MS-based metabolomics reveals the mechanism of anti-gouty arthritis effect of Wuwei Shexiang pill. *Front. Pharmacol.* **14**, 1213602 (2023). 37637422 10.3389/fphar.2023.1213602 PMC10450745

[8] White, W. B. et al. Cardiovascular safety of febuxostat or allopurinol in patients with gout. *N. Engl. J. Med.* **378**, 1200–1210 (2018). 29527974 10.1056/NEJMoa1710895

[9] Altabás-González, I. et al. Does expert opinion match the definition of lupus low disease activity state? Prospective analysis of 500 patients from a Spanish multicentre cohort. *Rheumatology* **62**, 1162–1169 (2023). 35961050 10.1093/rheumatology/keac462 PMC9977118

[10] Akuthota, P., *et al*. vol. 10, 2118 (Frontiers Media SA, 2019).

[11] Feng, C. et al. Precisely tailoring molecular structure of doxorubicin prodrugs to enable stable nanoassembly, rapid activation, and potent antitumor effect. *Pharmaceutics* **16**, 1582 (2024). 39771561 10.3390/pharmaceutics16121582 PMC11679313

[12] Huang, X.-C. et al. Design, synthesis and in vitro evaluation of novel dehydroabietic acid derivatives containing a dipeptide moiety as potential anticancer agents. *Eur. J. Med. Chem.* **89**, 370–385 (2015). 25462253 10.1016/j.ejmech.2014.10.060

[13] Endo, K. et al. 8-Substituted 2-alkynyl-N9-propargyladenines as A2A adenosine receptor antagonists. *Bioorg. Med. Chem.* **22**, 3072–3082 (2014). 24815000 10.1016/j.bmc.2014.04.041

[14] Marzouk, M. A. et al. Dual α-amylase and α-glucosidase inhibition by 1, 2, 4-triazole derivatives for diabetes treatment. *Sci. Rep.* **15**, 27172 (2025). 40715234 10.1038/s41598-025-11214-4 PMC12297423

[15] Shehab, W. *et al*. Design, synthesis, and computational studies as cytotoxicity of novel pyrimidine carbonitrile derivatives as dual-target inhibitors of BRD4. *Bull. Faculty Sci.* 141–152 (2025).

[16] Jimenez, C. et al. Exploring the size adaptability of the B ring binding zone of the colchicine site of tubulin with para-nitrogen substituted isocombretastatins. *Eur. J. Med. Chem.* **100**, 210–222 (2015). 26092446 10.1016/j.ejmech.2015.05.047

[17] Nowakowska, Z. et al. A review of anti-infective and anti-inflammatory chalcones. *Eur. J. Med. Chem.* **42**, 125–137 (2007). 17112640 10.1016/j.ejmech.2006.09.019

[18] Shehab, W. et al. Computational chemistry for some novel pyrimidine derivatives as significant antioxidants using cytochrome c peroxidase enzyme. *Bull. Faculty Sci.* **2025**, 171–177 (2025).

[19] Tantawy, E. et al. Eco-friendly synthesis, docking study, pharmacokinetics studies, and anti-proliferative evaluation of pyrimidine derivatives as dual Topoisomerase II and HSP90 inhibitors. *Egypt. J. Chem.* **68**, 203–218 (2025).

[20] Polshettiwar, V. et al. Green chemistry by nano-catalysis. *Green Chem.* **12**, 743–754 (2010).

[21] Yang, H. et al. Preparation and catalytic performance of Ag, Au, Pd or Pt nanoparticles supported on 3DOM CeO2–Al2O3 for toluene oxidation. *J. Mol. Catal. A Chem.* **414**, 9–18 (2016).

[22] Jawhari, A. H. et al. Design, synthesis, in silico ADMET prediction, molecular docking, antimicrobial and antioxidant evaluation of novel diethyl pyridinyl phosphonate derivatives. *Curr. Org. Chem.* **27**, 860–875 (2023).

[23] Khidre, R. E. et al. Design, one-pot synthesis, in silico ADMET prediction and molecular docking of novel triazolyl thiadiazine and thiazole derivatives with evaluation of antimicrobial, antioxidant and antibiofilm inhibition activity. *J. Iran. Chem. Soc.* **20**, 2923–2947 (2023).

[24] Zhou, R. et al. NEDD: A network embedding based method for predicting drug-disease associations. *BMC Bioinform.* **21**, 387 (2020). 10.1186/s12859-020-03682-4 PMC7495830 32938396

[25] Wang, S. et al. Flat-Lattice-CNN: A model for Chinese medical-named-entity recognition. *PLoS ONE* **20**, e0331464 (2025). 40966234 10.1371/journal.pone.0331464 PMC12445539

[26] Rezvani, M. A. et al. Deep oxidative desulfurization of gas oil by iron (III)-substituted polyoxometalate immobilized on nickel (II) oxide,((n-C4H9) 4N) 4H [PW11FeO39]@ NiO, as an efficient nanocatalyst. *Sci. Rep.* **13**, 15233 (2023). 37709938 10.1038/s41598-023-42545-9 PMC10502112

[27] El Komy, G. et al. Innovative synthesis of nickel nanoparticles in polystyrene matrix with enhanced optical and magnetic properties. *J. Inorg. Organomet. Polym Mater.* **29**, 1983–1994 (2019).

[28] Anand, G. T. et al. Structural and optical properties of nickel oxide nanoparticles: Investigation of antimicrobial applications. *Surf. Interfaces* **18**, 100460 (2020).

[29] Olajire, A. et al. Green synthesis of nickel oxide nanoparticles and studies of their photocatalytic activity in degradation of polyethylene films. *Adv. Powder Technol.* **31**, 211–218 (2020).

[30] Mabrouk, M. et al. Bioactivity and cell viability of Ag+-and Zr 4+-co-doped biphasic calcium phosphate. *Appl. Phys. A* **127**, 1–19 (2021).

[31] Sagadevan, S. et al. Investigations on structural, optical, morphological and electrical properties of nickel oxide nanoparticles. *Int. J. Nanopart.* **8**, 289–301 (2015).

[32] Firisa, S. G. et al. Synthesis of nickel oxide nanoparticles and copper-doped nickel oxide nanocomposites using phytolacca dodecandra l’herit leaf extract and evaluation of its antioxidant and photocatalytic activities. *ACS Omega* **7**, 44720–44732 (2022). 36530241 10.1021/acsomega.2c04042 PMC9753499

[33] Jayaseelan, C. et al. Effect of sub-acute exposure to nickel nanoparticles on oxidative stress and histopathological changes in *Mozambique tilapia*, *Oreochromis mossambicus*. *Ecotoxicol. Environ. Saf.* **107**, 220–228 (2014). 25011118 10.1016/j.ecoenv.2014.06.012

[34] Panja, S. K. et al. First report of the application of simple molecular complexes as organo-catalysts for Knoevenagel condensation. *RSC Adv.* **5**, 65526–65531 (2015).

[35] Ablajan, K. et al. An efficient three component one-pot synthesis of 5-amino-7-aryl-7, 8-dihydro-[1, 2, 4] triazolo [4, 3-a]-pyrimidine-6-carbonitriles. *Molecules* **17**, 1860–1869 (2012). 22334064 10.3390/molecules17021860 PMC6268426

[36] Aider, N. et al. Studies of the solvent-free knoevenagel condensation over commercial NiO compared with NiO drived from hydrotalcites. *Bull. Chem. React. Eng. Catal.* **18**, 186–199 (2023).

[37] Roy, A. et al. Synthesis of nitrogen and oxygen containing heterocyclic compounds using nano catalyst: A review. *J. Turk. Chem. Soc. Sect. A Chem.* **8**, 851–862 (2021).

[38] Pan, Y. Magnetic nanocatalyst for microwave-assisted synthesis of Benzo [4, 5] imidazo [1, 2-a] pyrimidines via A3 coupling. *Front. Chem.* **13**, 1631183 (2025). 40727249 10.3389/fchem.2025.1631183 PMC12302415

[39] Devi, S. et al. Evaluation of substituent effect in Z-isomer stability of arylazo-1H-3,5-dimethylpyrazoles: Interplay of steric, electronic effects and hydrogen bonding. *J. Org. Chem.* **83**, 4307–4322. 10.1021/acs.joc.7b02604 (2018). 29565133 10.1021/acs.joc.7b02604

[40] Dąbrowa, K. et al. Anion-tunable control of thermal Z→E isomerisation in basic azobenzene receptors. *Chem. Commun.* **50**, 15748–15751. 10.1039/C4CC07798A (2014). 10.1039/c4cc07798a 25369943

[41] Sundharaj, V., *et al.*. Microwave assisted palladium catalyzed carbon-carbon bond formation to synthesise novel benzimidazole derivatives and their Photophysical properties, molecular docking, and DFT study. *Heliyon*. 10.1016/j.heliyon.2025.e42105 (2025). 10.1016/j.heliyon.2025.e42105 PMC11808504 39931475

[42] Domingo, L. R. et al. Applications of the conceptual density functional theory indices to organic chemistry reactivity. *Molecules* **21**, 748 (2016). 27294896 10.3390/molecules21060748 PMC6273244

[43] Liu, C., *et al*. Effects of baicalin on gout based on network pharmacology, molecular docking, and in vitro experiments. *J. Inflamm. Res.* 1543–1556 (2025). 10.2147/JIR.S480911 PMC11806711 39925939

[44] Geng, Y.-H. et al. Potential molecular mechanisms of Ermiao san in the treatment of hyperuricemia and gout based on network pharmacology with molecular docking. *Medicine* **101**, e30525 (2022). 36123941 10.1097/MD.0000000000030525 PMC9478232

[45] Nabil, M. *et al.* Anti-diabetic potential of *Chamaerops humilis* L. aerial parts: Phenolic compounds with α-amylase and α-glucosidase inhibitory activates in-vitro, in-vivo and in-silico studies. *J. Mol. Struct.* **1312**, 138550 (2024).

[46] Cao, J.-F. et al. Exploring the mechanism of action of dapansutrile in the treatment of gouty arthritis based on molecular docking and molecular dynamics. *Front. Physiol.* **13**, 990469 (2022). 36105284 10.3389/fphys.2022.990469 PMC9465377

[47] Ahmed, N. S. et al. Design, synthesis, anticancer activity, and in silico computational studies of new imidazolone-based derivatives with potential multi-target kinase inhibitory activity. *Bioorg. Med. Chem.* **129**, 118292 (2025). 40609134 10.1016/j.bmc.2025.118292

[48] Farag, B. *et al.* Novel pyrimidinone scaffolds synthesized via nanotechnology as promising inhibitors of the hepatitis a virus with computational analyses. *Bioorg. Chem.* 109258 (2025). 10.1016/j.bioorg.2025.109258 41275768

[49] Hanwell, M. D. et al. Avogadro: An advanced semantic chemical editor, visualization, and analysis platform. *J. Cheminform.* **4**, 17. 10.1186/1758-2946-4-17 (2012). 22889332 10.1186/1758-2946-4-17 PMC3542060

[50] Neese, F. Software update: The ORCA program system—Version 5.0. *WIREs Comput. Mol. Sci.* **12**, e1606, 10.1002/wcms.1606 (2022).

[51] Neese, F. An improvement of the resolution of the identity approximation for the formation of the Coulomb matrix. *J. Comput. Chem.* **24**, 1740–1747. 10.1002/jcc.10318 (2003). 12964192 10.1002/jcc.10318

[52] Neese, F., *et al*. Efficient, approximate and parallel Hartree–Fock and hybrid DFT calculations. A ‘chain-of-spheres’ algorithm for the Hartree–Fock exchange. *Chem. Phys.* **356**, 98–109. 10.1016/j.chemphys.2008.10.036 (2009).

[53] Helmich-Paris, B. et al. An improved chain of spheres for exchange algorithm. *J. Chem. Phys.* **155**, 104109. 10.1063/5.0058766 (2021). 34525816 10.1063/5.0058766

[54] Neese, F. The SHARK integral generation and digestion system. *J. Comput. Chem.* **44**, 381–396. 10.1002/jcc.26942 (2023). 35678278 10.1002/jcc.26942

[55] Becke, A. D. Density-functional thermochemistry. III. The role of exact exchange. *J. Chem. Phys.* **98**, 5648–5652. 10.1063/1.464913 (1993).

[56] Sousa, S. F. et al. General performance of density functionals. *J. Phys. Chem. A* **111**, 10439–10452. 10.1021/jp0734474 (2007). 17718548 10.1021/jp0734474

[57] Barone, V. et al. Quantum calculation of molecular energies and energy gradients in solution by a conductor solvent model. *J. Phys. Chem. A* **102**, 1995–2001. 10.1021/jp9716997 (1998).

[58] Mahmoudi, S. et al. Density functional theory studies of the antioxidants—A review. *J. Mol. Model.* **27**, 271. 10.1007/s00894-021-04891-1 (2021). 34463834 10.1007/s00894-021-04891-1

[59] Khaoua, O. Reactivity, bioactivity, and antileishmanial activity of dihydrosyrindine and syringine: Modelling, cytotoxicity, molecular docking, molecular dynamics, and MM-GBSA analyses. *J. Mol. Graph. Model.* **142**, 109183. 10.1016/j.jmgm.2025.109183 (2026). 41076844 10.1016/j.jmgm.2025.109183

[60] Khaoua, O. et al. In vitro antibacterial, antifungal activities, reactivity, bioactivity, GUSAR, cytotoxicity profiles, molecular docking, and dynamic simulations of quinoline acrylonitrile derivatives. *ChemistrySelect* **10**, e03272. 10.1002/slct.202503272 (2025).

[61] Khaoua, O. et al. Synthesis, in vitro antimicrobial activity, theoretical DFT-based reactivity investigations, NCI-RDG, NLO, EFL, LOL, AIM analyses, molecular docking, and dynamic simulations of novel 2-(Hydroxy(tetrazolo[1,5-a]quinolin-4-yl)methyl)acrylonitrile derivatives. *J. Mol. Struct.* **1349**, 143849. 10.1016/j.molstruc.2025.143849 (2026).

[62] O’Boyle, N. M. et al. *Open Babel: An open chemical toolbox.* **3**, 33 (2011). 10.1186/1758-2946-3-33 PMC3198950 21982300

[63] Eberhardt, J., *et al*. AutoDock Vina 1.2. 0: new docking methods, expanded force field, and python bindings. *J. Chem. Inf. Model.* **61**, 3891–3898 (2021). 10.1021/acs.jcim.1c00203 PMC10683950 34278794

[64] Fu, L. *et al.* ADMETlab 3.0: an updated comprehensive online ADMET prediction platform enhanced with broader coverage, improved performance, API functionality and decision support. *Nucleic Acids Res.* **52**, W422–W431 (2024). 10.1093/nar/gkae236 PMC11223840 38572755

[65] Páll, S. *et al.* Heterogeneous parallelization and acceleration of molecular dynamics simulations in GROMACS. *J. Chem. Phys.* **153** (2020). 10.1063/5.0018516 33032406

