# Supplementary material for: Green NiO nanoparticle catalyzed synthesis of novel triazolopyrimidine derivatives with physicochemical characterization and computational evaluation for gout therapy
Source: Sci Rep. 2026 Jun 26;16:19609. doi: 10.1038/s41598-026-57449-7 (PMC13304161; doi:10.1038/s41598-026-57449-7)
Supplement: Supplementary file 1 — Supplementary Information. [file 41598_2026_57449_MOESM1_ESM.pdf]

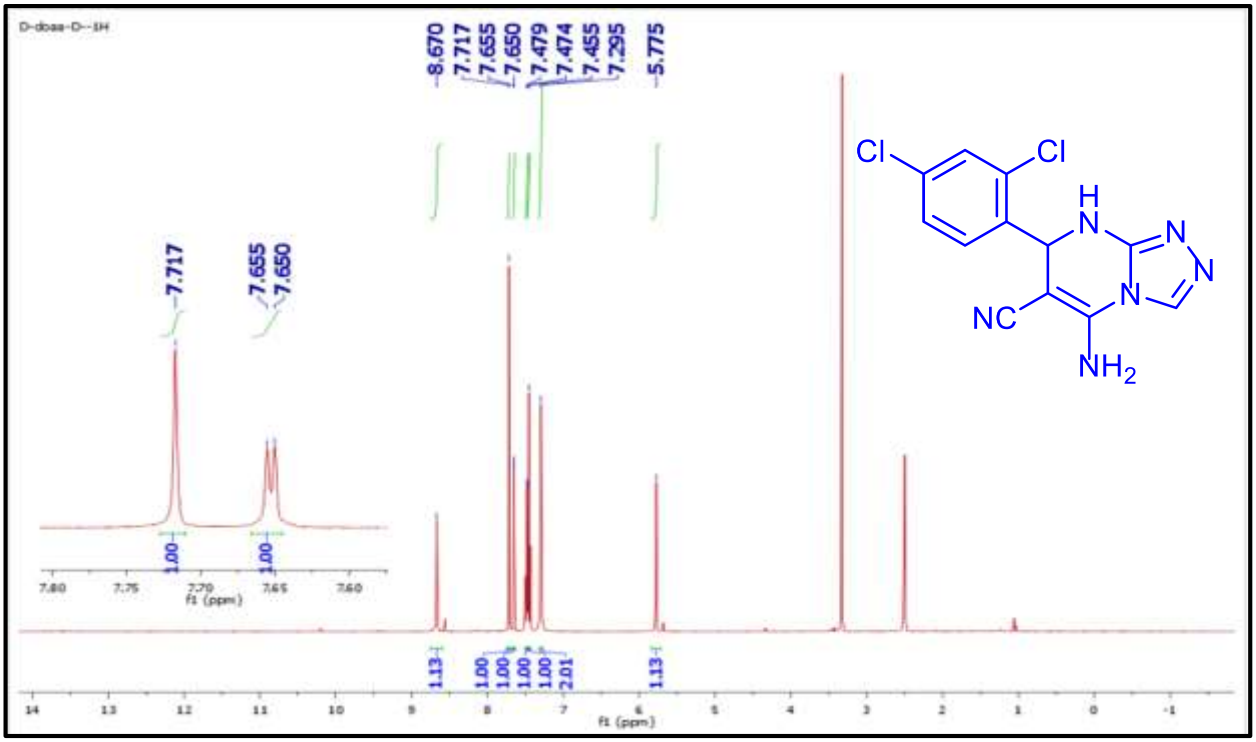

^{1}H-NMR Spectrum of compound 2

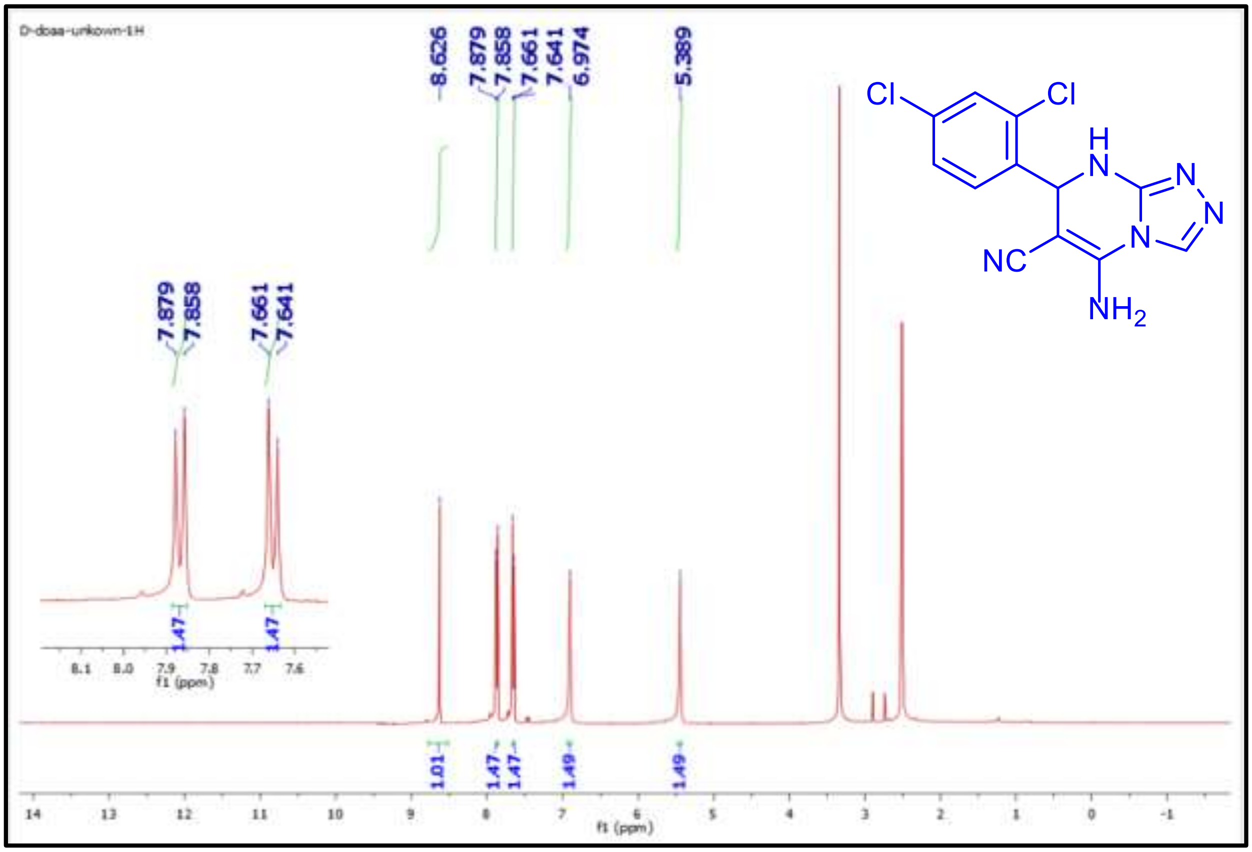

D₂O-NMR Spectrum of compound 2

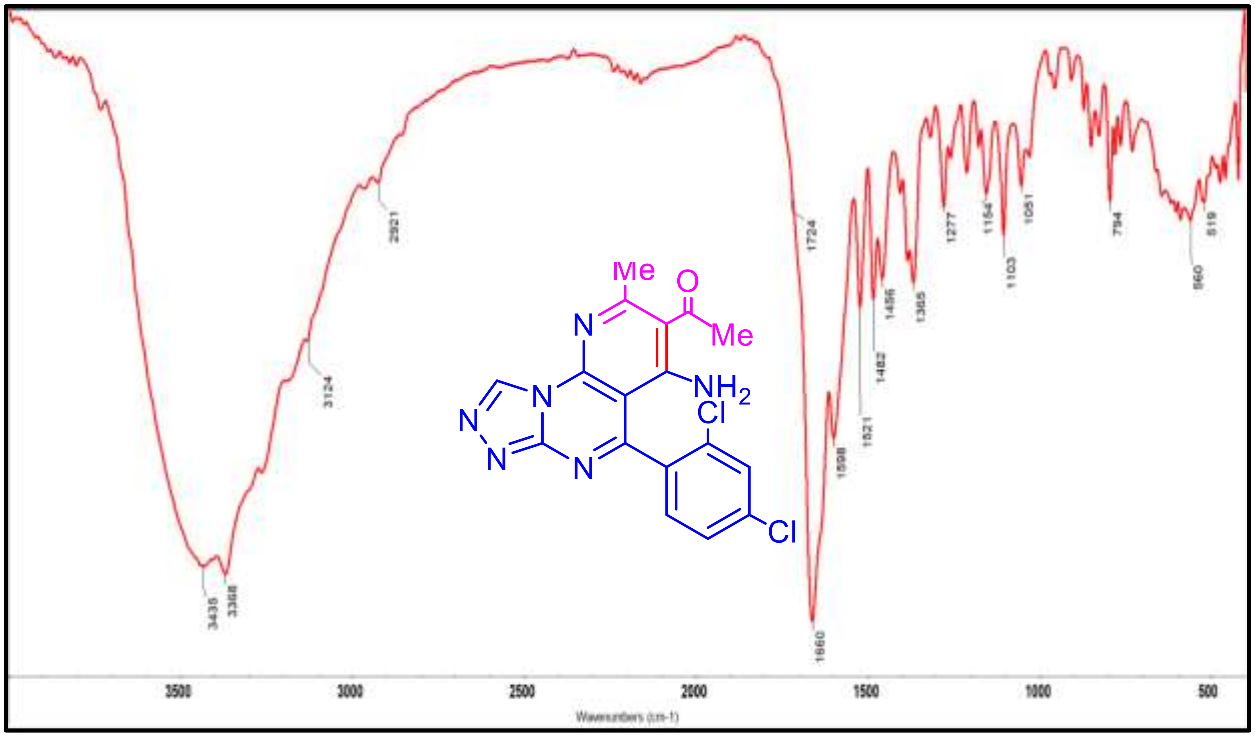

IR Spectrum of compound 3

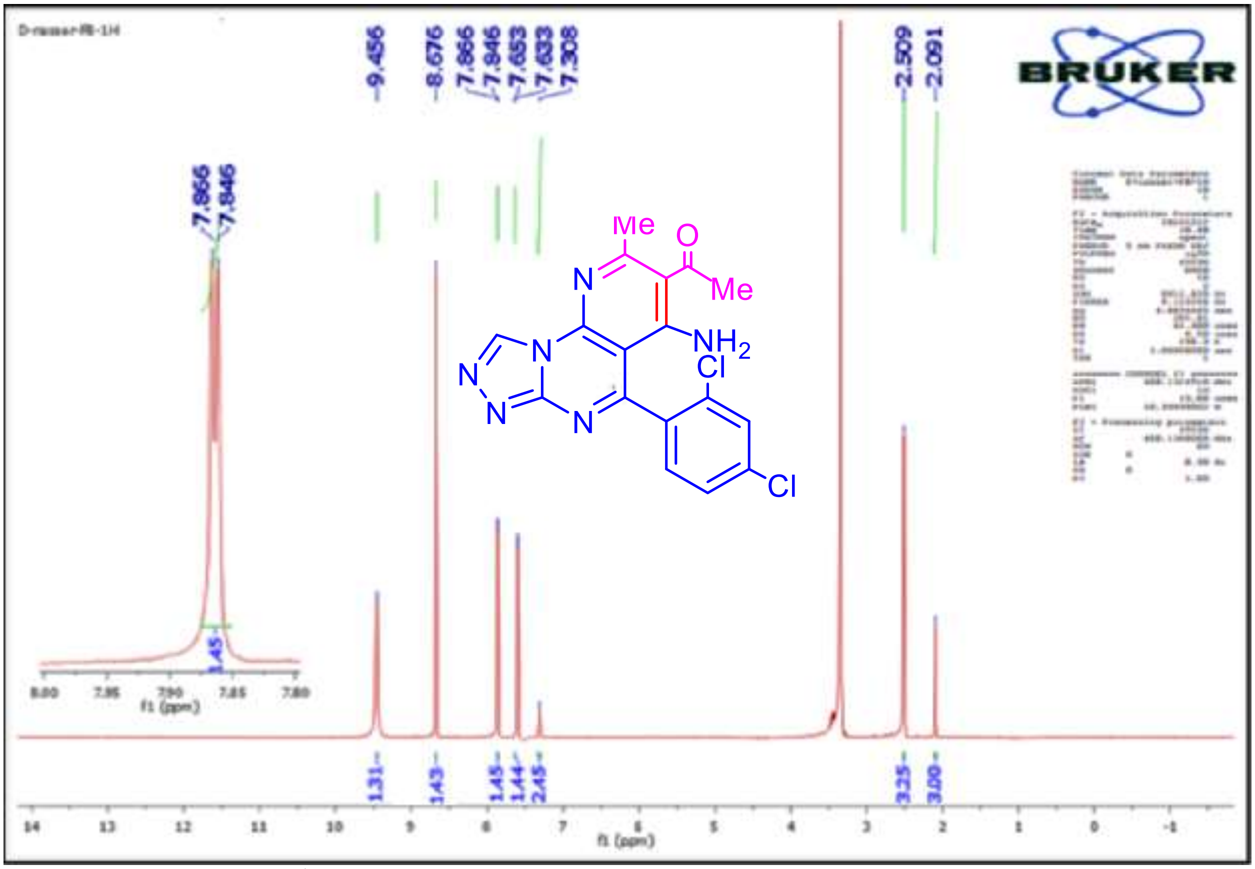

^{1}H-NMR Spectrum of compound 3 by DMSO-d_6

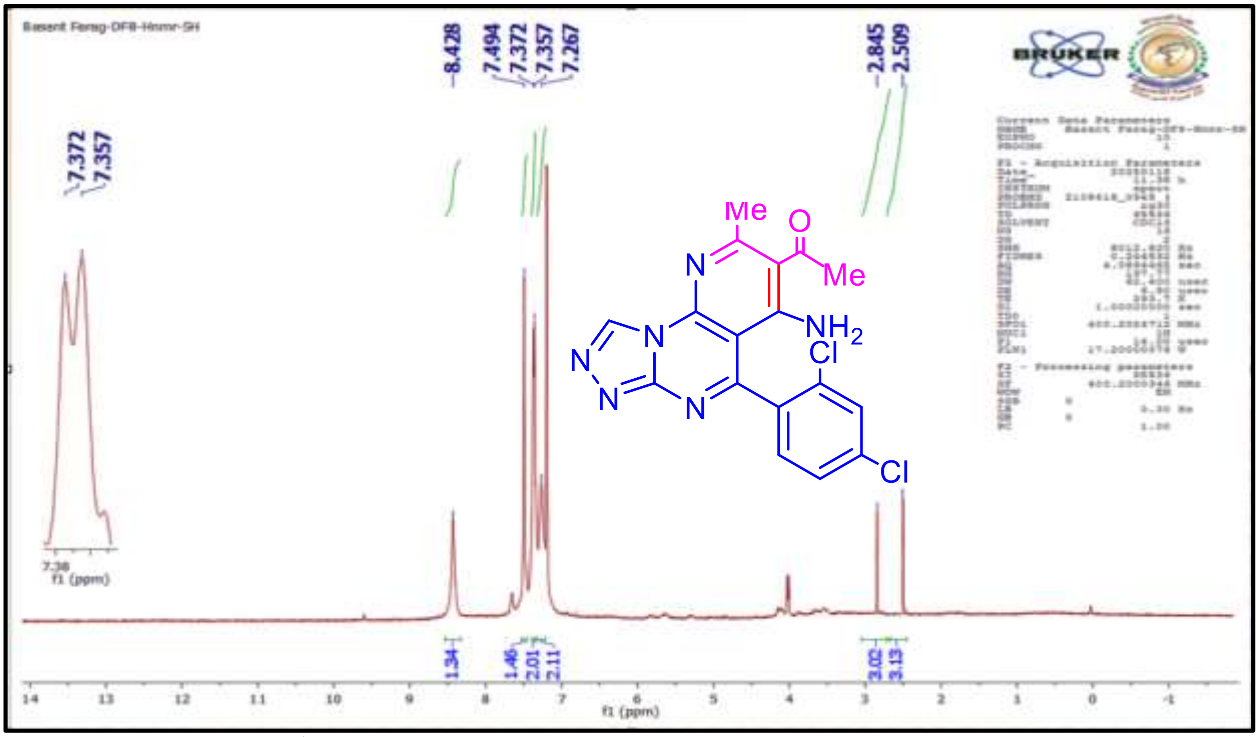

^{1}H-NMR Spectrum of compound 3 by CD_3Cl_3-d_3

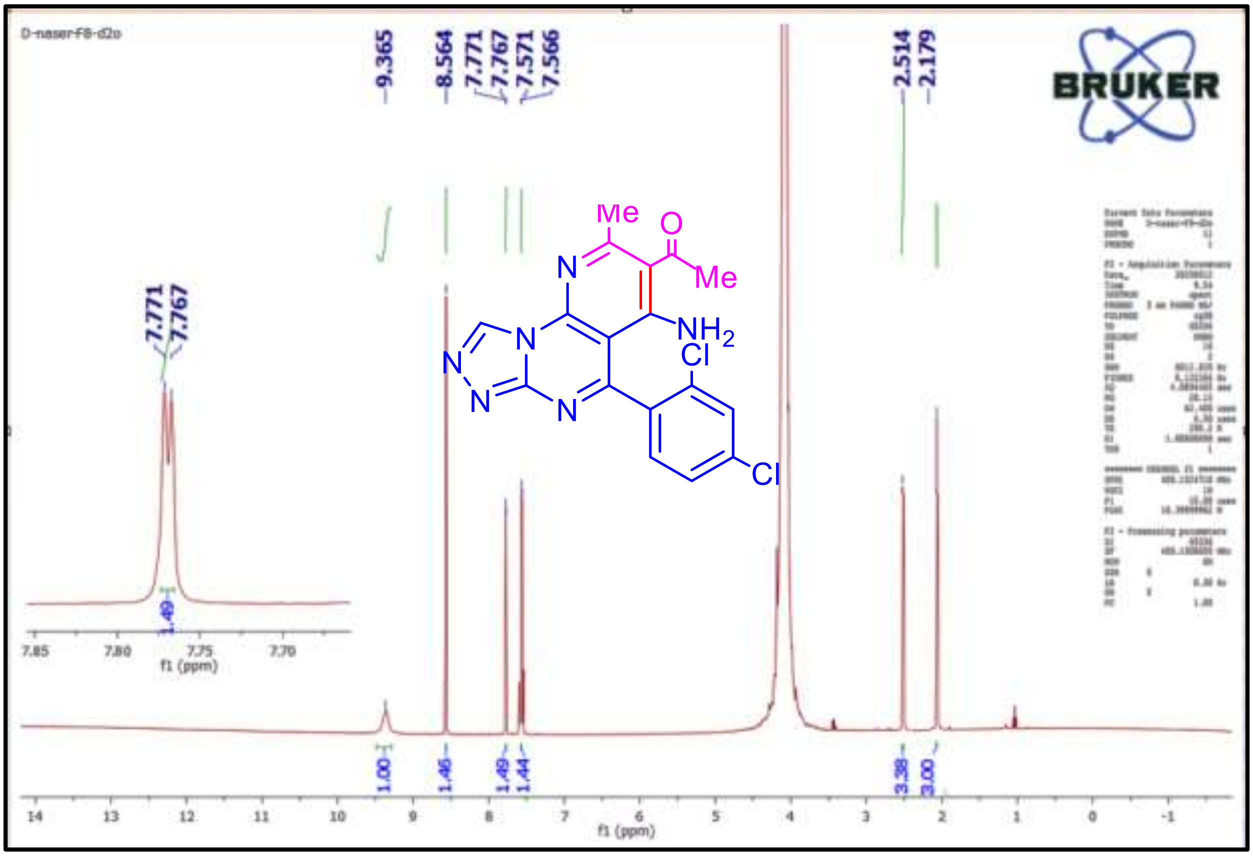

D_2O-NMR Spectrum of compound 3 by DMSO-d_6

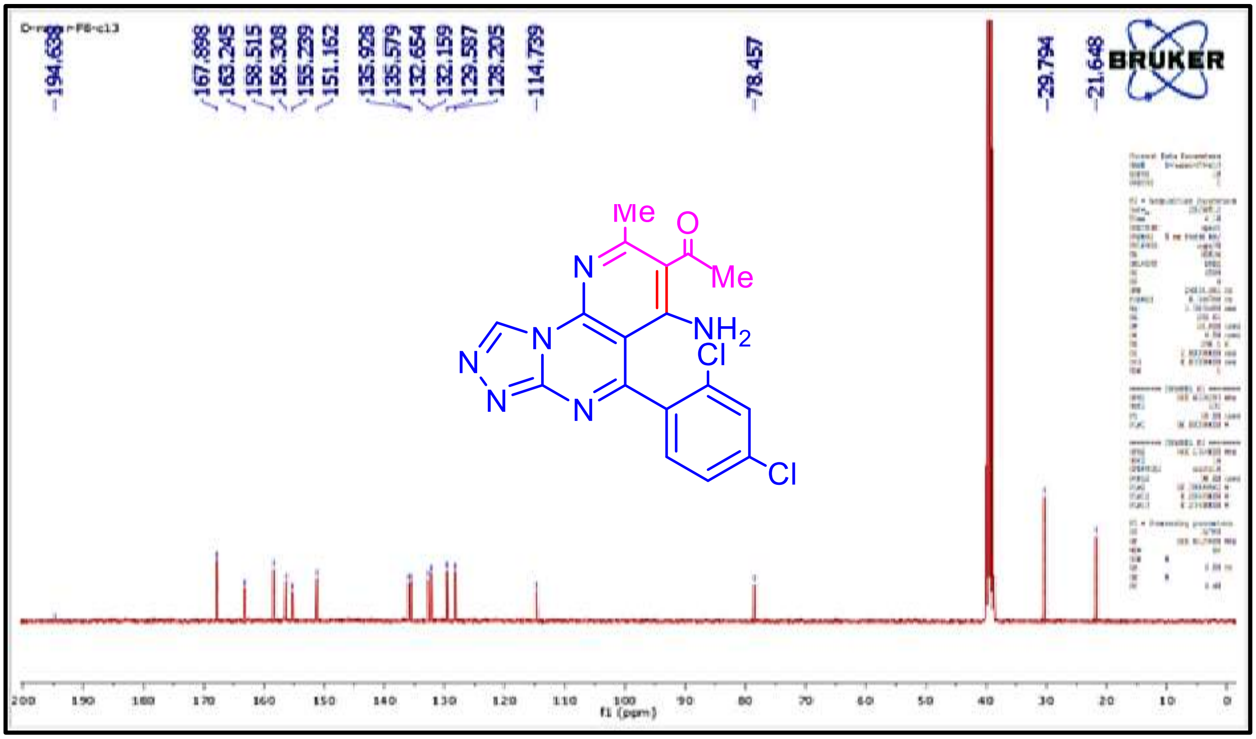

^{13}**C-NMR Spectrum of compound 3 by DMSO-*d_6***

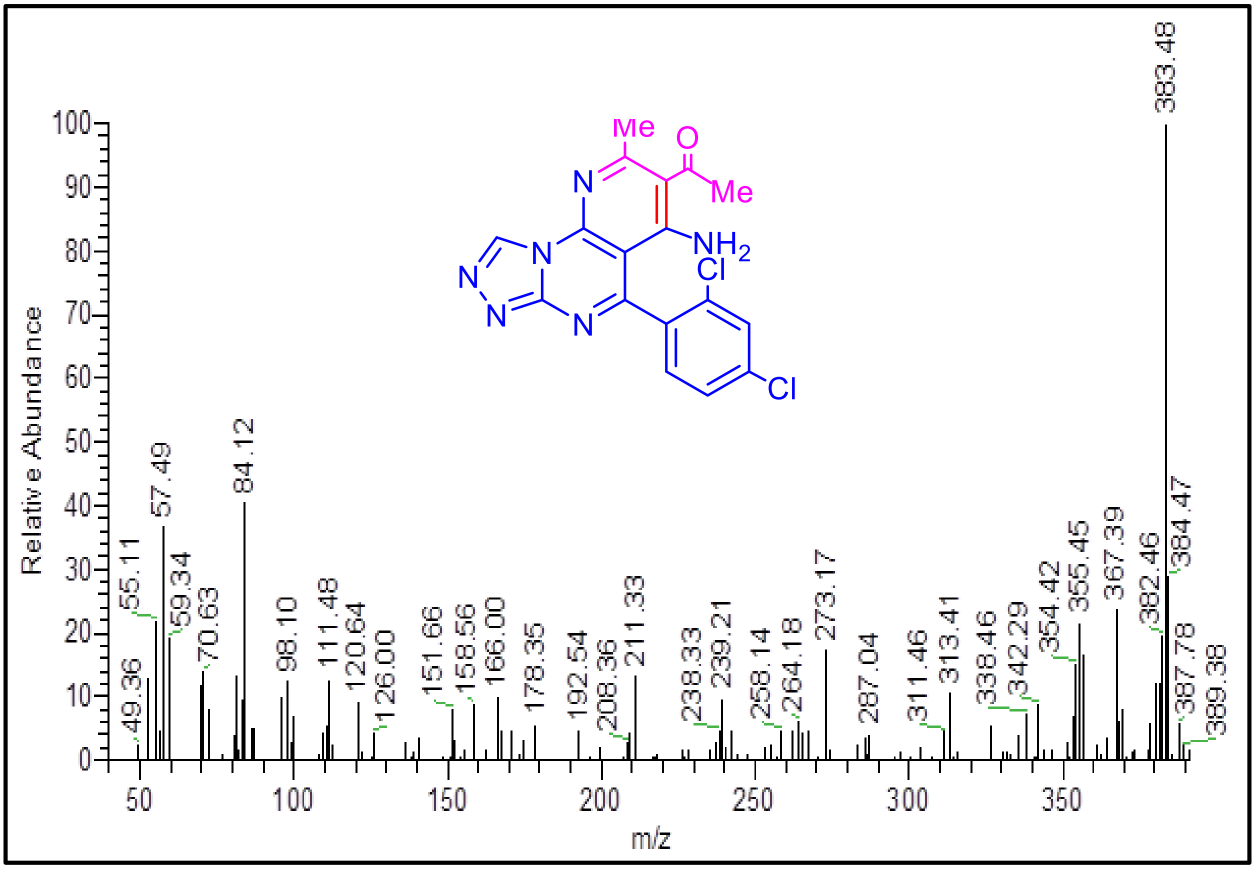

Mass spectrum of compound 3

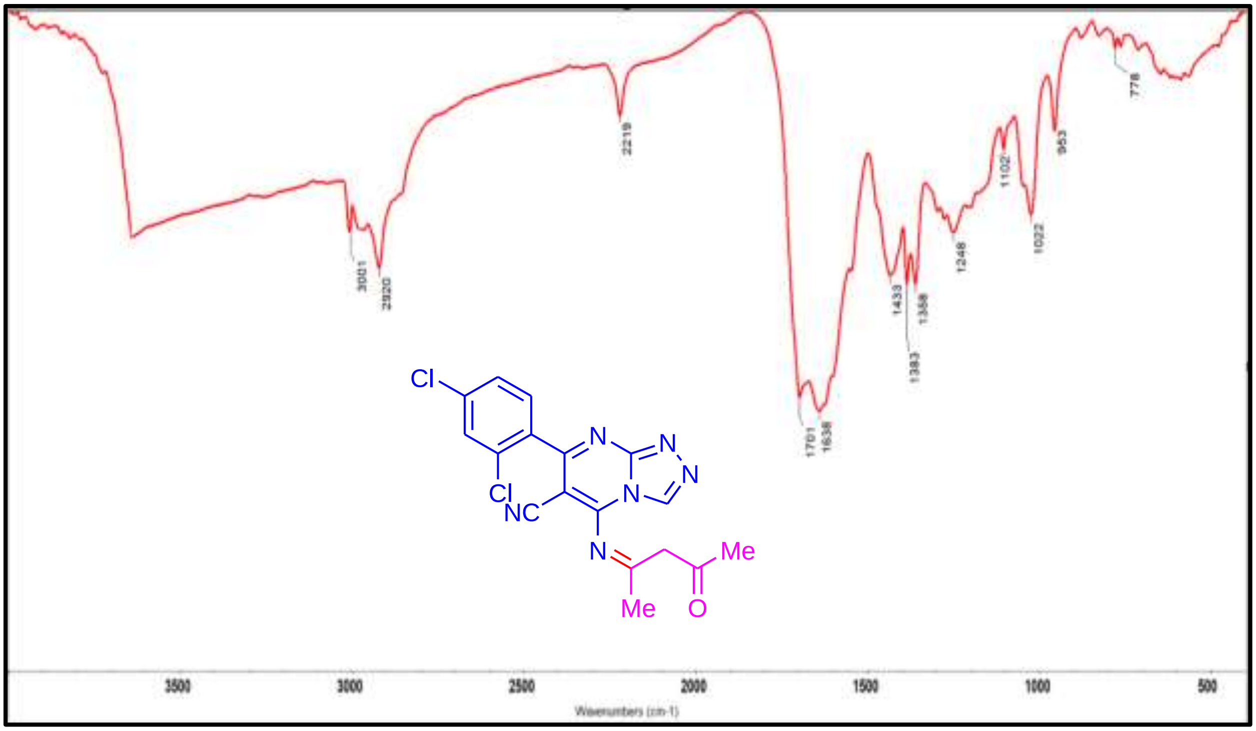

IR Spectrum of compound 4

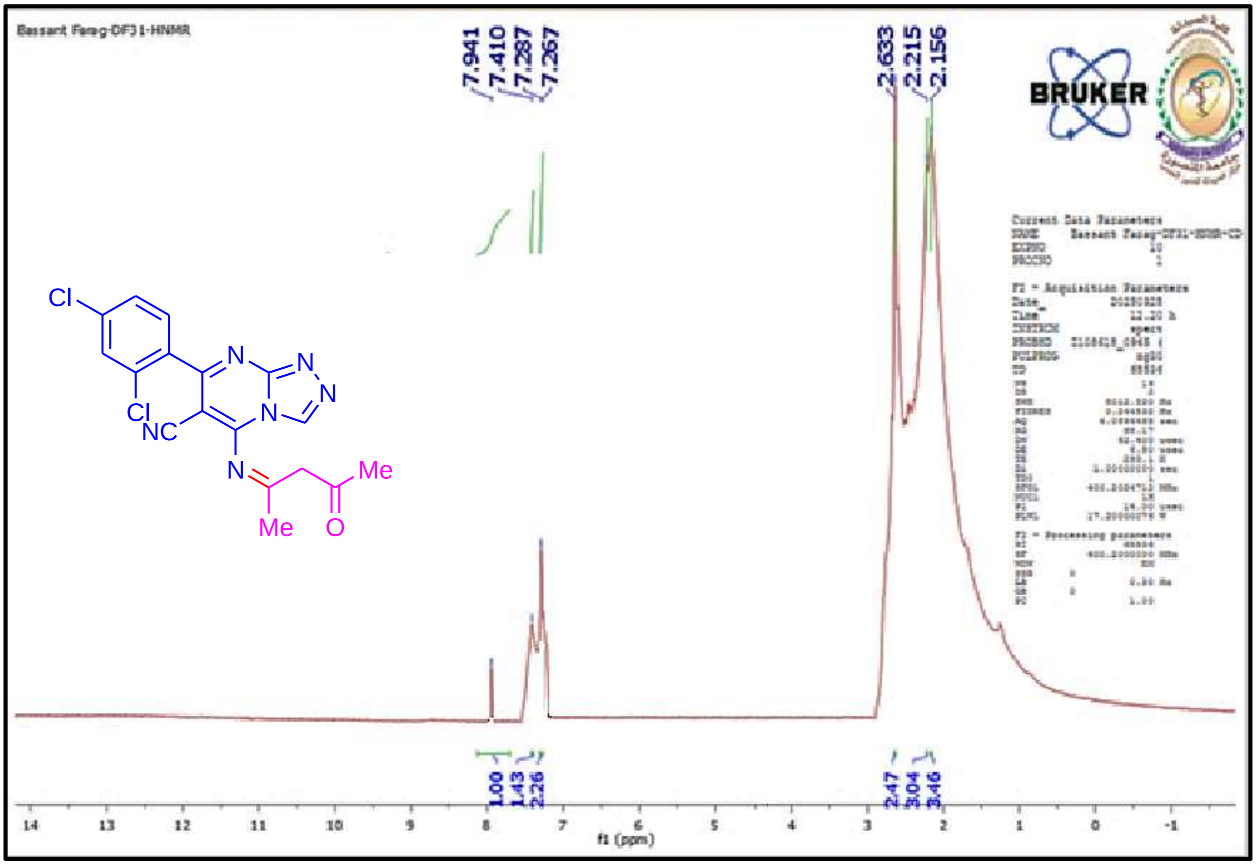

^{1}H-NMR Spectrum of compound 4

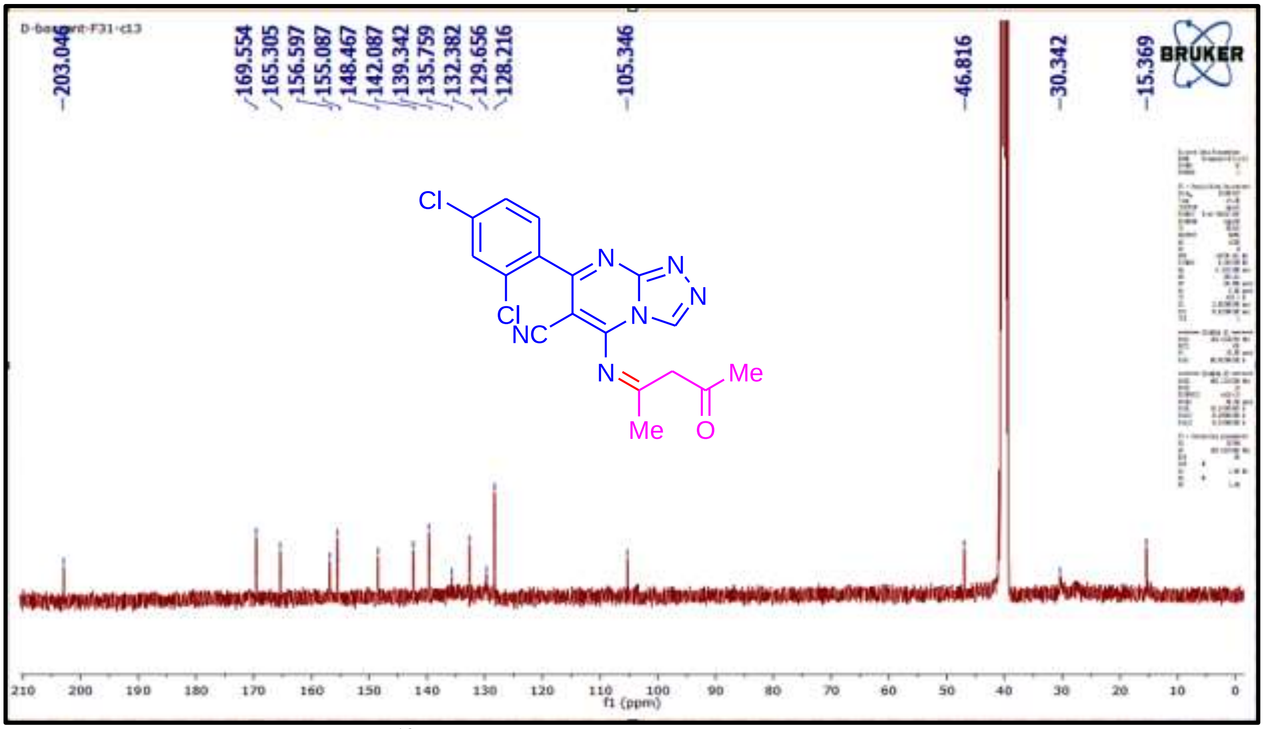

^{13}C-NMR Spectrum of compound 4

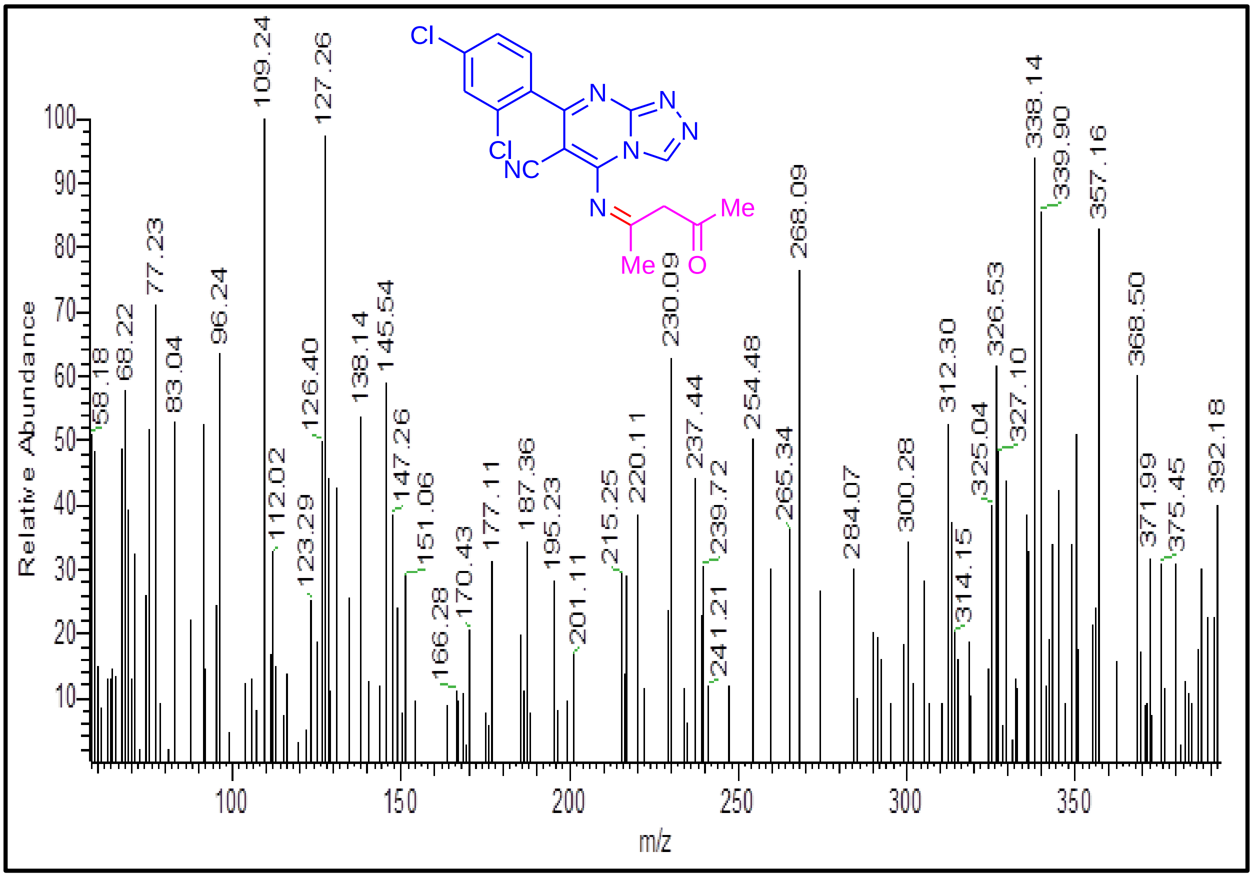

Mass spectrum of compound 4

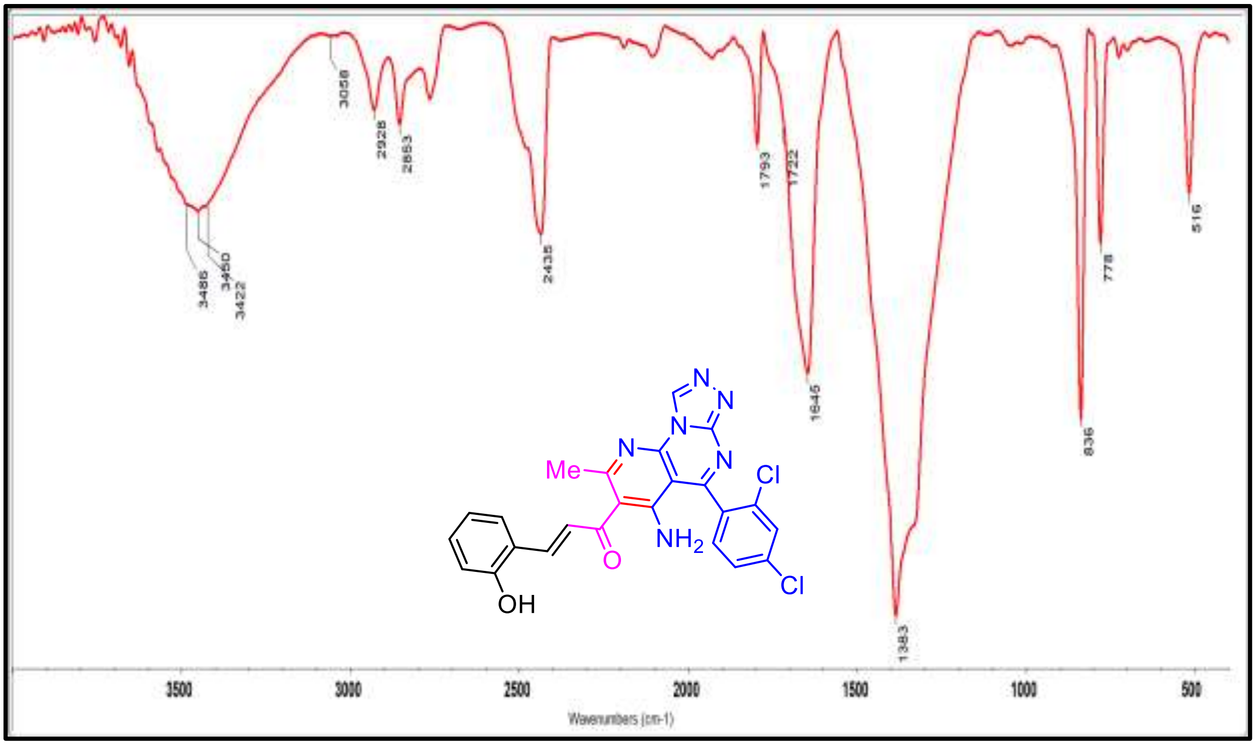

IR Spectrum of compound 5a

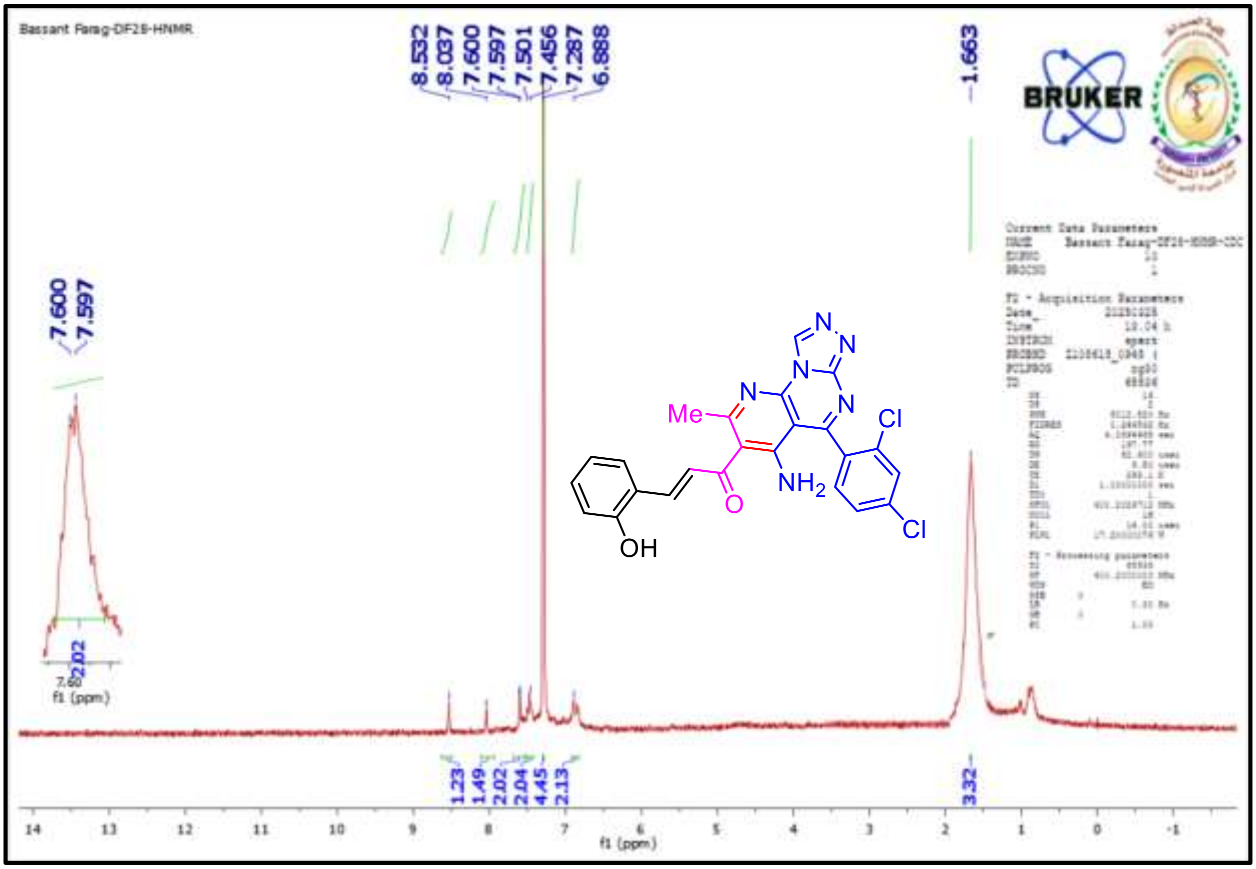

^{1}H-NMR Spectrum of compound 5a

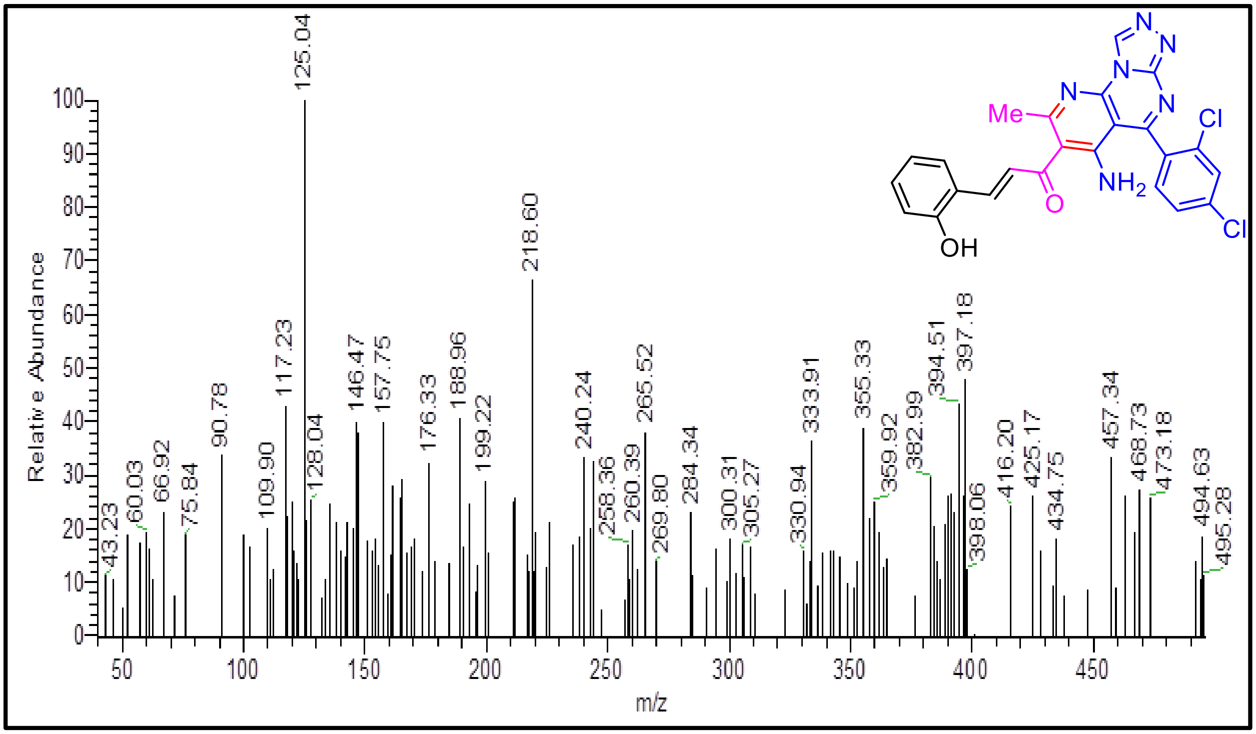

Mass spectrum of compound 5a

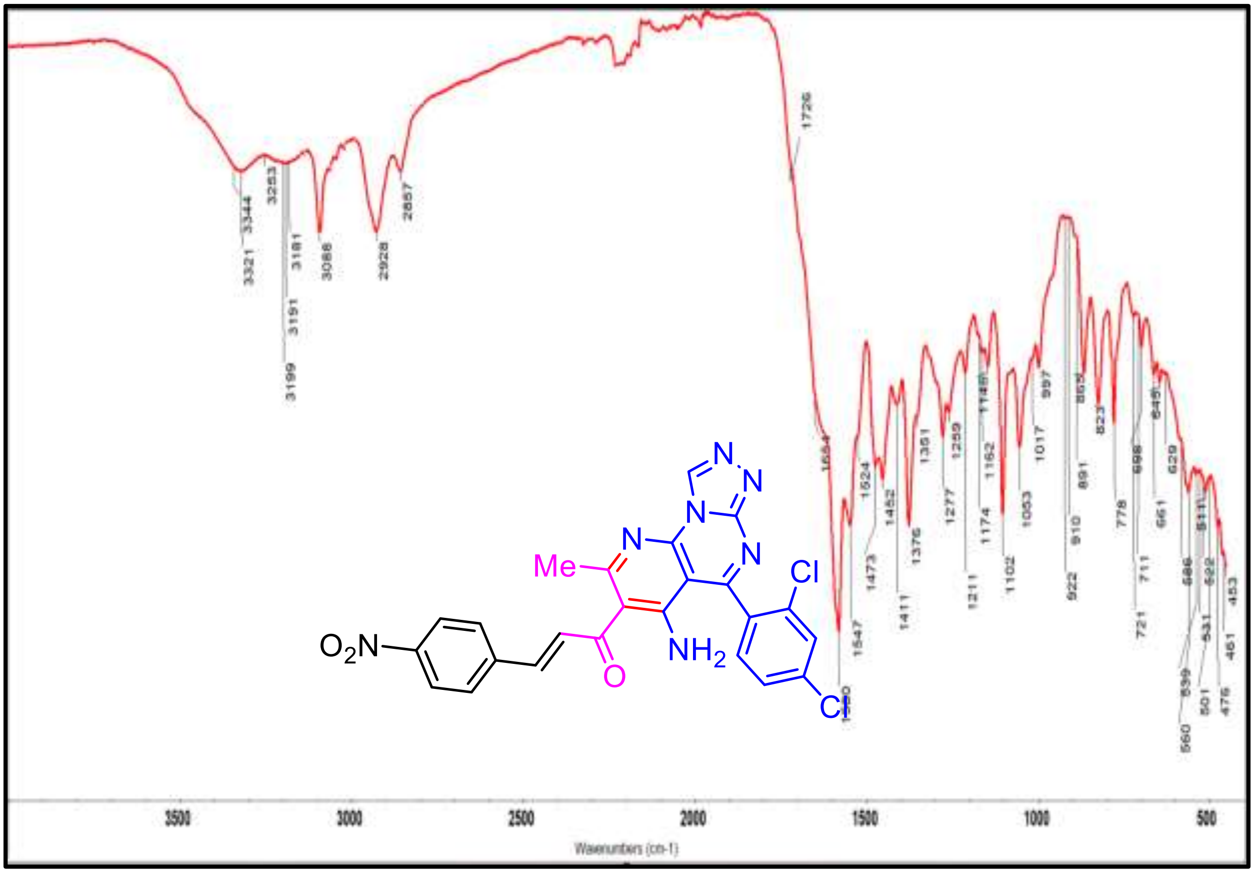

IR Spectrum of compound 5b

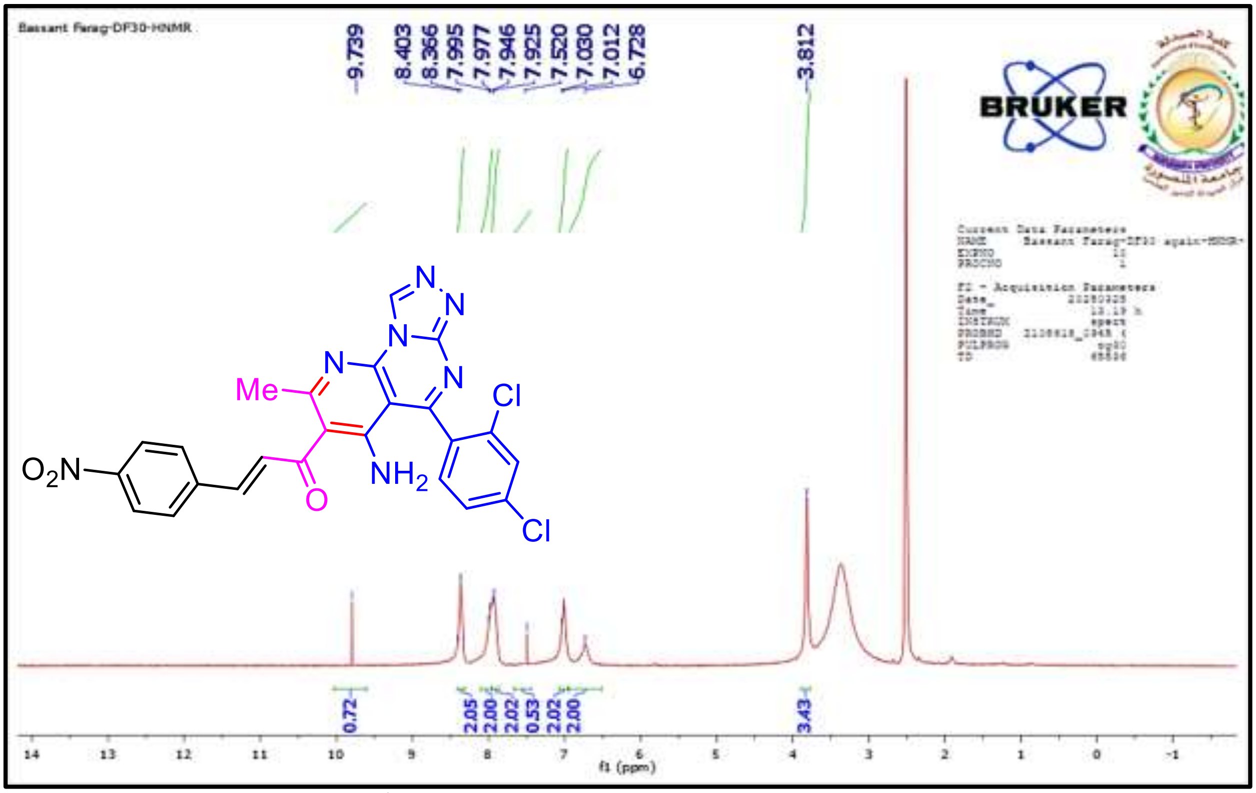

^{1}H-NMR Spectrum of compound 5b

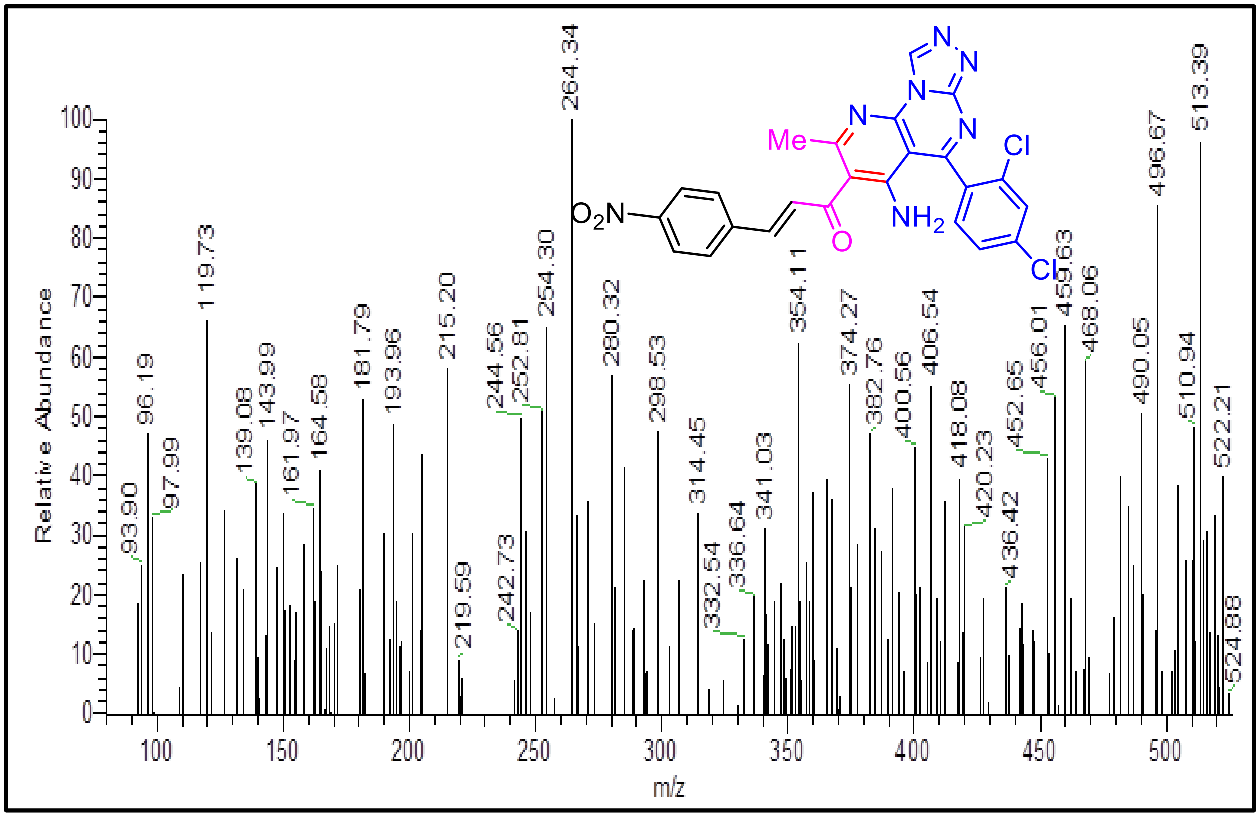

Mass spectrum of compound 5b

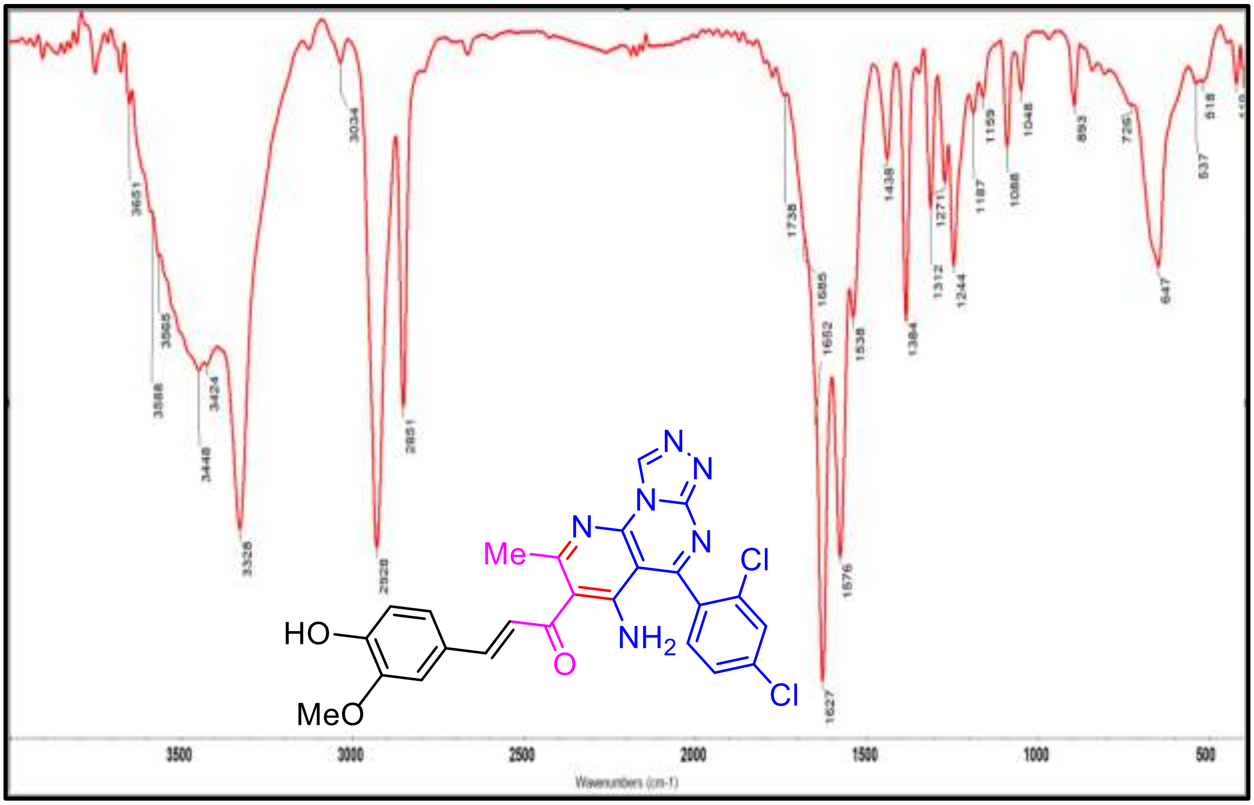

IR Spectrum of compound 5c

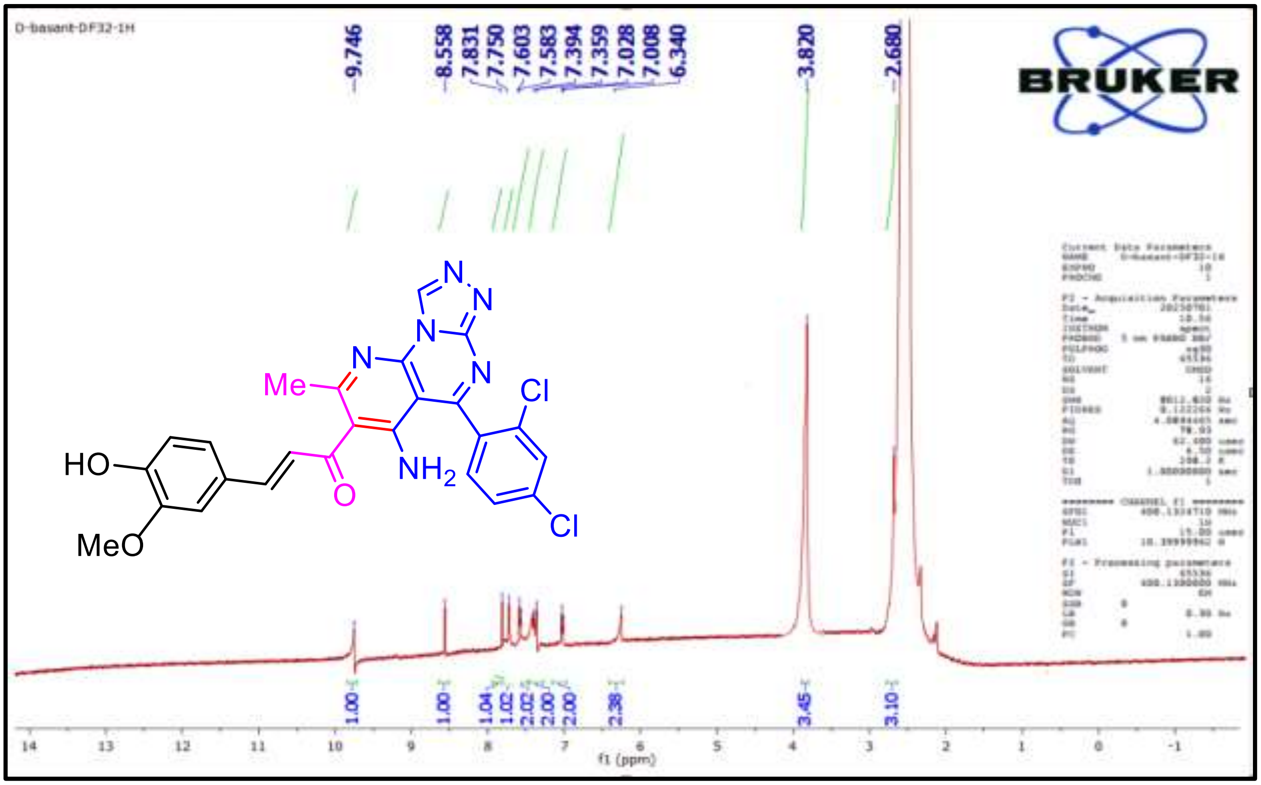

^{1}H-NMR Spectrum of compound 5c

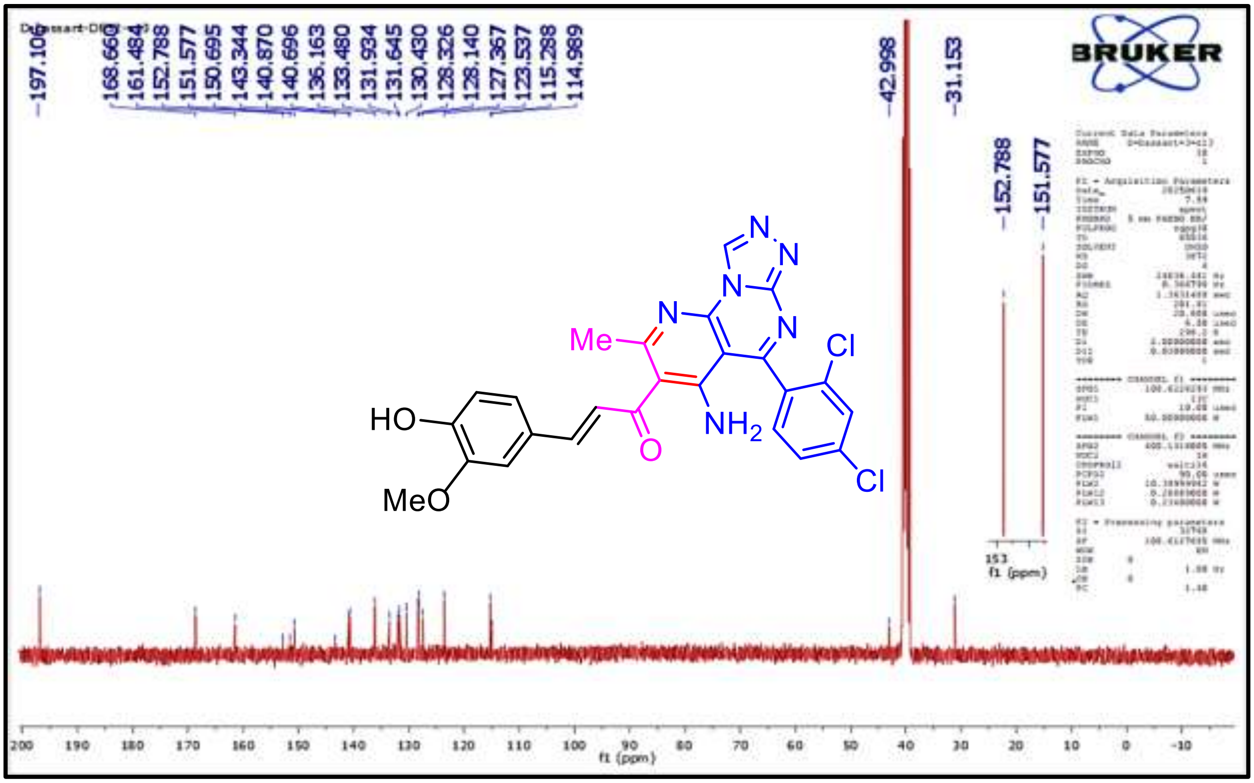

^{13}C-NMR Spectrum of compound 5c

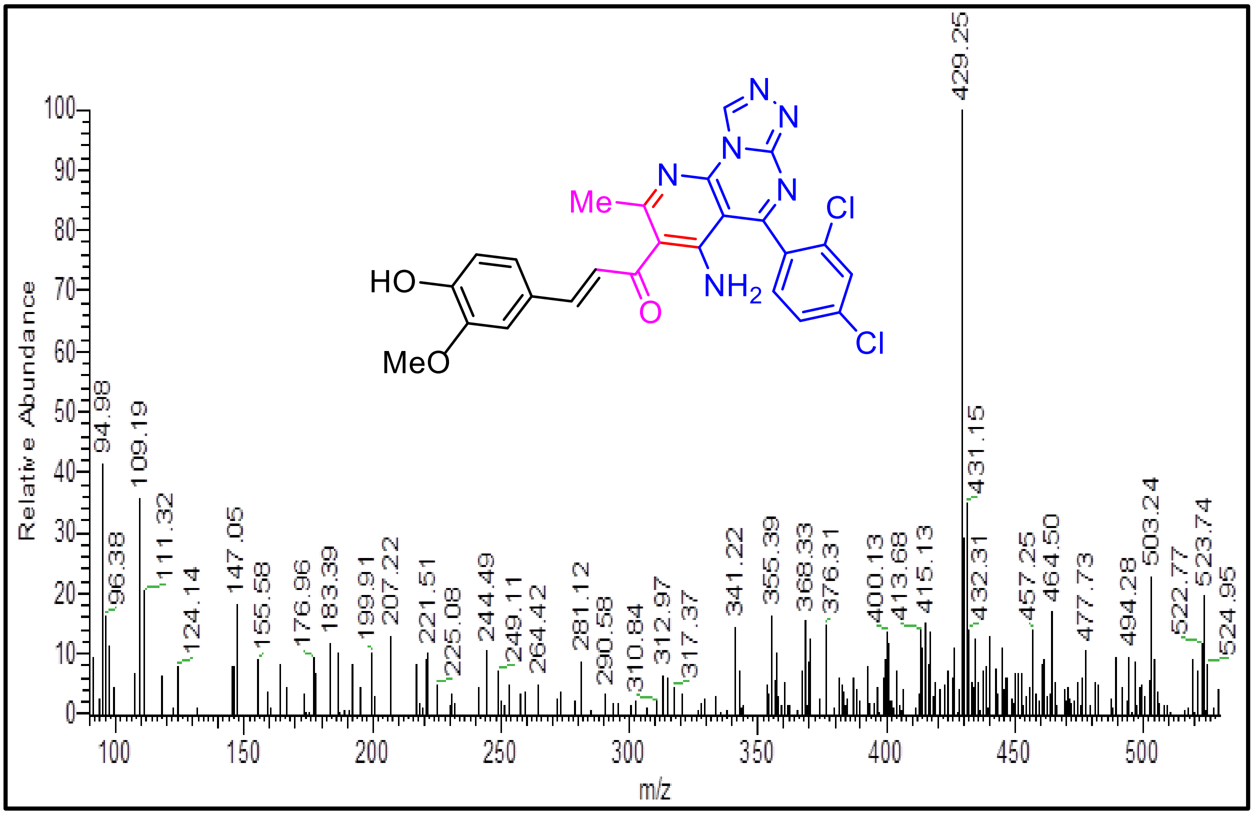

Mass spectrum of compound 5c

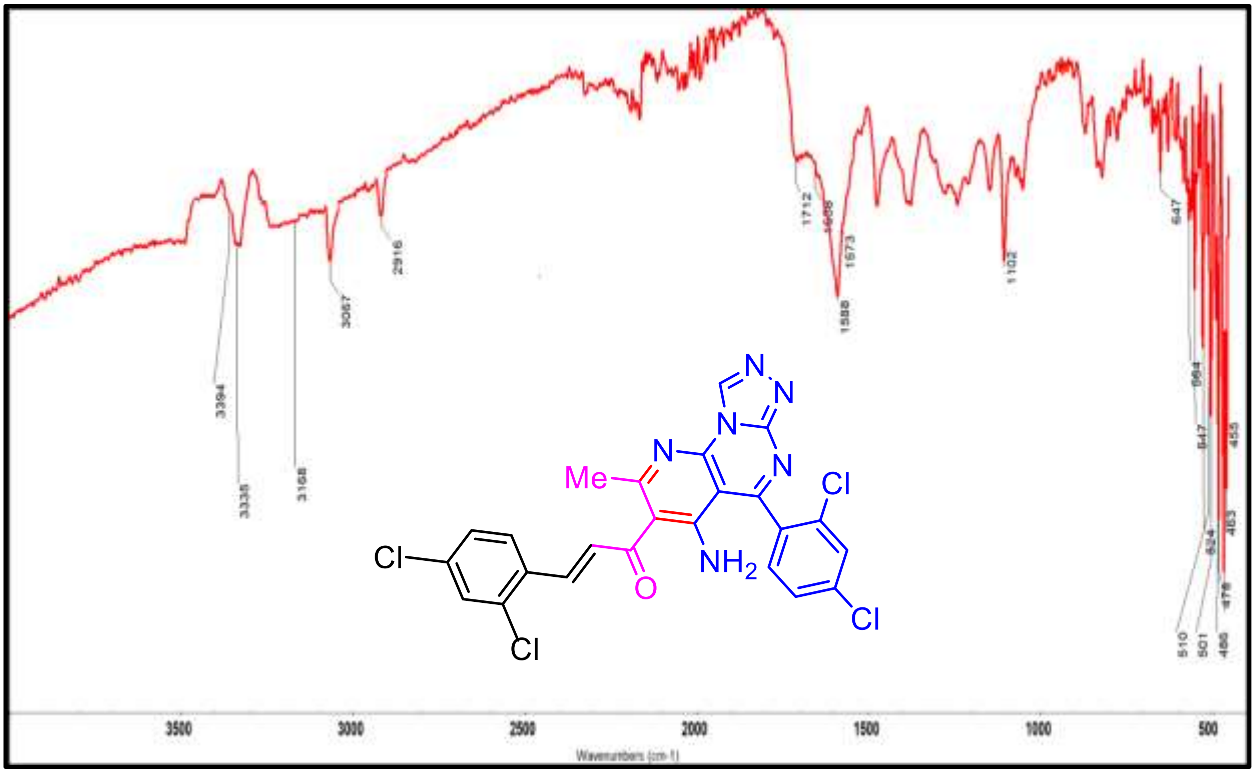

IR Spectrum of compound 5d

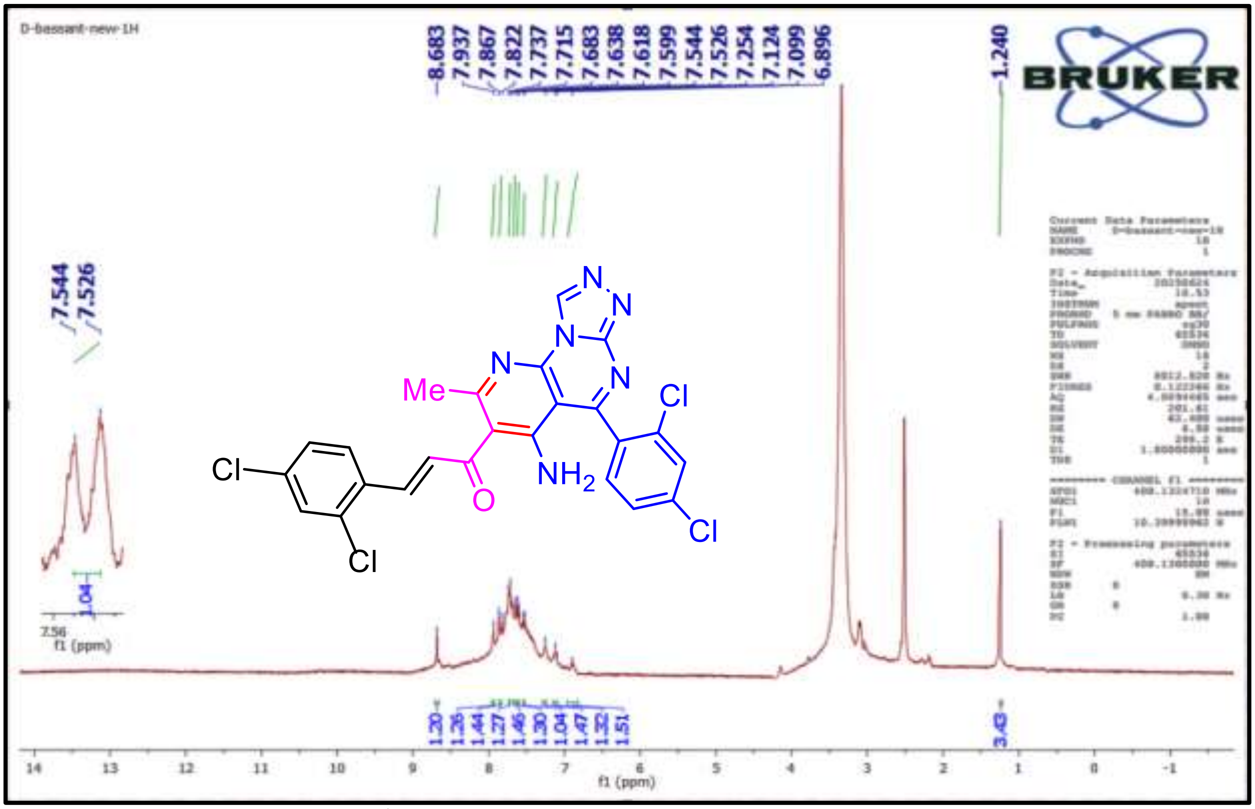

^{1}H-NMR Spectrum of compound 5d

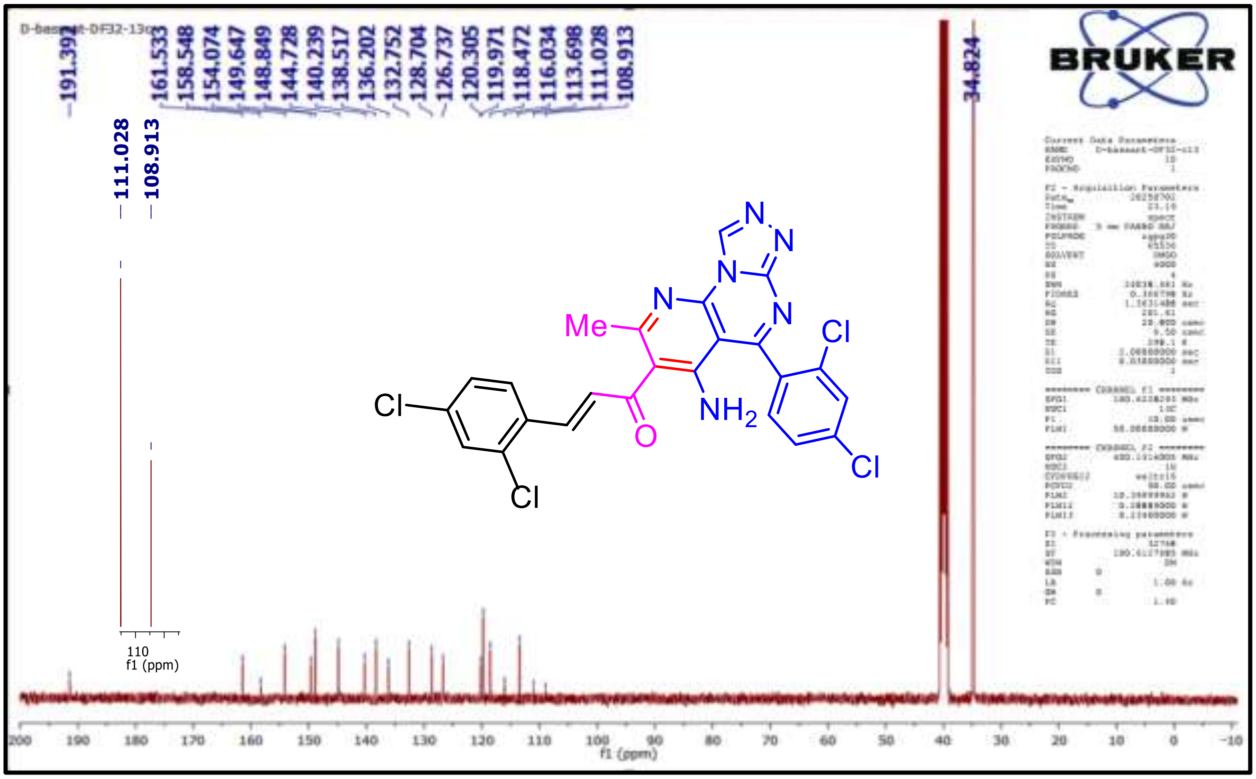

^{13}C-NMR Spectrum of compound 5d

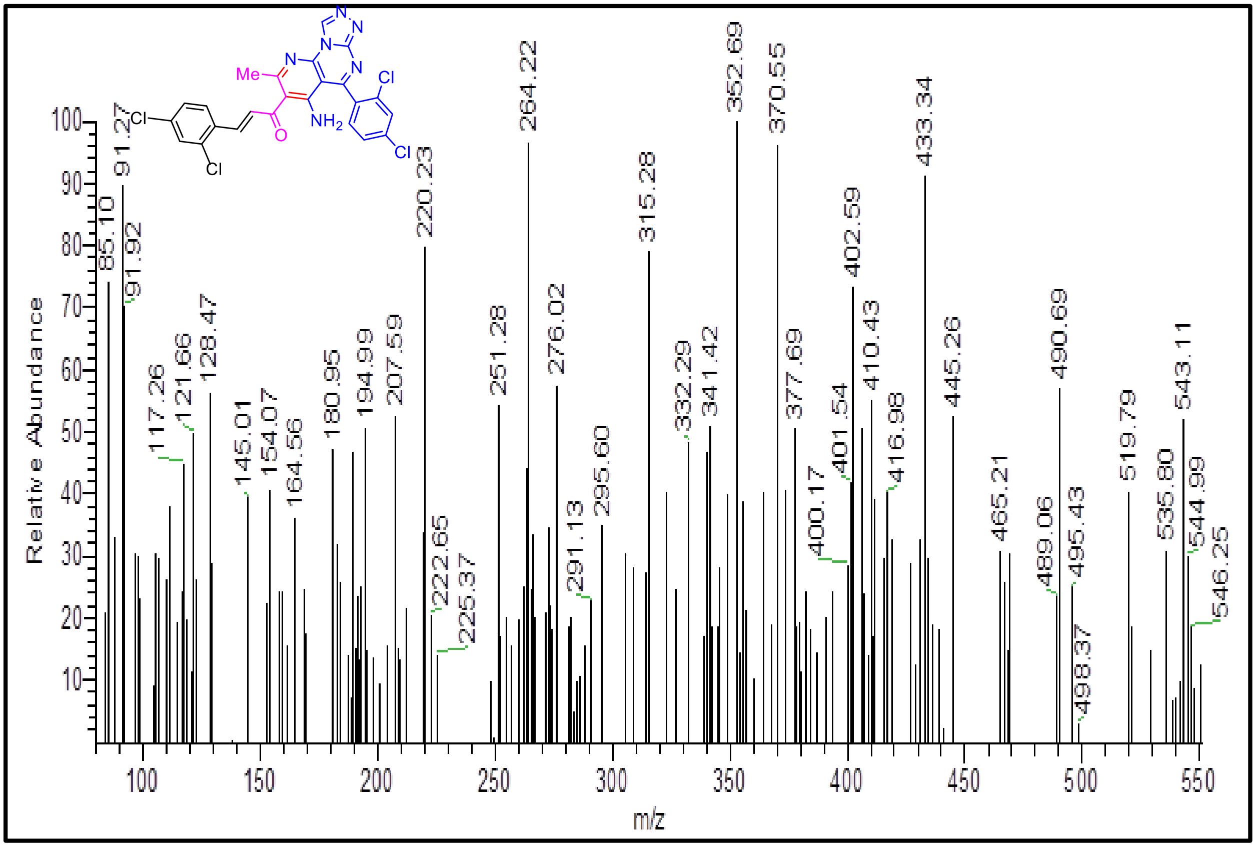

Mass spectrum of compound 5d

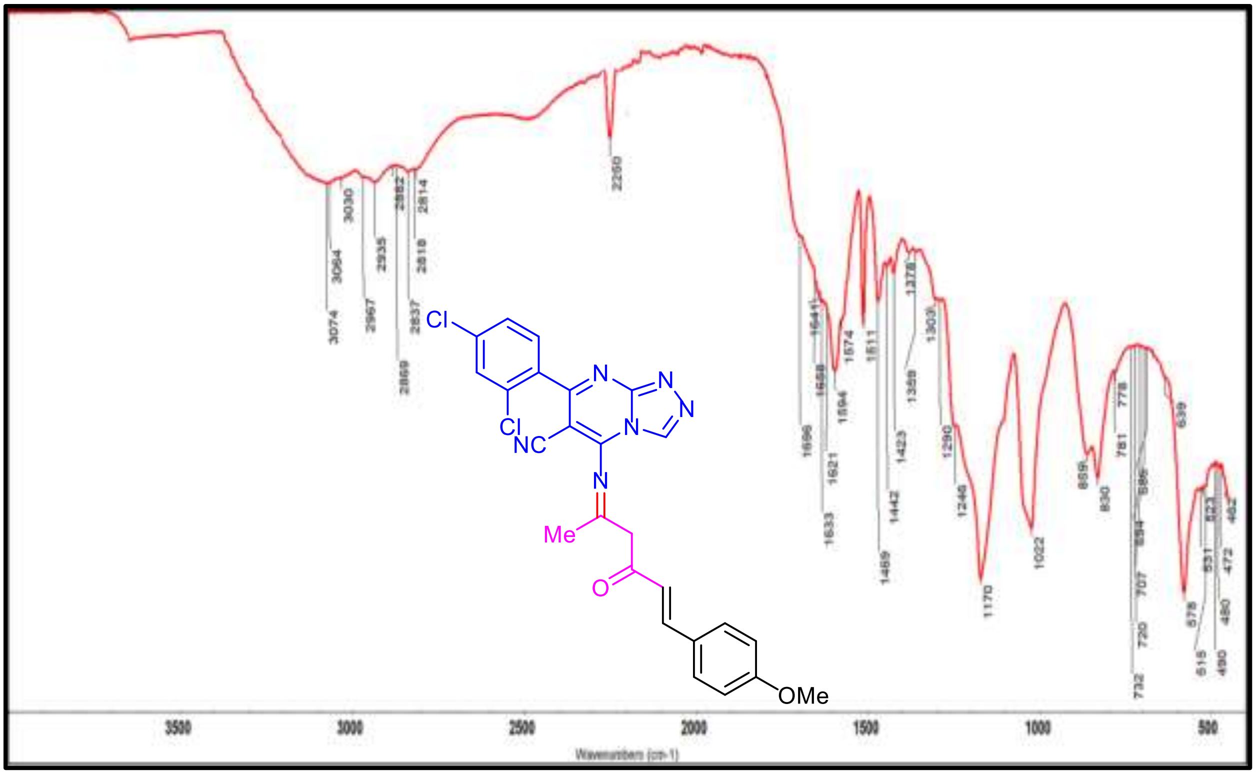

IR Spectrum of compound 6a

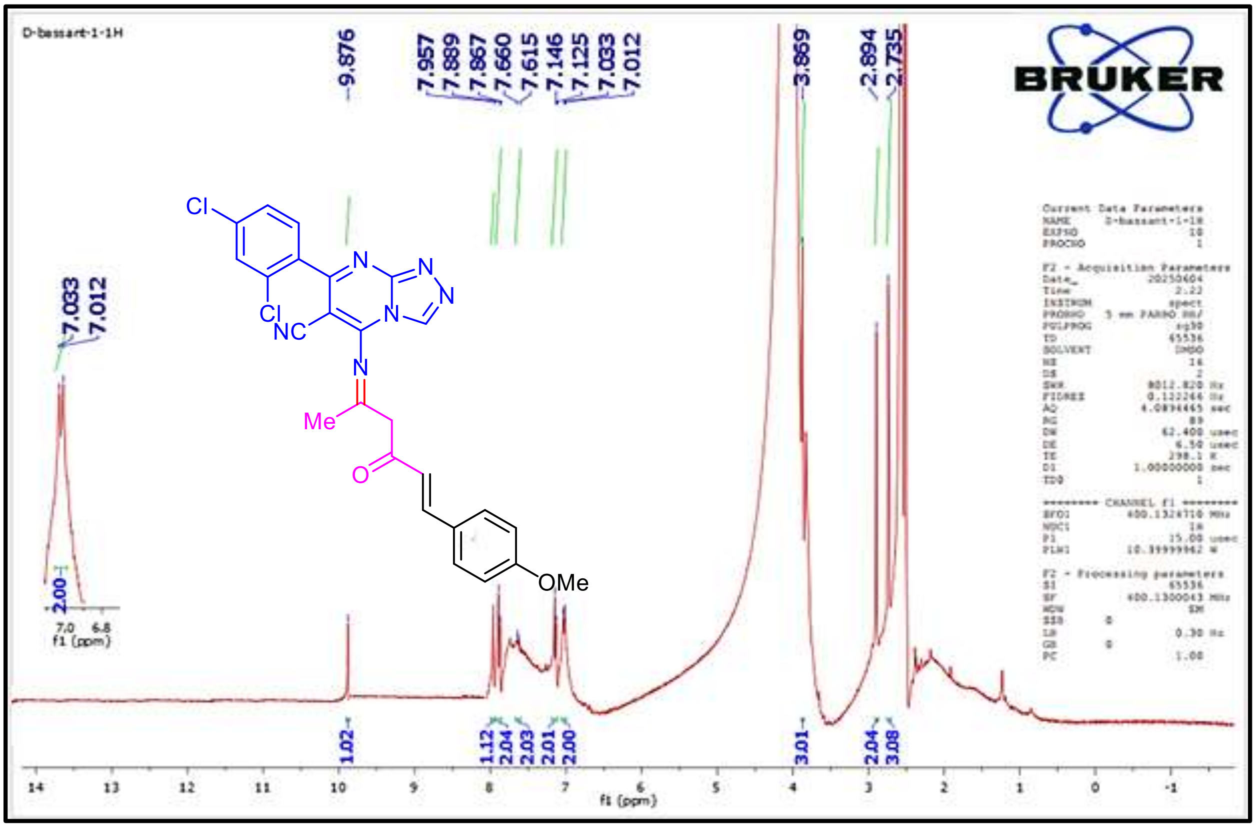

^{1}H-NMR Spectrum of compound 6a

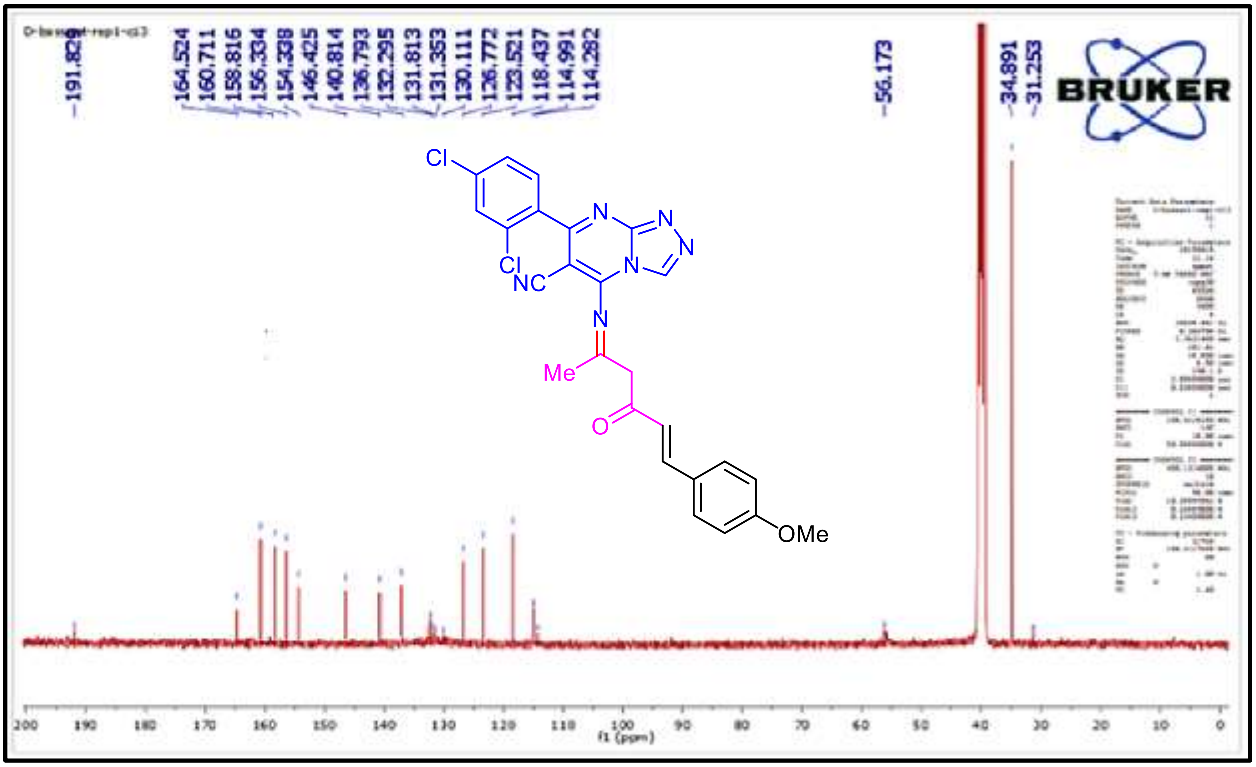

^{13}C-NMR Spectrum of compound 6a

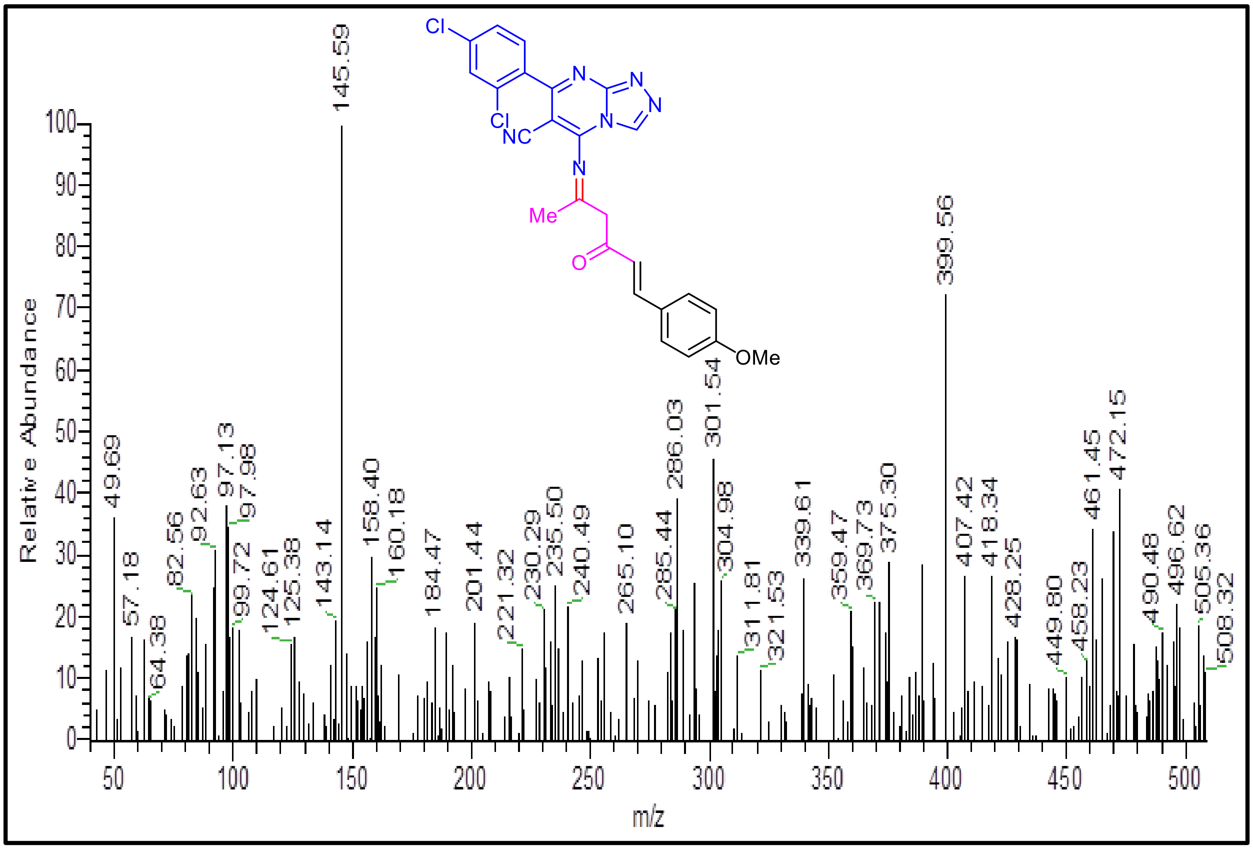

Mass spectrum of compound 6a

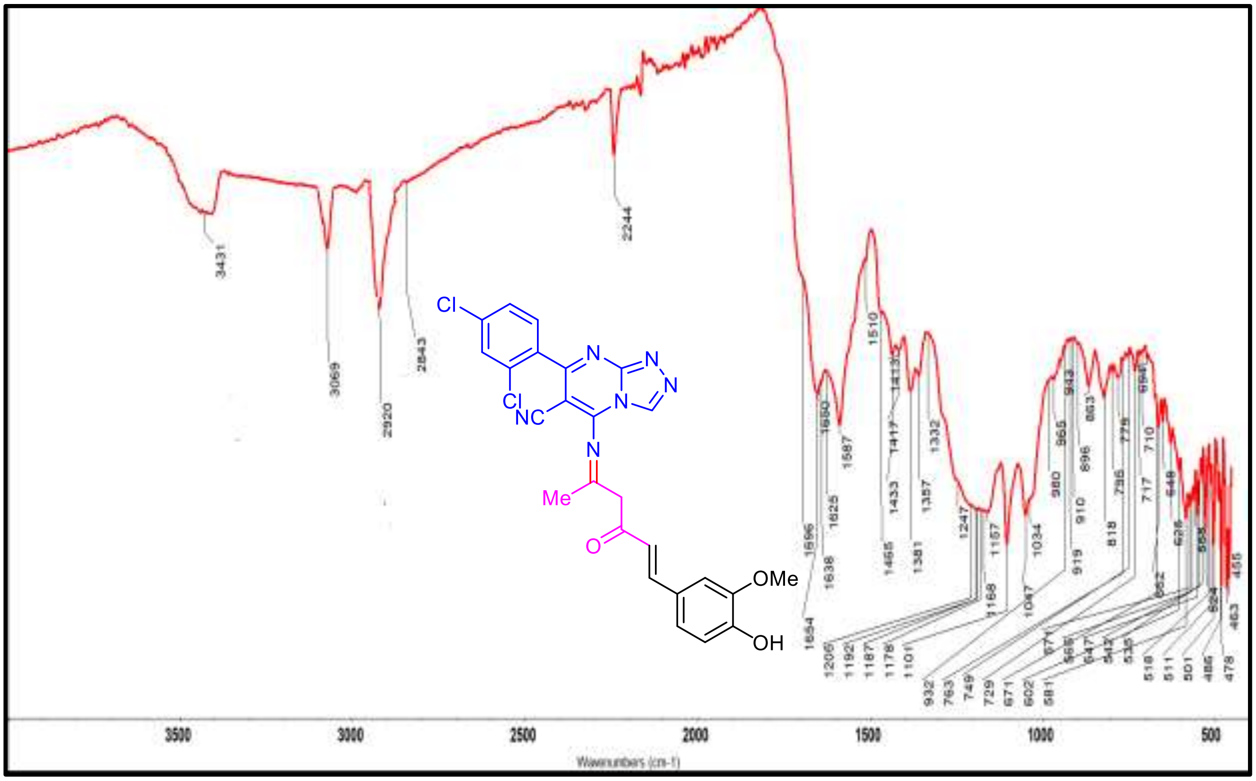

IR Spectrum of compound 6b

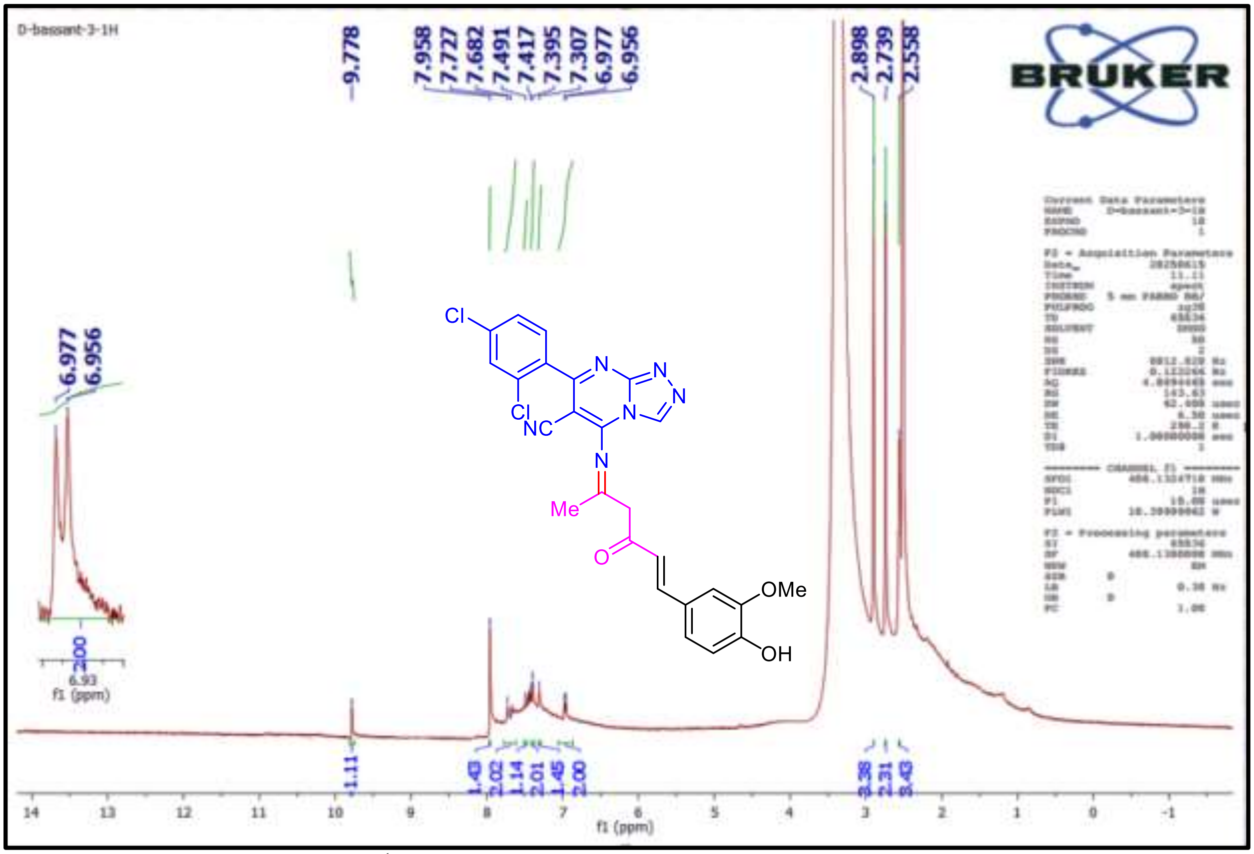

^{1}H-NMR Spectrum of compound 6b

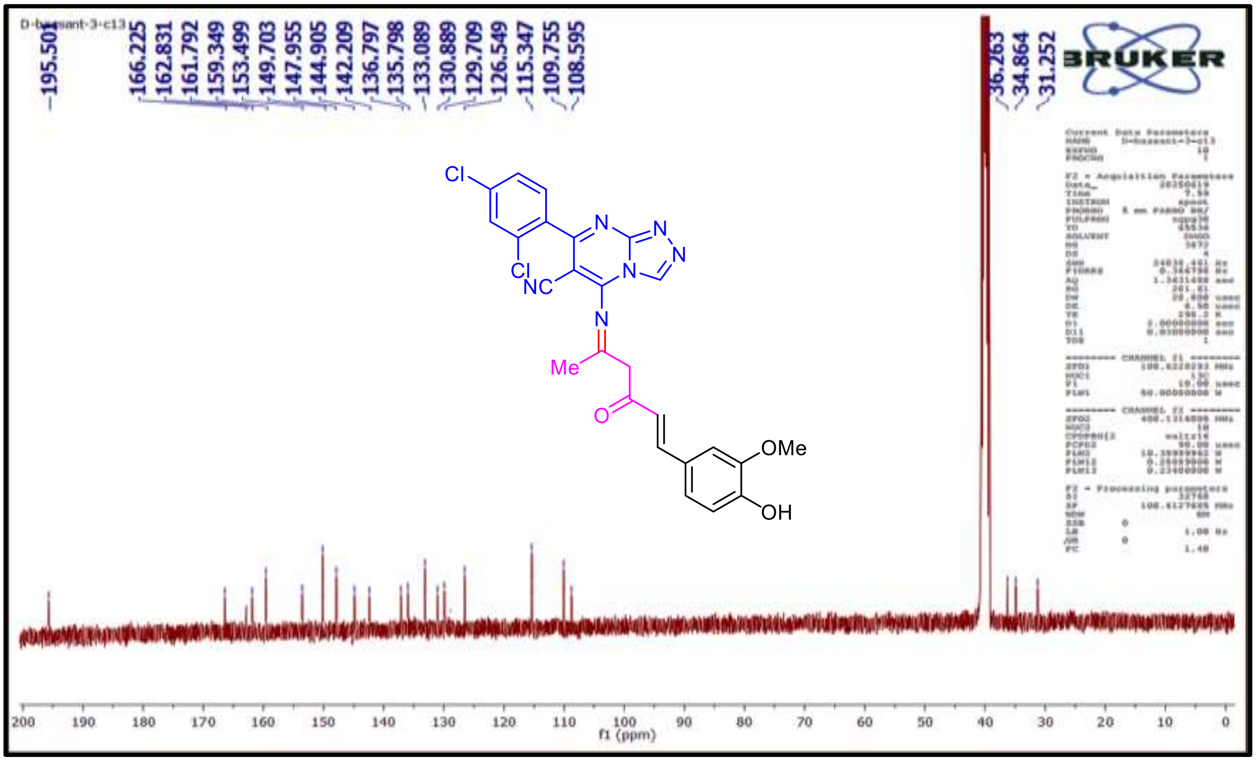

^{13}C-NMR Spectrum of compound 6b

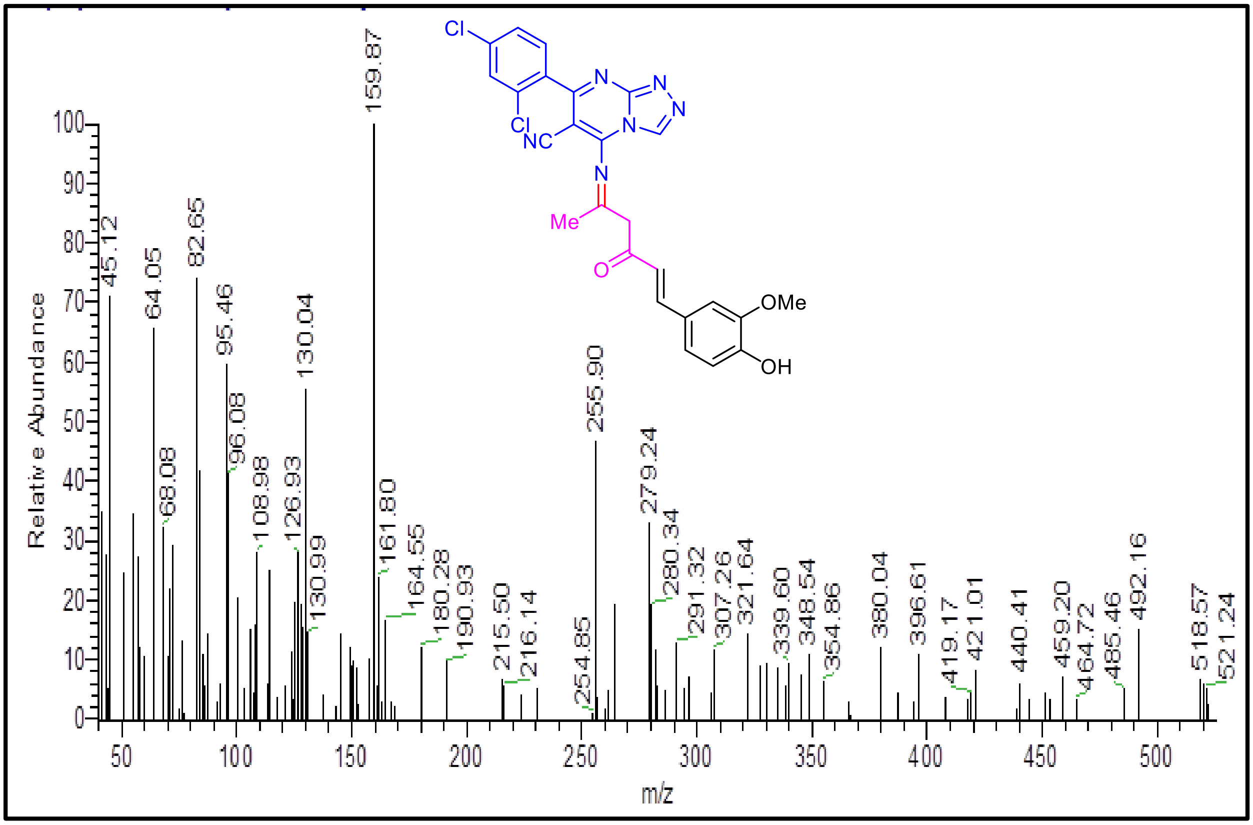

Mass spectrum of compound 6b

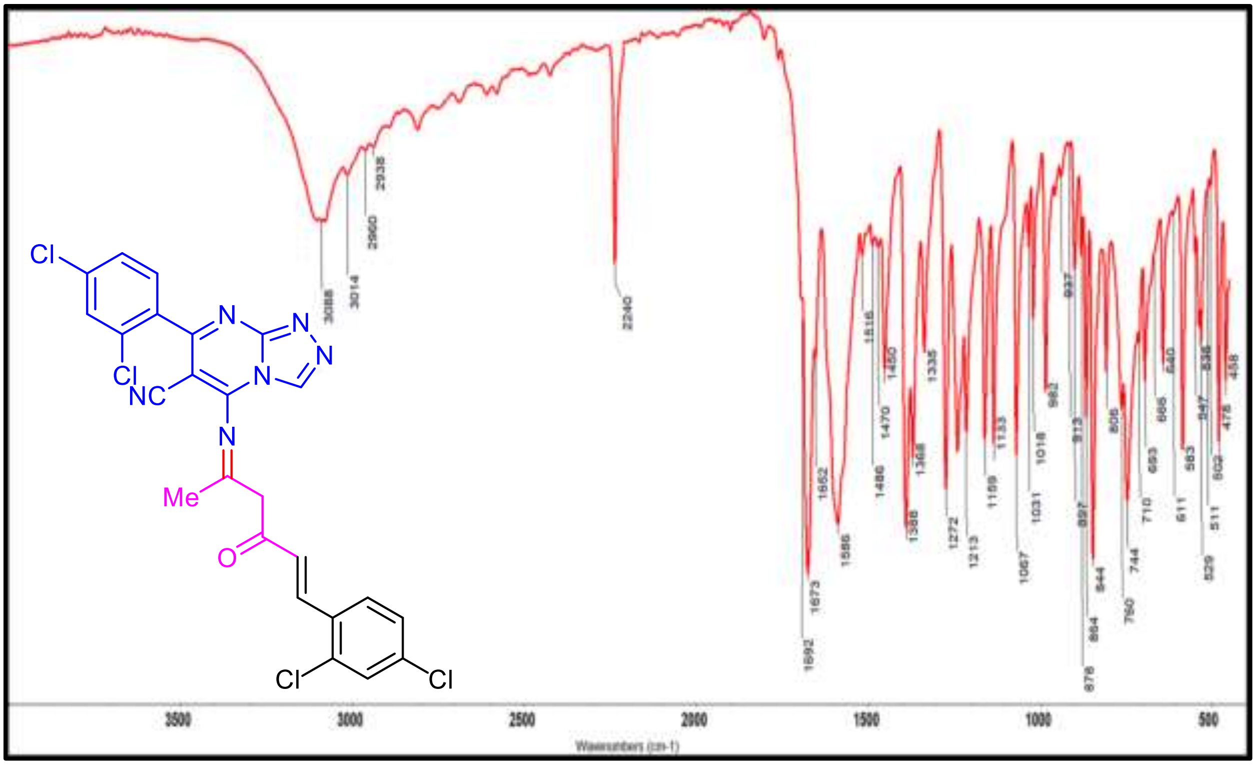

IR Spectrum of compound 6c

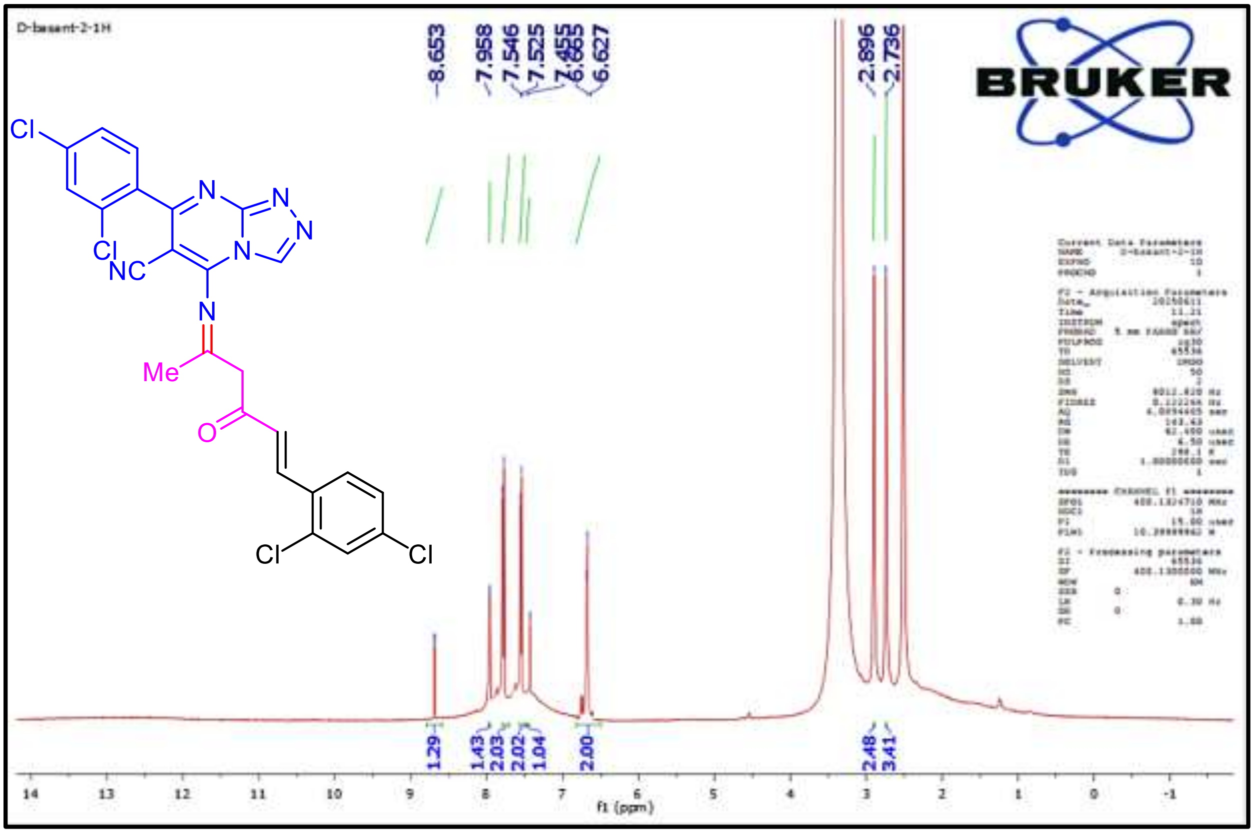

^{1}H-NMR Spectrum of compound 6c

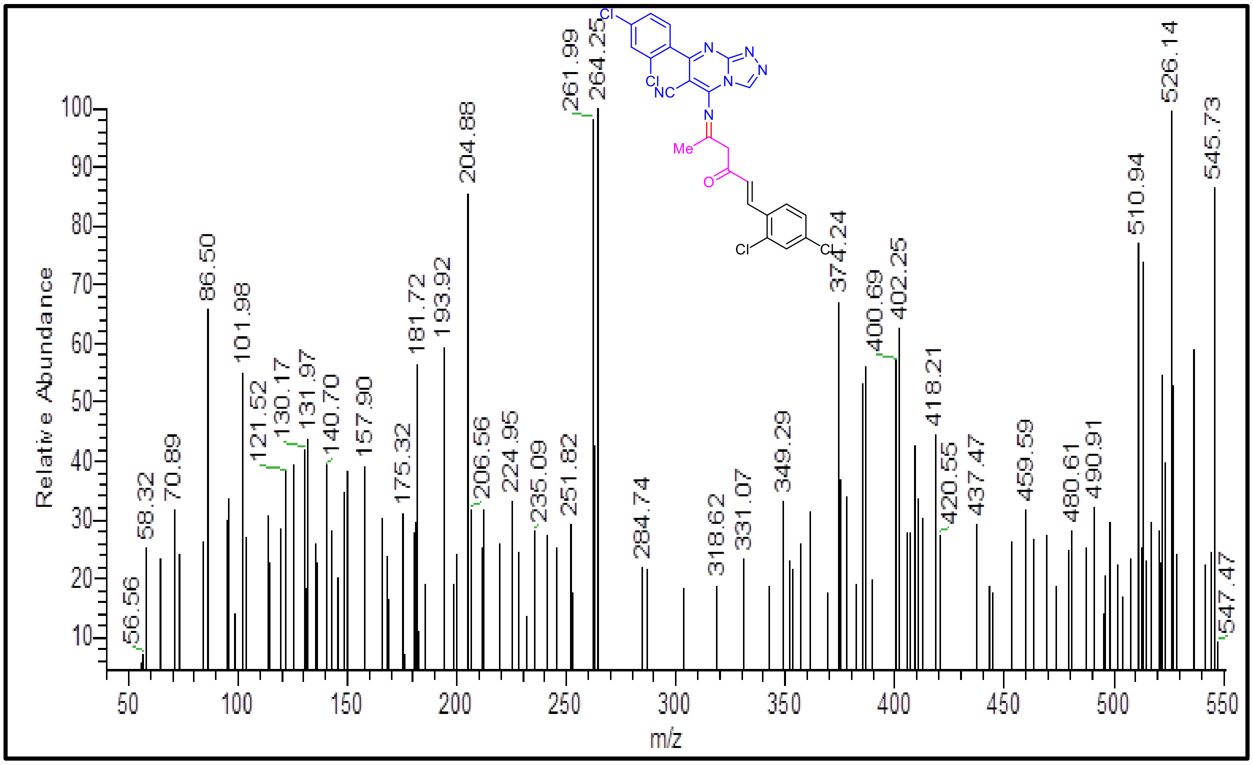

Mass spectrum of compound 6c

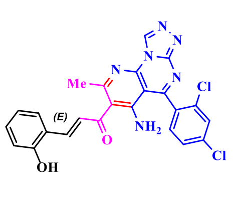
5a(E)

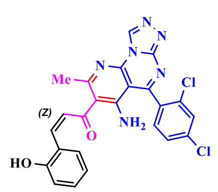
5a(Z)

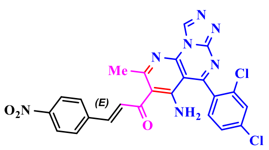
5b(E)

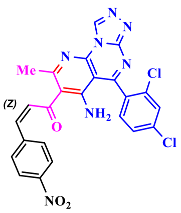
5b(Z)

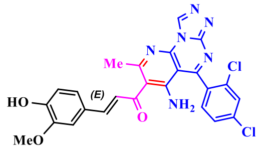
5c(E)

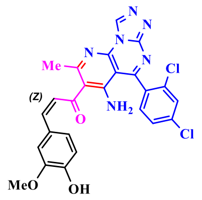
5c(Z)

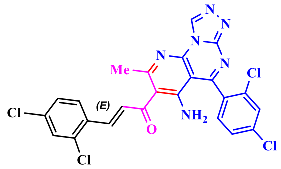
5d(E)

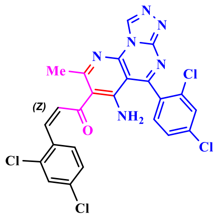
5d(Z)

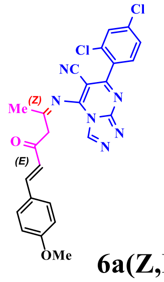
6a(Z,E)

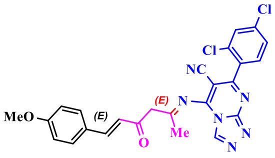
6a(E,E)

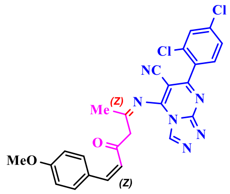
6a(Z,Z)

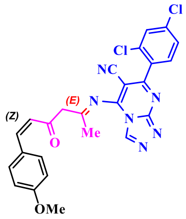
6a(E,Z)

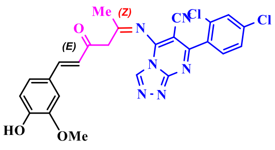
6b(Z,E)

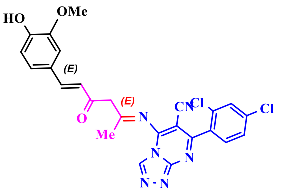
6b(E,E)

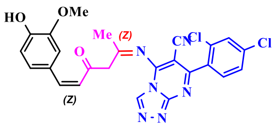
6b(Z,Z)

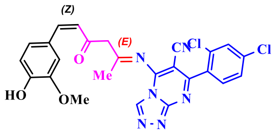
6b(E,Z)

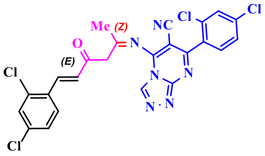
6c(Z,E)

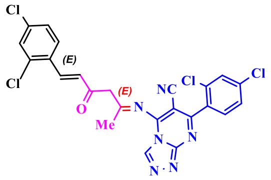
6c(E,E)

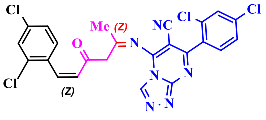
6c(Z,Z)

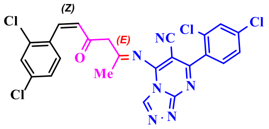
6c(E,Z)

Fig.S1: The structures of the synthesized compounds with their possible stereoisomers.

Compound	HOMO	LUMO	Energy Gap	Ionization Potential (IP)	Electron Affinity (Ea)	Electronic Chemical Potential (μ)	Chemical Hardness (η)	Chemical Softness (S)	Electrophilicity Index (ω)	Maximum Charge Acceptance (ΔN_{max})	Nucleophilicity index (N)
**Cpd5aE**	-5.946	-2.293	3.653	5.946	2.293	-4.120	1.827	0.547	4.645	2.255	2.685
**Cpd5aZ**	-5.966	-2.334	3.632	5.966	2.334	-4.150	1.816	0.551	4.742	2.285	2.665
**Cpd5bE**	-6.306	-2.943	3.363	6.306	2.943	-4.625	1.682	0.595	6.359	2.750	2.325
**Cpd5bZ**	-6.335	-2.919	3.416	6.335	2.919	-4.627	1.708	0.585	6.267	2.709	2.296
**Cpd5cE**	-5.579	-2.235	3.344	5.579	2.235	-3.907	1.672	0.598	4.565	2.337	3.052
**Cpd5cZ**	-5.629	-2.272	3.357	5.629	2.272	-3.951	1.679	0.596	4.649	2.353	3.002
**Cpd5dE**	-6.284	-2.515	3.769	6.284	2.515	-4.400	1.885	0.531	5.135	2.334	2.347
**Cpd5dZ**	-6.330	-2.338	3.992	6.330	2.338	-4.334	1.996	0.501	4.705	2.171	2.301
**Cpd6aEE**	-5.806	-2.100	3.706	5.806	2.100	-3.953	1.853	0.540	4.216	2.133	2.825
**Cpd6aEZ**	-5.879	-2.241	3.638	5.879	2.241	-4.060	1.819	0.550	4.531	2.232	2.752
**Cpd6aZE**	-5.813	-2.164	3.649	5.813	2.164	-3.989	1.825	0.548	4.359	2.186	2.818
**Cpd6aZZ**	-5.874	-2.055	3.819	5.874	2.055	-3.965	1.910	0.524	4.116	2.076	2.757
**Cpd6bEE**	-5.633	-2.132	3.501	5.633	2.132	-3.883	1.751	0.571	4.305	2.218	2.998
**Cpd6bEZ**	-5.655	-2.143	3.512	5.655	2.143	-3.899	1.756	0.569	4.329	2.220	2.976
**Cpd6bZE**	-5.605	-2.035	3.570	5.605	2.035	-3.820	1.785	0.560	4.088	2.140	3.026
**Cpd6bZZ**	-5.661	-2.061	3.600	5.661	2.061	-3.861	1.800	0.556	4.141	2.145	2.970
**Cpd6cEE**	-6.542	-2.286	4.256	6.542	2.286	-4.414	2.128	0.470	4.578	2.074	2.089
**Cpd6cEZ**	-6.545	-2.226	4.319	6.545	2.226	-4.386	2.160	0.463	4.453	2.031	2.086
**Cpd6cZE**	-6.515	-2.453	4.062	6.515	2.453	-4.484	2.031	0.492	4.950	2.208	2.116
**Cpd6cZZ**	-6.572	-2.270	4.302	6.572	2.270	-4.421	2.151	0.465	4.543	2.055	2.059
**Tetracyanoethylene (as Reference)**	-8.631	-4.389	4.242	8.631	4.389	-6.510	2.121	0.471	9.991	3.069	0.000

Table S1: Descriptors of global reactivity calculated at the DFT level B3LYP/RIJCOSX/6-31G(d,p) and solvent model CPCM (Water).

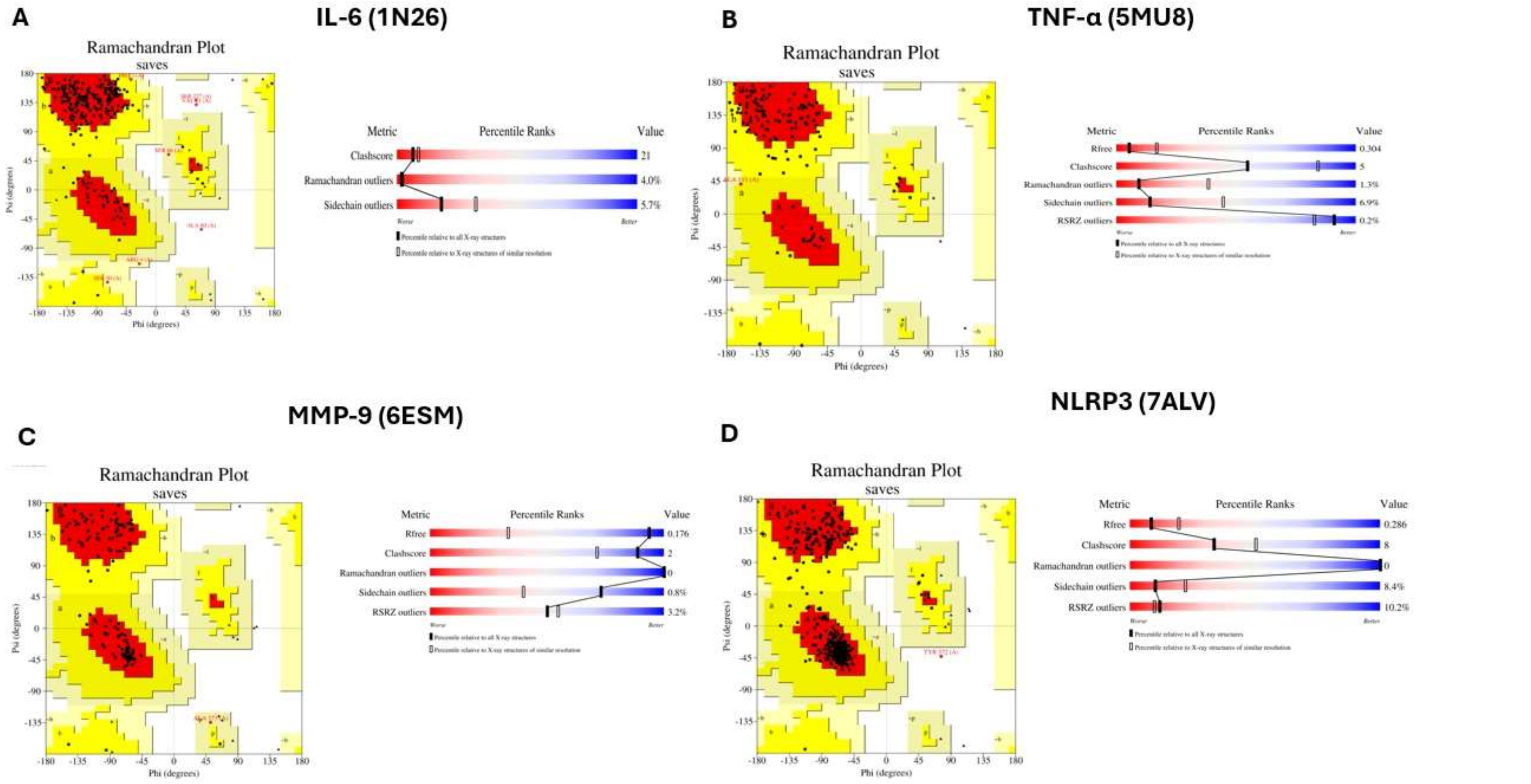

supplementary Figure 2S. Ramachandran plots of the four target protein structures used for molecular docking and molecular dynamics simulations. Ramachandran plots were generated using [PROCHECK] for the crystal structures of (A) IL-6 (PDB ID: 1N26, resolution 2.40 Å), (B) TNF-α (PDB ID: 5MU8, resolution 3.00 Å), (C) MMP-9 (PDB ID: 6ESM, resolution 1.10 Å), and (D) NLRP3 (PDB ID: 7ALV, resolution 2.83 Å). In each plot, the core (favored) regions are shown in dark green, allowed regions in light green, and generously allowed regions in pale yellow; outliers are indicated by red squares.
